# Controlled synchronization of a vibrating screen driven by two motors based on improved sliding mode controlling method

**DOI:** 10.1371/journal.pone.0294726

**Published:** 2023-11-21

**Authors:** Lei Jia, Guohui Wang, Cheng Pan, Ziliang Liu, Xin Zhang

**Affiliations:** School of Mechanical Engineering, Shenyang Ligong University, Shenyang, China; Chongqing Three Gorges University, CHINA

## Abstract

With a requirement of miniaturization in modern vibrating screens, the vibration synchronization method can no longer meet the process demand, so the controlled synchronization method is introduced in the vibrating screen to achieve zero phase error state and realize the purpose of increasing the amplitude. In this article, the controlled synchronization of a vibrating screen driven by two motors based on improved sliding mode controlling method is investigated. Firstly, according to the theory of mechanical dynamics, the motion state of the vibrating screen is simplified as the electromechanical coupling dynamical model of a vibrating system driven by two inductor motors. And then the synchronization conditions and stability criterion of the vibrating system are derived and numerically analyzed. Based on a master-slave controlling strategy, the controllers of two motors are respectively designed with Super-Twisting sliding mode control (ST-SMC) and backstepping second-order complementary sliding mode control (BSOCSMC), while the uncertainty is estimated by an adaptive radial basis function neural network (ARBFNN). In addition, Lyapunov stability analysis is performed on the two controllers to prove their stability theoretically. Finally, simulation analysis is conducted based on the dynamics model in this paper.

## 1. Introduction

Vibrating machinery is a common mechanical equipment in industrial production, which is used for material screening and conveying, such as vibrating screen, vibrating conveyor [1, 2]. With the improvement of science and technology, vibrating machines like vibrating screens no longer make use of the traditional rigid transmission method. They use the principle of vibration synchronization to force the motors on the vibrating screen to work at the same speed. Blekhman [3, 4] was the first to investigate the theory of vibration synchronization. He used two motors to drive two eccentric rotors (ERs) and mounted them on a shaking table. After certain conditions are met, the two motors can work synchronously. Wen et al. [5] have conducted in-depth research and development on vibration synchronization theory. They used the averaging method as well as Hamilton’s principle to derive the synchronization conditions of vibration systems and the stability conditions of synchronous working. In addition, they also proposed the theory of vibration synchronous transmission and space motion vibration synchronization, and also conducted an in-depth study of multi-frequency synchronization and controlled synchronization. Zhao et al. [6–9] proposed the criterion theory of the small parameter averaging method by adding disturbance parameters to vibration systems.

Vibrating screens by vibration synchronization have gained great economic benefits. However, for miniaturized vibrating screens, it is difficult for ERs to achieve zero phase error by vibration synchronization, which can affect screening efficiency and may even lead to clogging. Controlled synchronization is a good solution in order to meet miniaturization requirements. The theory of controlled synchronization has been applied by many scholars in different fields, proportional-integral-derivative (PID) and sliding mode control are two of the more mature control methods. Jia et al. [10, 11] investigated the multi-frequency synchronization problem of a multi-motor vibration system with fuzzy PID control and experimentally proved the feasibility and effectiveness of this method. In their research, fuzzy PID control has good controlling effect, but the response time is long and the robustness of fuzzy PID is not considered. Sliding mode control is a nonlinear control method with fast response and good robustness. Kong et al. [12, 13] designed a synchronization controller with adaptive sliding mode control based on master-slave control for the multi-motor compound synchronization. Furthermore, Fang et al [14] also used adaptive sliding mode control to design the synchronization controller. Zhang et al. [15] used adaptive sliding mode control for error tracking and synchronization control of the electro-hydraulic shaker, and the controller performance was excellent and robust. Adaptive sliding mode control is designed based on the convergence law, which is highly robust only in the sliding mode phase and does not consider the convergence performance in the arrival phase. In order to extend the range of robustness, Huang et al. [16–18] applied an adaptive global sliding mode control to the controlled synchronization for multiple motors under the action of materials, and achieved both speed and phase synchronization. Fang et al. [19] similarly designed an error controller by global sliding mode control, which was used to investigate the synchronization problem of a three-motor vibration system. The final simulation proves that the control method has better robustness. In addition, Xi et al. [20] designed a robust control algorithm for adaptive global sliding mode to control a class of chaotic synchronous systems. Although the adaptive global sliding mode control enhances the global robustness, the chattering phenomenon is not well addressed. However, intelligent control has great advantages in weakening the chattering and improving the robustness. In the research on position synchronization of manipulators, Zhai et al. [21] designed a neural network controller based on sliding mode control to estimate the uncertainty of the system online, which was able to significantly reduce the chattering. Shi et al. [22] combined fuzzy control with sliding mode control to design an adaptive fuzzy sliding mode controller for synchronous control of a spatial three-motor vibration system, and this control method reduced jitter and improved robustness.

Most of the investigations on synchronous control of motors are based on field oriented control, which has good control performance but complex structure. This paper is based on model predictive control, which is robust and simple, and then combines intelligent control with conventional control to improve the control performance of synchronous controllers. In section 2, the vibrating screen is transformed into a dynamics model of a vibrating system driven by two motors, and an electro-mechanical coupling model of the motor and the vibrating system is developed. In section 3, the synchronization conditions and stability conditions of the ERs are derived by using the small parameter method. In section 4, based on the master-slave control strategy, the controllers of two motors are designed separately by adopting the modified SMC and combining with ARBFNN, then the stability analysis of the controllers through Lyapunov theory is performed. In section 5, the synchronization and stability conditions are visualized and analyzed numerically, then the controlled synchronization is simulated to verify the effectiveness and robustness of controllers. Finally, section 6 shows some conclusions.

## 2. Dynamical model and induction motor model

### 2.1 Dynamical model of the vibrating system

**Fig 1:**
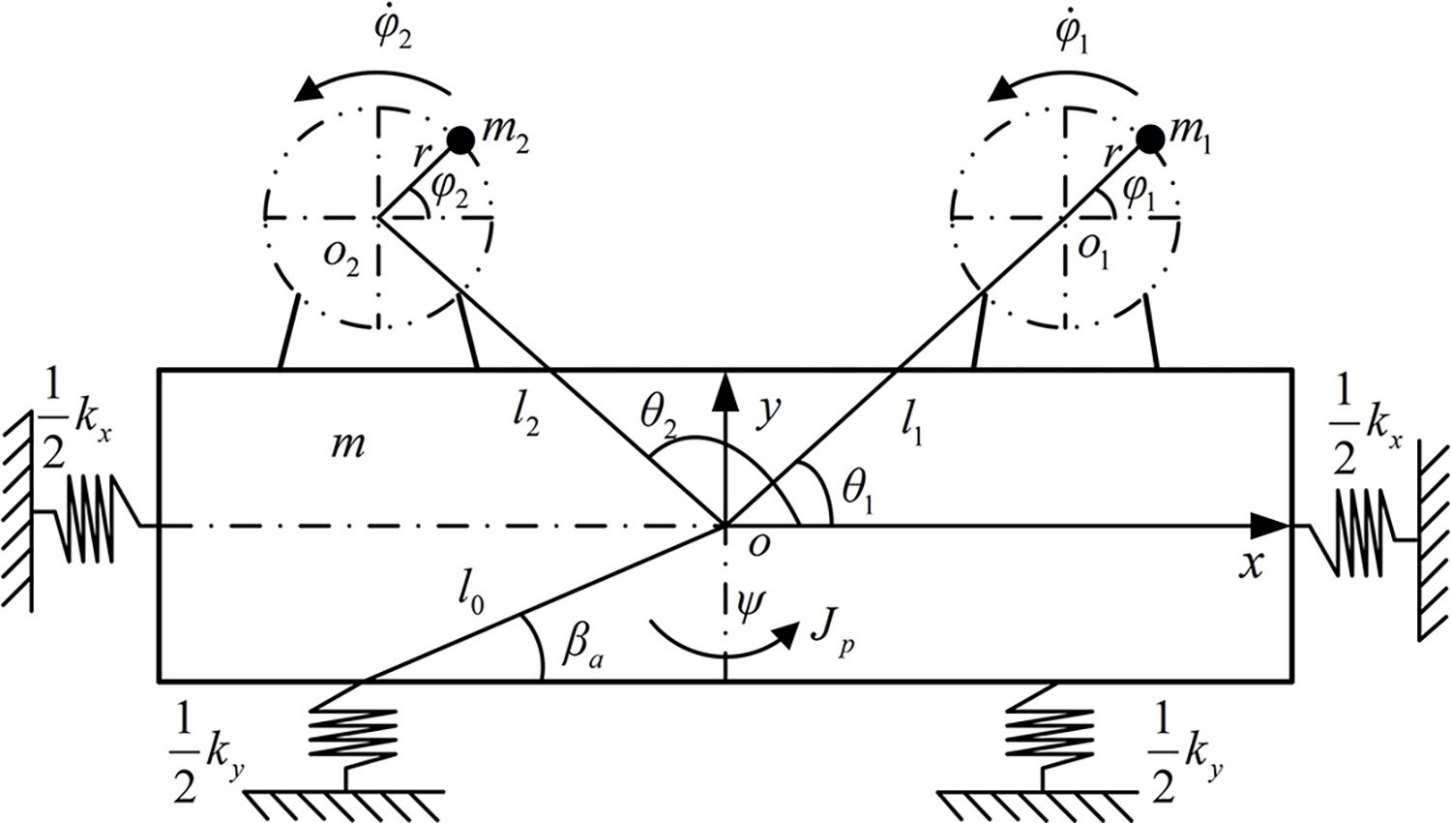
Mechanical model of a vibrating screen driven by two motors.

Fig 1 shows the equivalent mechanical model of a vibrating screen driven by two motors. According to Fig 1, the mathematical model of the vibration system can be established based on the Lagrange equation.

The kinetic energy of the vibrating system is as follows:

$$T=[m({\dot{x}}^{2}+{\dot{y}}^{2})+J_{p}{\dot{\psi }}^{2}+\sum_{i=1}^{2}\left(m_{i}({\dot{x}}_{i}{}^{2}+{\dot{y}}_{i}{}^{2})+J_{i}{\dot{\varphi }}_{i}{}^{2}\right)]/2$$ (1)

with

$$\left(\begin{array}{c} x_{i}\\ y_{i} \end{array})=(\begin{array}{c} x\\ y \end{array})+(\begin{array}{cc} 1 & -\psi \\ \psi & 1 \end{array})(\begin{array}{c} l_{i}\cos\theta_{i}+r\cos\varphi_{i}\\ l_{i}\sin\theta_{i}+r\sin\varphi_{i} \end{array}\right)$$

In Eq (1), *m* is the quality of the shaking table and motors. *J*_*p*_ is the rotational inertia of the shaking table. *m*_1_ and *m*_2_ are the masses of the two eccentric rotors (ERs) and the ERs are driven by motors. *J*_1_ and *J*_2_ are the rotational inertia of two motors. *x*_*i*_ and *y*_*i*_ are the coordinates for ERs. *l*_1_ and *l*_2_ represent the distance between *o* and *o*_1_, *o*_2_, *l*_1_ = *l*_2_. *θ*_1_ and *θ*_2_ are the position angles of two ERs. *r* indicates the radius of rotation of the ERs. *φ*_1_ and *φ*_2_ represent the phase of two ERs, *φ* = (*φ*_1_+*φ*_2_)/2 and 2*α* = (*φ*_1_−*φ*_2_).

The potential energy of the vibrating system is as follows:

$$V=k_{x}x^{2}/2+k_{y}y^{2}/2+k_{\psi }\psi^{2}/2$$ (2)

In Eq (2), *k*_*x*_, *k*_*y*_ and *k*_*ψ*_ are the spring stiffness of the vibration system in *x*, *y* and *ψ* directions, and $k_{\psi }=k_{x}{\left(l_{0}\sin\beta_{a}\right)}^{2}+k_{y}{\left(l_{0}\cos\beta_{a}\right)}^{2}$.

The dissipated energy of the vibration system is as follows:

$$D=f_{x}x^{2}+f_{y}y^{2}+f_{\psi }\psi^{2}$$ (3)

In Eq (3), *f*_*x*_, *f*_*y*_ and *f*_*ψ*_ are the damping coefficients of the vibration system in *x*, *y* and *ψ* directions, and $f_{\psi }=f_{x}{\left(l_{0}\sin\beta_{a}\right)}^{2}+f_{y}{\left(l_{0}\cos\beta_{a}\right)}^{2}$.

The Lagrange equation of the vibrating system is as follows:

$$\frac{d}{dt}\frac{\partial (T-V)}{\partial \dot{q}}-\frac{\partial (T-V)}{\partial q}+\frac{\partial D}{\partial \dot{q}}=Q$$ (4)

In Eq (4), ***q*** denotes generalized coordinates, and $q={\left(\begin{array}{ccccc} x & y & \psi & \varphi_{1} & \varphi_{2} \end{array}\right)}^{T}$. ***Q*** denotes generalized force, and $Q={\left(\begin{array}{ccccc} 0 & 0 & 0 & T_{e1} & T_{e2} \end{array}\right)}^{T}$. *T*_*e*1_ and *T*_*e*2_ denote the electromagnetic torque in ***Q***.

Taking Eqs (1)–(3) into Eq (4) and simplifying them, the mathematical model of the vibration system can be obtained as follows:

$$\begin{array}{l} M\ddot{x}+f_{x}\dot{x}+k_{x}x=\sum_{i=1}^{2}m_{i}r{\dot{\varphi }}_{i}{}^{2}\cos\varphi_{i}\\ M\ddot{y}+f_{y}\dot{y}+k_{y}y=\sum_{i=1}^{2}m_{i}r{\dot{\varphi }}_{i}{}^{2}\sin\varphi_{i}\\ J\ddot{\psi }+f_{\psi }\dot{\psi }+k_{\psi }\psi =\sum_{i=1}^{2}m_{i}rl_{i}{\dot{\varphi }}_{i}{}^{2}\sin(\varphi_{i}-\theta_{i})\\ J_{1}{\ddot{\varphi }}_{1}+f_{1}{\dot{\varphi }}_{1}=T_{e1}-T_{L1}\\ J_{2}{\ddot{\varphi }}_{2}+f_{2}{\dot{\varphi }}_{2}=T_{e2}-T_{L2} \end{array}$$ (5)

*T*_*L*1_ and *T*_*L*2_ are indicated as:

$$\begin{array}{c} T_{L1}=m_{1}r[\ddot{y}\cos\varphi_{1}-\ddot{x}\sin\varphi_{1}+l_{1}{\dot{\psi }}^{2}\sin(\varphi_{1}-\theta_{1})+l_{1}\ddot{\psi }\cos(\varphi_{1}-\theta_{1})]\\ T_{L2}=m_{2}r[\ddot{y}\cos\varphi_{2}-\ddot{x}\sin\varphi_{2}+l_{2}{\dot{\psi }}^{2}\sin(\varphi_{2}-\theta_{2})+l_{2}\ddot{\psi }\cos(\varphi_{2}-\theta_{2})] \end{array}$$ (6)

In Eq (5), *M* indicates the total mass, *M* = *m*+*m*_1_+*m*_2_. *J* denotes the total rotational inertia of the vibrating system, $J=Ml_{e}{}^{2}\approx J_{p}+m_{1}(l_{1}{}^{2}+r^{2})+m_{2}(l_{2}{}^{2}+r^{2})$. *T*_*L*1_ and *T*_*L*2_ denote load torque.

### 2.2 Equation of state for induction motor

*ω*−***ϕ***_*r*_−***i***_*s*_ is selected as the state variable to control the motor, so the equation of state of the induction motor in coordinate *αβ* can be obtained as:

$$\begin{array}{l} {\dot{\phi }}_{r\alpha }=-\phi_{r\alpha }/T_{r}-n_{p}\omega /\phi_{r\beta }+L_{m}i_{s\alpha }/T_{r}\\ {\dot{\phi }}_{r\beta }=n_{p}\omega /\phi_{r\alpha }-\phi_{r\beta }/T_{r}+L_{m}i_{s\beta }/T_{r}\\ {\dot{i}}_{s\alpha }=L_{m}\phi_{r\alpha }/(\sigma L_{s}T_{r}L_{r})+L_{m}n_{p}\omega \phi_{r\beta }/(\sigma L_{s}L_{r})-(L_{m}{}^{2}+R_{s}L_{r}T_{r})i_{s\beta }/(\sigma L_{s}T_{r}L_{r})+u_{s\alpha }/(\sigma L_{s})\\ {\dot{i}}_{s\beta }=-L_{m}n_{p}\omega \phi_{r\alpha }/(\sigma L_{s}L_{r})+L_{m}\phi_{r\beta }/(\sigma L_{s}L_{r}T_{r})-(L_{m}{}^{2}+R_{s}L_{r}T_{r})i_{s\beta }/(\sigma L_{s}T_{r}L_{r})+u_{s\beta }/(\sigma L_{s}) \end{array}$$ (7)

Where, *ω* is the mechanical angular speed of the motor. *i*_*sα*_ and *i*_*sβ*_ are the stator current in coordinate *αβ*. *ϕ*_*rα*_ and *ϕ*_*rβ*_ are the rotor magnetic chains in coordinate *αβ*. *u*_*sα*_ and *u*_*sβ*_ are the stator voltage in coordinate *αβ*. *L*_*s*_ and *R*_*s*_ are respectively indicates stator inductance and stator resistance. *T*_*r*_ denotes the rotor time constant, *T*_*r*_ = *L*_*r*_/*R*_*r*_. *L*_*r*_ and *L*_*m*_ are respectively denotes rotor inductance and mutual inductance coefficients. *σ* is magnetic leakage coefficient. *σ* = 1−*L*_*m*_^2^/(*L*_*s*_*L*_*r*_).

Based on ***ϕ***_*r*_ and ***i***_*s*_ we can calculate ***ϕ***_*s*_ as:

$$\phi_{s}=(L_{m}/L_{r})\phi_{r}-(L_{r}-L_{s}L_{r}{}^{2}/L_{m}{}^{2})i_{s}$$ (8)

The electromagnetic torque of the induction motor can be obtained as:

$$T_{e}=(3/2)n_{p}\phi_{s}⊗i_{s}$$ (9)

Where, ⊗ denotes a fork product.

## 3. Synchronization conditions and stability conditions

When the vibration system is running steadily, the responses in *x*, *y* and *ψ* directions can be obtained according to Eq (5).

$$\begin{array}{l} x=-r_{m}r/\mu_{x}[\cos(\varphi_{1}+\gamma_{x})+\eta \cos(\varphi_{2}+\gamma_{x})]\\ y=-r_{m}r/\mu_{y}[\sin(\varphi_{1}+\gamma_{y})+\eta \sin(\varphi_{2}+\gamma_{y})]\\ \psi =-(r_{m}rr_{l}/l_{e}\mu_{\psi })[\sin(\varphi_{1}-\theta_{1}+\gamma_{\psi })+\eta \sin(\varphi_{2}-\theta_{2}+\gamma_{\psi })] \end{array}$$ (10)

Where, $\mu_{i}=1-\omega_{x}{}^{2}/\omega_{0}{}^{2}(i=x,y,\psi )$, *r*_*l*_ = *l*_1_/*l*_*e*_, $\zeta_{i}=f_{i}/(2\sqrt{k_{i}M})(i=x,y)$, $l_{e}{}^{2}=J/M$, $\tan\gamma_{i}=2\zeta_{i}\omega_{i}/\omega_{0}\mu_{i}(i=x,y,\psi )$, $\omega_{i}{}^{2}=k_{i}/M(i=x,y)$, $\omega_{\psi }{}^{2}=k_{\psi }/J$, $\zeta_{\psi }=f_{\psi }/2\sqrt{k_{\psi }J}$, *r*_*m*_ = *m*_1_/*M*, *η* = *m*_2_/*m*_1_. *ω*_0_ indicates the average angular velocity of ERs, $\omega_{0}=\int_{0}^{T_{0}}\dot{\varphi }dt/T_{0}$.

Introducing perturbation parameters into the vibration system, then ${\dot{\varphi }}_{1}=(1+\varepsilon_{1})\omega_{0}$, ${\dot{\varphi }}_{2}=(1+\varepsilon_{2})\omega_{0}$, ${\ddot{\varphi }}_{1}={\dot{\varepsilon }}_{1}\omega_{0}$, ${\ddot{\varphi }}_{2}={\dot{\varepsilon }}_{2}\omega_{0}$. Taking Eq (10) into Eqs (5) and (6), then Eq (11) can be obtained by using the small parameter averaging method and integrating.

$$\begin{array}{l} J_{1}{\dot{\bar{\varepsilon }}}_{1}\omega_{0}+f_{1}\omega_{0}(1+{\bar{\varepsilon }}_{1})={\bar{T}}_{e1}-{\bar{T}}_{L1}\\ J_{2}{\dot{\bar{\varepsilon }}}_{2}\omega_{0}+f_{2}\omega_{0}(1+{\bar{\varepsilon }}_{2})={\bar{T}}_{e2}-{\bar{T}}_{L2} \end{array}$$ (11)

$$\begin{array}{l} {\bar{T}}_{L1}=m_{1}r^{2}\omega_{0}(a_{11}{\dot{\bar{\varepsilon }}}_{1}+a_{12}{\dot{\bar{\varepsilon }}}_{2}+b_{11}{\bar{\varepsilon }}_{1}+b_{12}{\bar{\varepsilon }}_{2}+\kappa_{1})\\ {\bar{T}}_{L2}=m_{1}r^{2}\omega_{0}(a_{21}{\dot{\bar{\varepsilon }}}_{1}+a_{22}{\dot{\bar{\varepsilon }}}_{2}+b_{21}{\bar{\varepsilon }}_{1}+b_{22}{\bar{\varepsilon }}_{2}+\kappa_{2}) \end{array}$$ (12)

with

$$\kappa_{1}=(b_{11}+b_{12})/2,\;\kappa_{2}=(b_{21}+b_{22})/2$$

$$a_{11}=-[r_{m}\cos\gamma_{x}/\mu_{x}+r_{m}\cos\gamma_{y}/\mu_{y}+r_{m}r_{l}{}^{2}\cos\gamma_{\psi }/\mu_{\psi }]/2$$

$$a_{12}=-\eta [r_{m}r_{l}{}^{2}\cos(-2\alpha +\theta_{1}-\theta_{2}+\gamma_{\psi })/\mu_{\psi }+r_{m}\cos(-2\alpha +\gamma_{x})/\mu_{x2}+r_{m}\cos(-2\alpha +\gamma_{y})/\mu_{y2}]/2$$

$$b_{11}=\omega_{0}[r_{m}\sin\gamma_{x}/\mu_{x}+r_{m}\sin\gamma_{y}/\mu_{y}+r_{m}r_{l}{}^{2}\sin\gamma_{\psi }/\mu_{\psi }]$$

$$b_{12}=\eta \omega_{0}[r_{m}r_{l}{}^{2}\sin(-2\alpha +\theta_{1}-\theta_{2}+\gamma_{\psi })/\mu_{\psi }+r_{m}\sin(-2\alpha +\gamma_{x})/\mu_{x}+r_{m}\sin(-2\alpha +\gamma_{y})/\mu_{y}]$$

$$a_{21}=-\eta [r_{m}r_{l}{}^{2}\cos(2\alpha -\theta_{1}+\theta_{2}+\gamma_{\psi })/\mu_{\psi }+r_{m}\cos(2\alpha +\gamma_{x})/\mu_{x}+r_{m}\cos(2\alpha +\gamma_{y})/\mu_{y}]/2$$

$$a_{22}=-\eta^{2}[r_{m}\cos\gamma_{x}/\mu_{x}+r_{m}\cos\gamma_{y}/\mu_{y}+r_{m}r_{l}{}^{2}\cos\gamma_{\psi }/\mu_{\psi }]/2$$

$$b_{21}=\eta \omega_{0}[r_{m}r_{l}{}^{2}\sin(2\alpha -\theta_{1}+\theta_{2}+\gamma_{\psi })/\mu_{\psi }+r_{m}\sin(2\alpha +\gamma_{x})/\mu_{x}+r_{m}\sin(2\alpha +\gamma_{y})/\mu_{y}]$$

$$b_{22}=\eta^{2}\omega_{0}[r_{m}\sin\gamma_{x}/\mu_{x}+r_{m}\sin\gamma_{y}/\mu_{y}+r_{m}r_{l}{}^{2}\sin\gamma_{\psi }/\mu_{\psi }]$$

According to Ref. [6], the electromagnetic torque when the vibration system reaches a steady state is Eq (13).

$${\bar{T}}_{ei}=T_{e0i}-k_{e0i}{\bar{\varepsilon }}_{i}(i=1,2)$$ (13)

Where, $T_{e0}=-kn_{p}\omega_{0}/(k_{1}n_{p}{}^{2}\omega_{0}{}^{2}+1)$, $k_{e0}=n_{p}k(-k_{1}n_{p}{}^{2}\omega_{0}{}^{3}+\omega_{0})/{\left(k_{1}n_{p}{}^{2}\omega_{0}{}^{2}+1\right)}^{2}$, $k_{1}=n_{p}{}^{2}T_{r}{}^{2}$, $k=3n_{p}L_{m}{}^{2}U_{n}{}^{2}/R_{s}{}^{2}R_{r}$.

Introducing a small parameter *ε*_3_ for the phase error, then taking Eqs (12)~(13) into Eq (11), expanding Eq (11) with Taylor’s method at *α* = *α*_0_+*ε*_3_. We can get the Eq (14) as follows:

$$A_{0}\dot{\bar{\varepsilon }}=B_{0}\bar{\varepsilon }+v$$ (14)

with

$$A_{0}=(\begin{array}{ccc} {a^{\prime }}_{11} & a_{120} & 0\\ a_{210} & {a^{\prime }}_{22} & 0\\ 0 & 0 & 1 \end{array}),\;B_{0}=(\begin{array}{ccc} {b^{\prime }}_{11} & -b_{120} & -b_{130}\\ -b_{210} & {b^{\prime }}_{22} & -b_{230}\\ \omega_{0}/2 & -\omega_{0}/2 & 0 \end{array}),\;v={\left(\begin{array}{ccc} v_{1} & v_{2} & 0 \end{array}\right)}^{T},\;\bar{\varepsilon }={\left(\begin{array}{ccc} {\bar{\varepsilon }}_{1} & {\bar{\varepsilon }}_{2} & {\bar{\varepsilon }}_{3} \end{array}\right)}^{T}$$

$$a_{11}^{\prime }=1+a_{110},\;a_{22}^{\prime }=\eta +a_{220},\;v={\left(\begin{array}{ccc} v_{1} & v_{2} & 0 \end{array}\right)}^{T},\;\dot{\bar{\varepsilon }}={\left(\begin{array}{ccc} {\dot{\bar{\varepsilon }}}_{1} & {\dot{\bar{\varepsilon }}}_{2} & {\dot{\bar{\varepsilon }}}_{3} \end{array}\right)}^{T},\;\bar{\varepsilon }={\left(\begin{array}{ccc} {\bar{\varepsilon }}_{1} & {\bar{\varepsilon }}_{2} & {\bar{\varepsilon }}_{3} \end{array}\right)}^{T}$$

$$b_{11}^{\prime }=-[f_{1}/(m_{1}r^{2})+k_{e01}/(m_{1}r^{2}\omega_{0})+b_{110}],\;b_{22}^{\prime }=-[f_{2}/(m_{1}r^{2})+k_{e02}/(m_{1}r^{2}\omega_{0})+b_{220}]$$

$$b_{130}=-\eta \omega_{0}[r_{m}\cos(-2\alpha_{0}+\gamma_{x})/\mu_{x}+r_{m}\cos(-2\alpha_{0}+\gamma_{y})/\mu_{y}+r_{m}r_{l}{}^{2}\cos(-2\alpha_{0}+\theta_{1}-\theta_{2}+\gamma_{\psi })/\mu_{\psi }]$$

$$b_{230}=\eta \omega_{0}[r_{m}\cos(2\alpha_{0}+\gamma_{x})/\mu_{x}+r_{m}\cos(2\alpha_{0}+\gamma_{y})/\mu_{y}+r_{m}r_{l}{}^{2}\cos(2\alpha_{0}-\theta_{1}+\theta_{2}+\gamma_{\psi })/\mu_{\psi }]$$

$$v_{1}=T_{e01}/(m_{1}r^{2}\omega_{0})-f_{1}/(m_{1}r^{2})-\kappa_{10},\;v_{2}=T_{e02}/(m_{1}r^{2}\omega_{0})-f_{2}/(m_{1}r^{2})-\kappa_{20}$$

When two ERs achieve synchronous motion, the perturbation parameters $\varepsilon_{1}=\varepsilon_{2}=\varepsilon_{3}=0$, ${\dot{\varepsilon }}_{1}={\dot{\varepsilon }}_{2}={\dot{\varepsilon }}_{3}=0$. Therefore, Eq (14) can be tidied up to obtain the synchronization criterion of the two ERs. The synchronization criterion are shown in Eq (15).

$$\begin{array}{l} T_{e01}=f_{1}\omega_{0}+m_{1}r^{2}\omega_{0}\kappa_{10}\\ T_{e02}=f_{2}\omega_{0}+m_{1}r^{2}\omega_{0}\kappa_{20} \end{array}$$ (15)

Where, |*T*_*e*01_|≤*T*_*eN*1_, |*T*_*e*02_|≤*T*_*eN*2_. *T*_*eN*1_ and *T*_*eN*2_ are the rated electromagnetic torques.

Since the vibration system achieves synchronization criterion, ***v*** = 0. Taking ***v*** = 0 into Eq (14), we can get Eq (16).


$$A_{0}\dot{\bar{\varepsilon }}=B_{0}\bar{\varepsilon }$$ (16)

Because |***A***_0_|≠0, so Eq (14) can be rewritten as Eq (17)

$$\dot{\bar{\varepsilon }}=(A_{0}{}^{-1}B_{0})\bar{\varepsilon }$$ (17)

By $|\lambda I-A_{0}{}^{-1}B_{0}|=0$, we can obtain the characteristic equation of Eq (18) as follows:

$$\lambda^{3}+d_{1}\lambda^{2}+d_{2}\lambda +d_{3}=0$$ (18)

Where, *d*_1_ = *D*_1_/*D*_0_, *d*_2_ = *D*_2_/*D*_0_, *d*_3_ = *D*_3_/*D*_0_, $D_{3}=(b_{130}b_{210}+b_{11}^{\prime }b_{230}-b_{120}b_{230}-b_{22}^{\prime }b_{130})\omega_{0}/2$, $D_{2}=(a_{210}b_{130}+{a^{\prime }}_{22}b_{230}-a_{120}b_{230}-{a^{\prime }}_{11}b_{230})\omega_{0}/2+b_{11}^{\prime }b_{22}^{\prime }-b_{120}b_{210}$, $D_{1}=-a_{210}b_{120}-{a^{\prime }}_{11}b_{22}^{\prime }-{a^{\prime }}_{22}b_{11}^{\prime }-a_{120}b_{210}$, $D_{0}=a_{11}^{\prime }a_{22}^{\prime }-a_{120}a_{210}$.

When Eq (18) satisfies the Hurwitz condition, the synchronous state of the vibration system is stable. So the stability conditions for synchronous motion are as follows:

$$\left\{ \begin{array}{l} d_{2}>0\\ d_{3}>0\\ d_{1}d_{2}>d_{3} \end{array}\right.$$ (19)

## 4. Design of controllers

**Fig 2:**
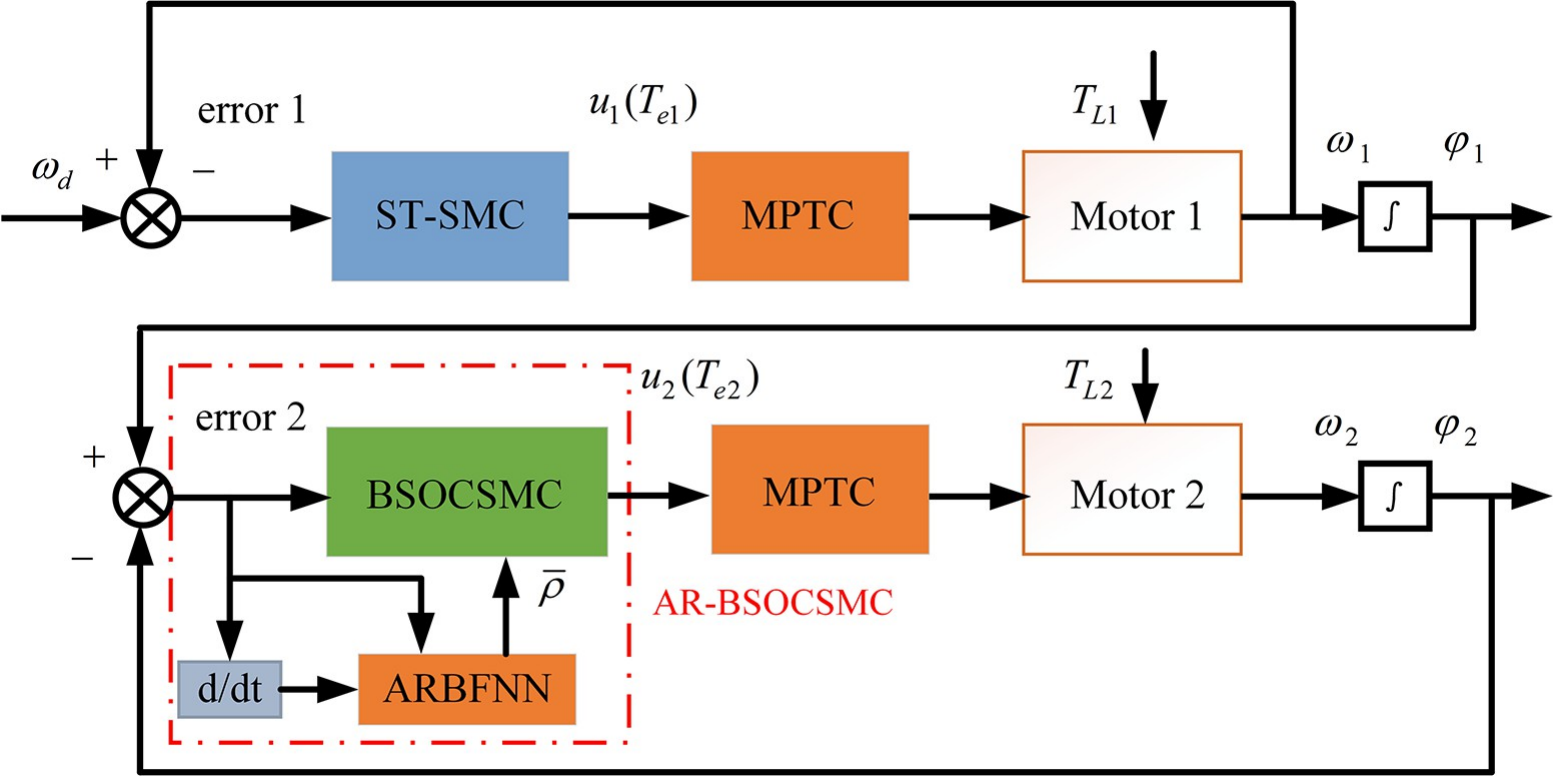
Structure of the control system.

**Fig 3:**
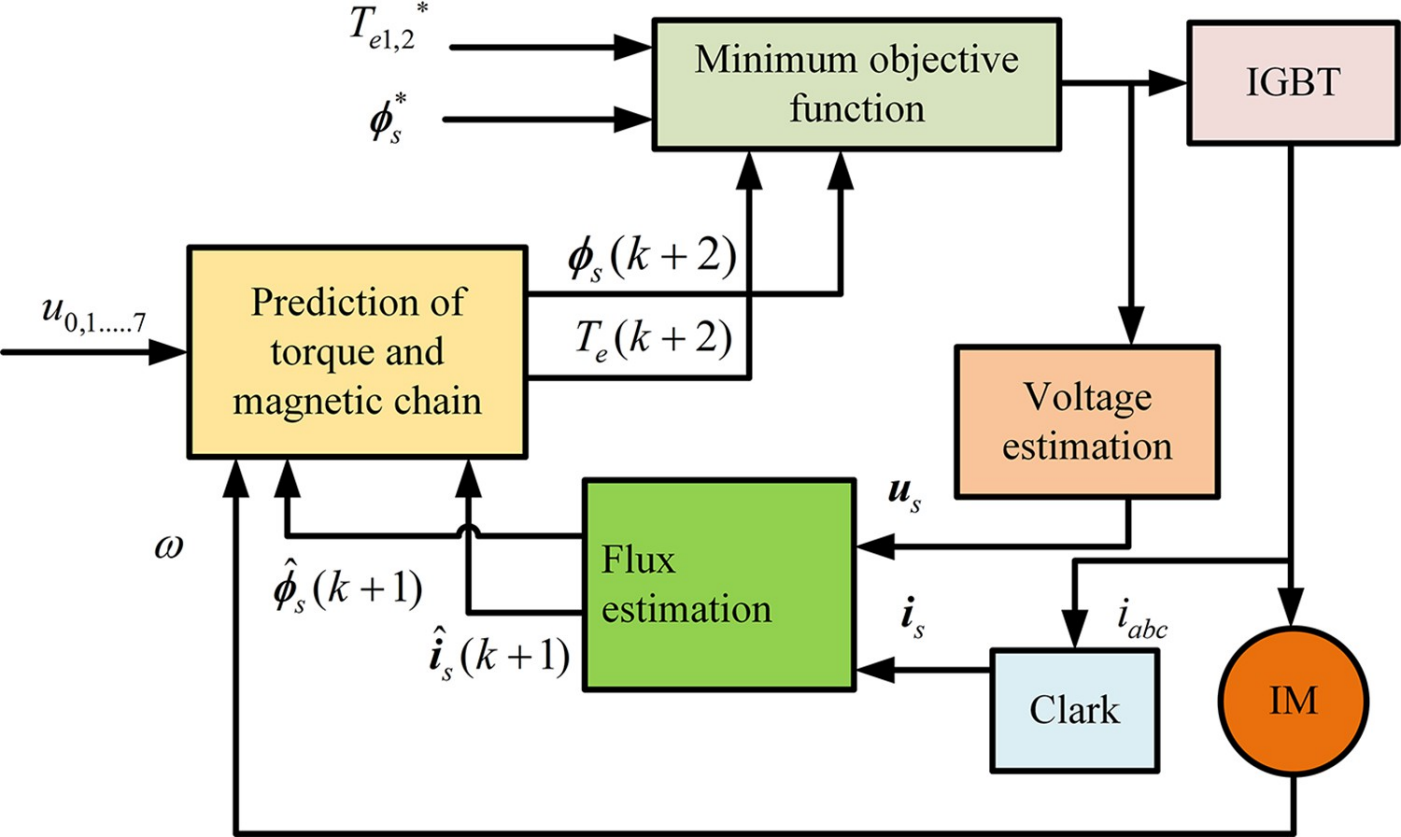
Model predictive torque control (MPTC).

The master-slave control strategy is selected to track and control the two motors, then the structure of the control system is shown in Fig 2. Motor 1 as the master motor, motor 2 as the slave motor. Motor 2 follows the motion state of motor 1. We set *ω*_*d*_ as the target speed, and *ω*_1_ is the actual speed of motor 1. The input of the control system is error 1, which generates the controlled object *u*_1_ by the ST-SMC controller. *u*_1_ (torque *T*_*e*1_) is used as an input to MPTC to control motor 1. Error 2 is generated by phase *φ*_1_ of ER1 and phase *φ*_2_ of ER2, similarly, error 2 generates controlled object *u*_2_ (torque *T*_*e*2_) by means of the BSOCSMC controller based on ARBFNN estimation (AR-BSOCSMC). The structure of the MPTC is shown in Fig 3.

### 4.1 Controller of the master motor

The equation of motion for motor 1 is represented by Eq (20).


$${\dot{\omega }}_{1}=1/J_{1}(T_{e1}-f_{1}\omega_{1}-T_{L1})$$ (20)

We choose the sliding mode surface as:

$$s=\omega_{d}-\omega_{1}$$ (21)

According to the theory of Super-Twisting sliding mode control, we design the mathematical form of the controlled object *u*_1_ as Eq (22) in order to reach the sliding-mode surface quickly.

$$u_{1}=T_{e1}=\lambda_{0}{\left|s\right|}^{1/2}\mathrm{sgn}(s)+\int_{0}^{t}\beta_{0}\mathrm{sgn}(s)dt$$ (22)

Where, both *λ*_0_ and *β*_0_ represent gain, and *λ*_0_>0, *β*_0_>0.

By deriving Eq (21) once, we can get the following equation.


$$\dot{s}=-\lambda_{1}{\left|s\right|}^{1/2}\mathrm{sgn}(s)-\int_{0}^{t}\beta_{1}\mathrm{sgn}(s)dt+\upsilon$$ (23)

Where, *λ*_1_ = *λ*_0_/*J*_1_, *β*_1_ = *β*_0_/*J*_1_, *υ* = (*f*_1_*ω*_1_−*T*_*L*1_)/*J*_1_.

Selecting ***ξ*** as the status variable, so ***ξ*** is as follows:

$$\xi =[\begin{array}{c} \xi_{1}\\ \xi_{2} \end{array}]=[\begin{array}{c} {\left|s\right|}^{1/2}\mathrm{sgn}(s)\\ \int_{0}^{t}\beta_{1}\mathrm{sgn}(s)dt \end{array}]$$ (24)

According to Eq (24), derivative $\dot{\xi }$ of the state variable ***ξ*** can be expressed as:

$$\dot{\xi }=-{\left|s\right|}^{-1/2}(A\xi -\rho )$$ (25)

with

$$A=[\begin{array}{cc} \lambda_{1}/2 & 1/2\\ -\beta_{1} & 0 \end{array}],\;\rho =[\begin{array}{c} \upsilon /2\\ 0 \end{array}]$$

It is necessary to prove the stability of the ST-SMC controller, so the Lyapunov function is designed as:

$$V=\xi^{T}p\xi$$ (26)

Because *V*>0, thus ***p*** needs to be a positive-definite matrix.


$$p=[\begin{array}{cc} \lambda_{1}{}^{2}/4+2\beta_{1} & \lambda_{1}/4\\ \lambda_{1}/4 & 1 \end{array}]$$ (27)

By deriving Eq (26) once, we can get the following equation.

$$\dot{V}=-{\left|s\right|}^{-1/2}\xi^{T}(A^{T}p+pA)\xi +{\left|s\right|}^{-1/2}\rho^{T}p\xi +{\left|s\right|}^{-1/2}\xi^{T}p\rho$$ (28)

Selecting *δ*>0 and satisfied |*υ*/2|≤*δ*|*ξ*_1_|, so Eq (28) can be rewritten as:

$$\begin{array}{l} \dot{V}\leq -{\left|s\right|}^{-1/2}\xi^{T}(A^{T}p+pA)\xi +{\left|s\right|}^{-1/2}\xi^{T}\left[\begin{array}{cc} \delta & 0\\ 0 & 0 \end{array}\right]p\xi +{\left|s\right|}^{-1/2}\xi^{T}p\left[\begin{array}{cc} \delta & 0\\ 0 & 0 \end{array}\right]\xi \\ \;=-{\left|s\right|}^{-1/2}\xi^{T}N\xi \end{array}$$ (29)

with

$$\begin{array}{l} N=A^{T}p+pA-p\left[\begin{array}{cc} \delta & 0\\ 0 & 0 \end{array}\right]-\left[\begin{array}{cc} \delta & 0\\ 0 & 0 \end{array}\right]p\\ \;=\lambda_{1}/4\left[\begin{array}{cc} \lambda_{1}{}^{2}+6\beta_{1}-2\delta (\lambda_{1}+8\beta_{1}/\lambda_{1}) & \lambda_{1}-\delta \\ \lambda_{1}-\delta & 1 \end{array}\right] \end{array}$$

$\dot{V}<0$ can keep the control system stable, thus it is necessary to satisfy that the matrix ***N*** is a positive-definite matrix. Considering the definition of a positive-definite matrix, thus the condition that ***N*** is a positive-definite matrix is as follows:

$$\left\{ \begin{array}{l} \lambda_{1}{}^{2}+6\beta_{1}-2\delta (\lambda_{1}+8\beta_{1}/\lambda_{1})>0\\ \lambda_{1}>0\\ \beta_{1}>0 \end{array}\right.$$ (30)

According to Eq (30), *λ*_1_ and *β*_1_ need to satisfy the following relationship.


$$\left\{ \begin{array}{l} \lambda_{1}>8\delta /3\\ \beta_{1}>\lambda_{1}\delta^{2}/(6\lambda_{1}-16\delta ) \end{array}\right.$$ (31)

The above calculations and analysis show that when *λ*_1_ and *β*_1_ meet the required conditions, the controlled object *T*_*e*1_ is stable and the control system is asymptotically stable.

The motor may be affected by disturbances during operation, so Eq (20) can be rewritten as Eq (32) after the disturbance is applied.

$${\dot{\omega }}_{1}=1/J_{1}(T_{e1}-f_{1}\omega_{1}-T_{L1})+H$$ (32)

Where, *H* indicates perturbation, and *H* is bounded.

Similarly, Eq (23) can be rewritten as follows:

$$\dot{s}=-\lambda_{1}{\left|s\right|}^{1/2}\mathrm{sgn}(s)-\int_{0}^{t}\beta_{1}\mathrm{sgn}(s)dt+\upsilon -H$$ (33)

Selecting *δ*>0 and satisfied |(*υ*−*H*)/2|≤*δ*|*ξ*_1_|. Referring to the previous analysis, the control system is asymptotically stable even with disturbances.

### 4.2 Controller of the slave motor

The last term in Eq (5) can be written as:

$${\ddot{\varphi }}_{2}=T_{e2}/J_{2}-f_{2}{\dot{\varphi }}_{2}/J_{2}-G$$ (34)

Where, *G* stands for an uncertain term, *G* = *T*_*L*2_/*J*_2_.

Since ER2 tracks the phase of ER1, thus the tracking error is defined as:

$$e=\varphi_{1}-\varphi_{2}$$ (35)

Referring to the theory of backstepping control, we respectively define the stable function *z*_1_ and Lyapunov function *V*_1_ as:

$$z_{1}=ke$$ (36)

$$V_{1}=e^{2}/2$$ (37)

Where, *k*>0.

Then, let’s define the dummy quantity *z*_2_ as:

$$z_{2}={\dot{\varphi }}_{1}+ke$$ (38)

According to Eq (38), the error *e*_1_ of *z*_2_ can be calculated as:

$$e_{1}=z_{2}-{\dot{\varphi }}_{2}={\dot{\varphi }}_{1}+ke-{\dot{\varphi }}_{2}$$ (39)

Then Eq (37) is written as Eq (40) by deriving.


$${\dot{V}}_{1}=ee_{1}-ke^{2}$$ (40)

It is known from Eq (40) that if *e*_1_ = 0, then the backstepping system is stable.

Considering the effect of the integral term in generalized sliding mode surface, we design the complementary sliding surface which is orthogonal to the generalized sliding mode surface. This is more effective in reducing the tracking error.

We respectively design the generalized sliding mode surface *s*_*a*_ and complementary sliding mode surface *s*_*b*_ as:

$$\begin{array}{l} s_{a}=e_{1}+\chi_{2}\int_{0}^{t}e_{1}(\tau )d\tau \\ s_{b}=e_{1}-\chi_{2}\int_{0}^{t}e_{1}(\tau )d\tau \end{array}$$ (41)

Where, *χ*_2_ is the sliding mode constant.

Combining the two terms in Eq (41), we can obtain *s*_*c*_ and ${\dot{s}}_{c}$

$$\begin{array}{l} s_{c}=s_{a}+s_{b}=2e_{1}\\ {\dot{s}}_{c}=2[{\dot{z}}_{2}-(T_{e2}/J_{2}-f_{2}{\dot{\varphi }}_{2}/J_{2}-G)] \end{array}$$ (42)

We design the Lyapunov function *V*_2_ as:

$$V_{2}=\varepsilon_{0}{\left(s_{a}+s_{b}\right)}^{2}/2+{\left({\dot{s}}_{a}+{\dot{s}}_{b}\right)}^{2}/2+\rho_{0}|s_{a}+s_{b}|$$ (43)

Where, *ε*_0_>0, *ρ*_0_ indicates the maximum value of uncertain term *G*, *ρ*_0_≥|*G*|.

By deriving Eq (43) once, we can get the following equation.


$${\dot{V}}_{2}=({\dot{s}}_{a}+{\dot{s}}_{b})[\varepsilon_{0}(s_{a}+s_{b})+({\ddot{s}}_{a}+{\ddot{s}}_{b})+\rho_{0}\mathrm{sgn}(s_{a}+s_{b})]$$ (44)

Defining ${\ddot{s}}_{a}+{\ddot{s}}_{b}$ as:

$${\ddot{s}}_{a}+{\ddot{s}}_{b}=-\varepsilon_{0}(s_{a}+s_{b})-k_{1}({\dot{s}}_{a}+{\dot{s}}_{b})-\rho_{0}\mathrm{sgn}(s_{a}+s_{b})]$$ (45)

Where, *k*_1_>0.

The stability of the system is related to whether Eq (45) is satisfied. If Eq (45) is satisfied, thus ${\dot{V}}_{2}\leq 0$ and the system is stable.

Combining Eq (45) with Eq (44), we design the mathematical form of the controlled object *u*_2_ as Eq (46).

$$u_{2}=T_{e2}=J_{2}({\dot{z}}_{2}+f_{2}{\dot{\varphi }}_{2}/J_{2}-{\bar{\rho }}_{0})+2J_{2}\int_{0}^{t}\left[\varepsilon_{0}(s_{a}+s_{b})+k_{1}({\dot{s}}_{a}+{\dot{s}}_{b})+{\bar{\rho }}_{0}\mathrm{sgn}(s_{a}+s_{b})\right]d\tau$$ (46)

Where, ${\bar{\rho }}_{0}$ is estimated from *ρ*_0_.

To ensure that the solution of Eq (45) is asymptotically stable, thus *s*_*c*_^(*n*)^ = 0 in finite time. We can rewrite Eq (45) as:

$${\ddot{s}}_{a}+{\ddot{s}}_{b}=-\varepsilon_{0}(s_{a}+s_{b})-\rho_{0}\mathrm{sgn}(s_{a}+s_{b})]$$ (47)

When Eq (48) is satisfied, ${\ddot{s}}_{c}\equiv 0$.


$$s_{a}+s_{b}\equiv -\varepsilon_{0}{}^{-1}\rho_{0}\mathrm{sgn}(s_{a}+s_{b})$$ (48)

In summary we can know that *s*_*c*_ and ${\dot{s}}_{c}$ will become zero in finite time. According to Eq (42), the error *e*_1_ of *z*_2_ will also become zero in finite time. Eq (40) is rewritten as:

$${\dot{V}}_{1}=-ke^{2}\leq 0$$ (49)

Since ${\dot{V}}_{1}\leq 0$, thus *V*_1_(*e*,0)≥*V*_1_(*e*,*t*), *e* is bounded.

The integral of Eq (49) can be described as:

$$\underset{t\to \infty }{\lim}\int_{0}^{t}(ke^{2})d\tau \leq V_{1}(e,0)-V_{1}(e,\infty )=\infty$$ (50)

Referring to Barbalat Lemma [23] and Eq (50), we can obtain the Eq (51).


$$\underset{t\to \infty }{\lim}e(t)=0$$ (51)

Therefore, the control system is stable.

${\bar{\rho }}_{0}$ is estimated by ARBFNN. The structure of the neural network is chosen as 2-5-1, and the RBFNN algorithm is as follows [24, 25]:

$$\begin{array}{c} h_{j}=g({\left\|x-c_{ij}\right\|}^{2}/b_{j}{}^{2})\\ \;=\exp(-{\left\|x-c_{ij}\right\|}^{2}/b_{j}{}^{2})\\ \;f=W^{T}h(x)+\varepsilon_{a} \end{array}$$ (52)

Where, *h*_*j*_ is a Gaussian function. *g* denotes the Gaussian activation function. ***x*** is the input of the RBFNN. *i* is the number of inputs. *j* stands for implied layer node. *b*_*j*_ is the width of Gaussian function. ***h*** denotes the output of the Gaussian function, $h={\left[\begin{array}{cccc} h_{1} & h_{2} & \cdots & h_{j} \end{array}\right]}^{T}$. ***W*** is the weight of the RBFNN. *ε*_*a*_ indicates the estimation error.

The input of RBFNN is defined as $x={\left[\begin{array}{cc} e & \dot{e} \end{array}\right]}^{T}$, then the output can be obtained as:

$${\bar{\rho }}_{0}={\hat{W}}^{T}h(x)$$ (53)

In Eq (53), $\hat{W}$ is the estimation of ***W***. $\hat{W}$ is obtained by the following equation.

$$\dot{\hat{W}}=-\gamma E^{T}PBh(x)$$ (54)

Where, *γ*>0, $E={\left[\begin{array}{cc} e & \dot{e} \end{array}\right]}^{T}$, $B={\left[\begin{array}{cc} 0 & 1 \end{array}\right]}^{T}$. ***P*** denotes a positive definite matrix.

## 5. Characterization and simulation

The parameters of two motors and vibration system are shown in Tables 1 and 2.

**Table 1:** Parameters of motors.

Parameters	Motor 1	Motor 2
Power Rating *P*/kW	1	1
Number of polar pairs *n*_*p*_	3	3
Frequency rating *f*_0_/Hz	50	50
Rated speed *n*/(r/min)	950	950
Stator resistance *R*_*s*_/Ω	5.75	5.4
Rotor resistance *R*_*r*_/Ω	5.4	5.3
Stator inductor *L*_*s*_/H	0.170	0.179
Rotor inductor *L*_*r*_/H	0.170	0.179
Coefficient of mutual inductance *L*_*m*_/H	0.115	0.125
Given magnetic chain $\phi_{s}^{*}$/Wb	0.8	0.8
Friction coefficient *f*_1,2_/(N·s·m/rad)	0.005	0.005

**Table 2:** Parameters of the vibration system.

Parameters	Value
The quality of the shaking table and motors *m*/kg	242
The rotational inertia of the shaking table *J*_*P*_/(kg·m^2^)	43.5
The spring stiffness in *x* direction *k*_*x*_/(N/m)	129322
The spring stiffness in *y* direction *k*_*y*_/(N/m)	105334
The spring stiffness in *ψ* direction *k*_*ψ*_/(N·m/rad)	30715
The damping coefficients in *x* direction *f*_*x*_/(N·s/m)	615.5
The damping coefficients in *y* direction *f*_*y*_/(N·s/m)	618
The damping coefficients in *ψ* direction *f*_*ψ*_/(N·s·m/rad)	180.2
The distance between *o* and *o*_1_ *l*_1_/m	0.3
The distance between *o* and *o*_2_ *l*_2_/m	0.3
The position angles of ER1 *θ*_1_/ (°)	30
The position angles of ER2 *θ*_2_/ (°)	150
The quality of ERs *m*_1_/kg	4
Rotational radius of ERs *r*/m	0.05

### 5.1 Characterization of synchronization conditions and stability conditions

**Fig 4:**
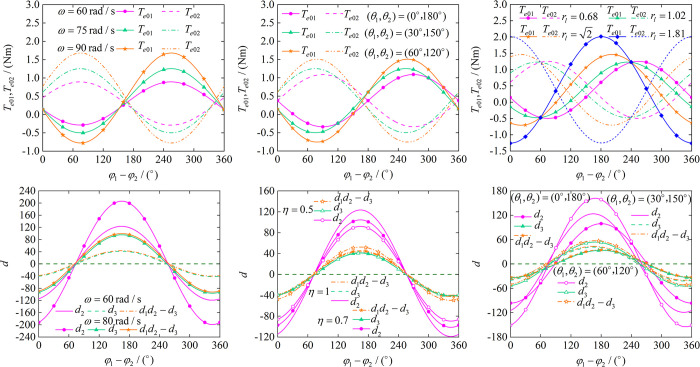
Characterization of synchronization conditions and stability conditions. (a) Synchronization conditions at different speeds. (b) Effect of *θ* on synchronization conditions. (c) Effect of *r*_*1*_ on synchronization conditions. (d) Stabilized areas at different speeds. (e) Effect of *η* on stabilized areas. (f) Effect of *θ* on stabilized areas.

The theory of synchronization and stability conditions has been derived in Eq (15) and Eq (19) in section 2, we continue to analyze its numerical aspects. In Fig 4, we can see that (a) represents the relationship between the phase error and the electromagnetic torque of the two motors. Although different phase errors result in different electromagnetic torque output from motors, but the electromagnetic torque is still less than the rated electromagnetic torque. The electromagnetic torque of motor 1 and motor 2 increase as the target speed increases. (b) indicates the effect of *θ*_1_ and *θ*_2_ on the electromagnetic torque with other parameters unchanged. We can see that the electromagnetic torques become larger after increasing the position angle, the phase errors corresponding to the highest point of the curve and the lowest point of the curve are different. In (c), the effect of the change in *r*_*l*_ on the electromagnetic torque is the same as in (a), the electromagnetic torque is also still less than the rated electromagnetic torque. The four curves show a pattern that their torques are equal at 60° and 240°. (d), (e) and (f) show the stability conditions of the synchronous motion. Because of *d*_2_>0, *d*_3_>0,*d*_1_*d*_2_>*d*_3_, so we can get the stable region of phase error when the speed is 60 rad/s and 80 rad/s by (d). From (d), the stable region is (75°~254°) and doesn’t change much at different speeds. When we change the parameter *η*, only *d*_2_ and *d*_1_*d*_2_−*d*_3_ are affected, and this phenomenon can be seen in (e). (f) shows the effect of changing the position angle on the stability conditions. As the position angle increases, the curves shift to the left overall, which indicates that the stable region has changed.

**Fig 5:**
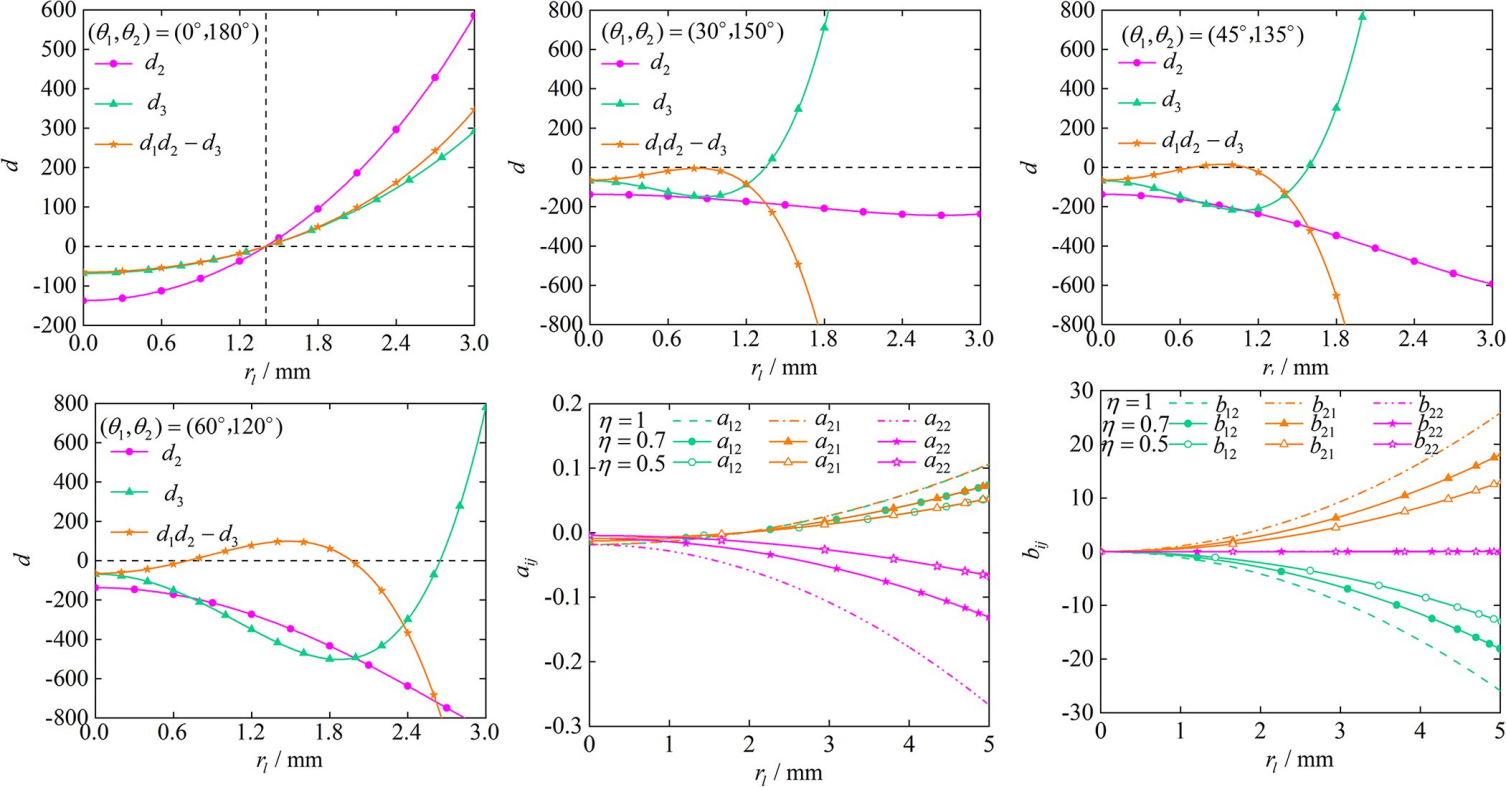
Effect of *r*_*l*_ on parameters *a*_*ij*_(*i*,*j* = 1,2), *b*_*ij*_(*i*,*j* = 1,2) and stability conditions for zero phase error. (a) Stability conditions at (*θ*_1_,*θ*_2_) = (0°,180°). (b) Stability conditions at (*θ*_1_,*θ*_2_) = (30°,150°). (c) Stability conditions at (*θ*_1_,*θ*_2_) = (45°,135°). (d) Stability conditions at (*θ*_1_,*θ*_2_) = (60°,120°). (e) Effect of *r*_*l*_ on parameters *a*_*ij*_(*i*,*j* = 1,2). (f) Effect of *r*_*l*_ on parameters *b*_*ij*_(*i*,*j* = 1,2).

The purpose of controlled synchronization is to achieve *φ*_1_−*φ*_2_ = 0, therefore, we analyze the relationship between *r*_*1*_ and *d* on the basis of *φ*_1_−*φ*_2_ = 0. First we set the position angle to 0° and 180°, it is obvious from Fig 5(A) that *d*_2_>0, *d*_3_>0 and *d*_1_*d*_2_−*d*_3_>0 are only satisfied when $r_{l}>\sqrt{2}$. After the position angle is increased, we can see from (b), (c)and (d) that *d*_2_>0, *d*_3_>0 and *d*_1_*d*_2_−*d*_3_>0 cannot be satisfied simultaneously despite increasing *r*_*l*_. Therefore, the self-synchronous motion to achieve *φ*_1_−*φ*_2_ = 0 requires the position angle to be 0 and $r_{l}>\sqrt{2}$ to be satisfied. (e), (f) denote the effect of *η* on *a*_*ij*_(*i*,*j* = 1,2) and *b*_*ij*_(*i*,*j* = 1,2), we can know that the stable capacity of the vibrating system is strongest at *η* = 1.

### 5.2 Self-synchronous simulation

**Fig 6:**
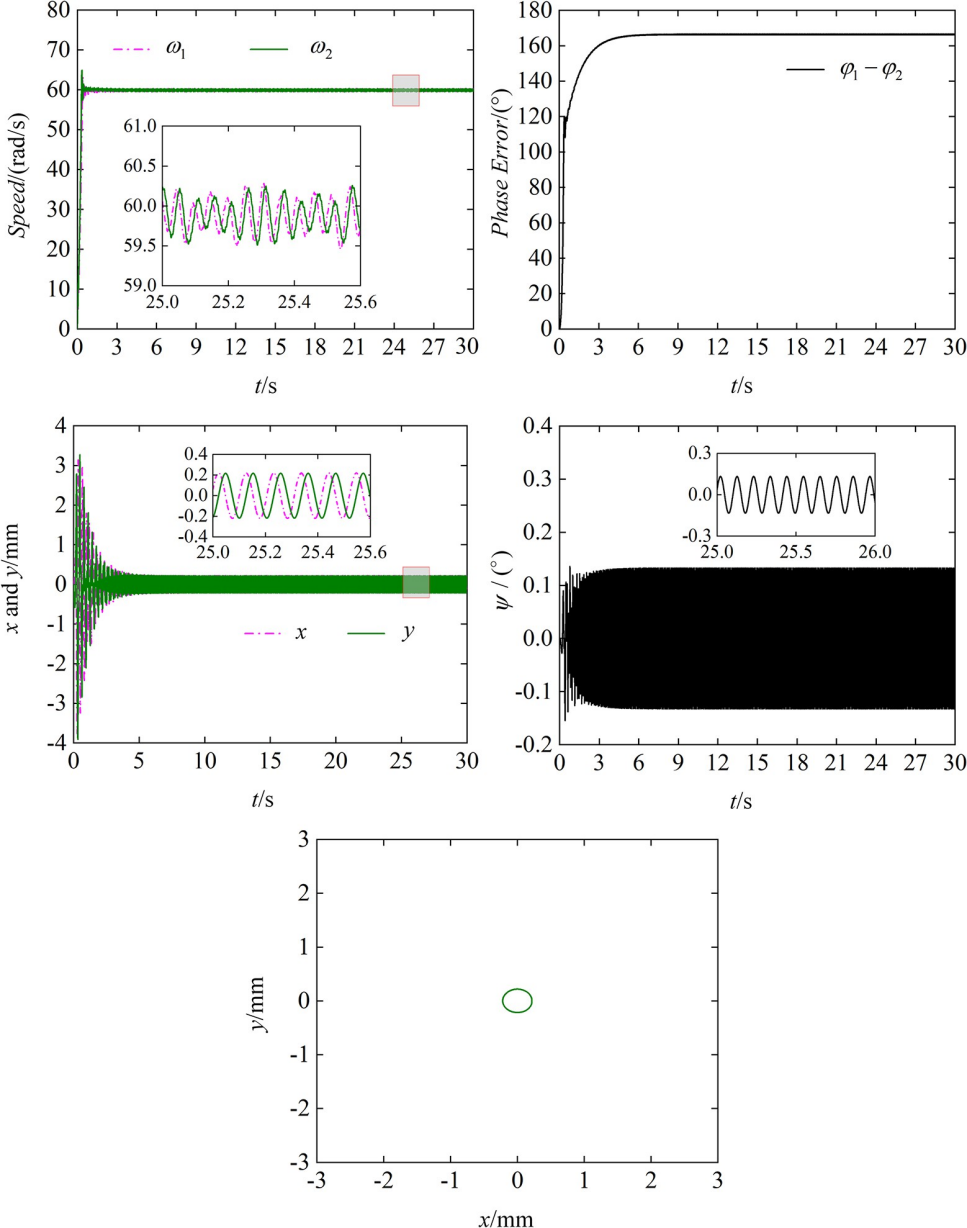
Results of self-synchronous simulation. (a) Speed of two motors. (b) Phase tracking error. (c) Response in *x*, *y* directions. (d) Response in *ψ* direction. (e) The trajectory of the body.

The limitations of the self-synchronization have been described in the previous section, so this section simulates the self-synchronization to further illustrate the need for the control method. The results of simulation are shown in Fig 6.

(a) reflects the speed of motor 1 and motor 2. We can see that at the beginning of the simulation, both motor 1 and motor 2 can quickly reach the target speed of 60 rad/s and stabilize around 60 rad/s. (b) is the phase error between the ERs. From (b), we can obtain that the phase error stabilizes around 165° after 10 s and does not achieve *φ*_1_−*φ*_2_ = 0, This phenomenon shows that the two motors are synchronized only in speed, not in phase error to zero. (c) and (d) are the responses of the vibrating system in three directions. The displacement response in *x*, *y* directions are stable with time between -0.2 mm and 0.2 mm, the *ψ* direction shows a small oscillation. As shown in (e), the trajectory of the shaking table at the steady state of 15~20 s is a small ellipse, which indicates that the amplitude of the body is stable but the amplitude is small. Simulation shows that the vibration system realizes the self-synchronous motion with equal speed but non-zero phase error. In practice, the vibrating screen driven by two motors will appear the phenomenon that the body amplitude is too small, which will lead to poor screening effect, and is not conducive to screening and conveying materials.

### 5.3 Controlled synchronous simulation

**Fig 7:**
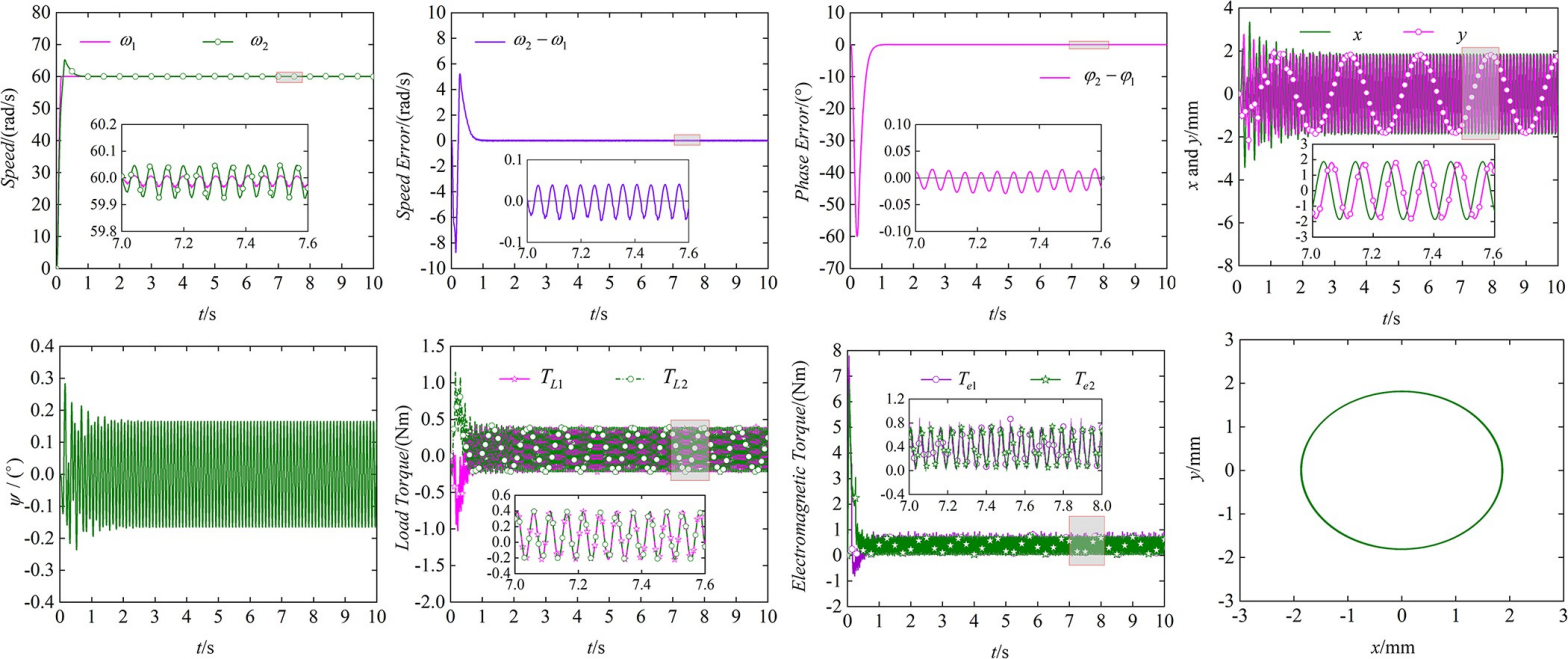
Results of controlled synchronization simulation. (a) Speed of two motors. (b) Speed tracking error. (c) Phase tracking error. (d) Response in *x*, *y* directions. (e) Response in *ψ* direction. (f) Load of motor 1 and motor 2. (g) Electromagnetic torque of two motors. (h) The trajectory of the body.

The controllers for the two motors have been designed in section 4, then we use simulation to verify the effectiveness of the control method. The results of simulation for controlled synchronization are shown in Fig 7.

(a) shows the speed curves of the two motors based on AR-BSOCSMC and MPTC. From (a), we can see that both motors reach the target speed of 60 rad/s, while the speed fluctuation range of motor 2 is ±0.06 rad/s, which is much smaller than the self-synchronous fluctuation. (b) indicates the speed error curves of motor 1 and motor 2. The maximum value of the speed error is 5 rad/s, which indicates that the maximum speed overshoot of motor 2 is 5 rad/s and the speed tracking error of the two motors is less than 0.05. In (c), the maximum value of phase error between ER1and ER2 is 60°, then motor 2 quickly tracks the phase of motor 1 and achieves the state of zero phase error at 1 s. The phenomenon indicates that the ERs have achieved synchronous motion with double synchronization of speed and phase. (d) and (e) are the responses of the vibrating system in three directions, and their response values are affected by phase synchronization. When the system reaches a synchronized state with zero phase error, the vibration displacement is stable between -2 mm and 2 mm in *x* and *y* directions, thus the motion trajectory of the shaking table is elliptical as shown in (h). Compared (h) with Fig 6(E), it can be known that the state of zero phase error can make the amplitude of the shaking table increase greatly, and the application to the vibrating screen can significantly improve the efficiency and process effect. (f) and (g) respectively indicate the load torque and electromagnetic torque of the two motors, the relationship between load torque and electromagnetic torque meets the requirements for achieving synchronous motion. The value of the electromagnetic torque when the phase error is zero is consistent with the analysis in Fig 4(A). Simulation results can show that the control methods and control strategy designed in this paper can realize the synchronous motion with equal speed and zero phase error. Applying them to the vibrating screen can increase the amplitude and improve the screening efficiency to meet higher process requirements.

### 5.4 Comparison of different methods and robustness analysis

**Fig 8:**
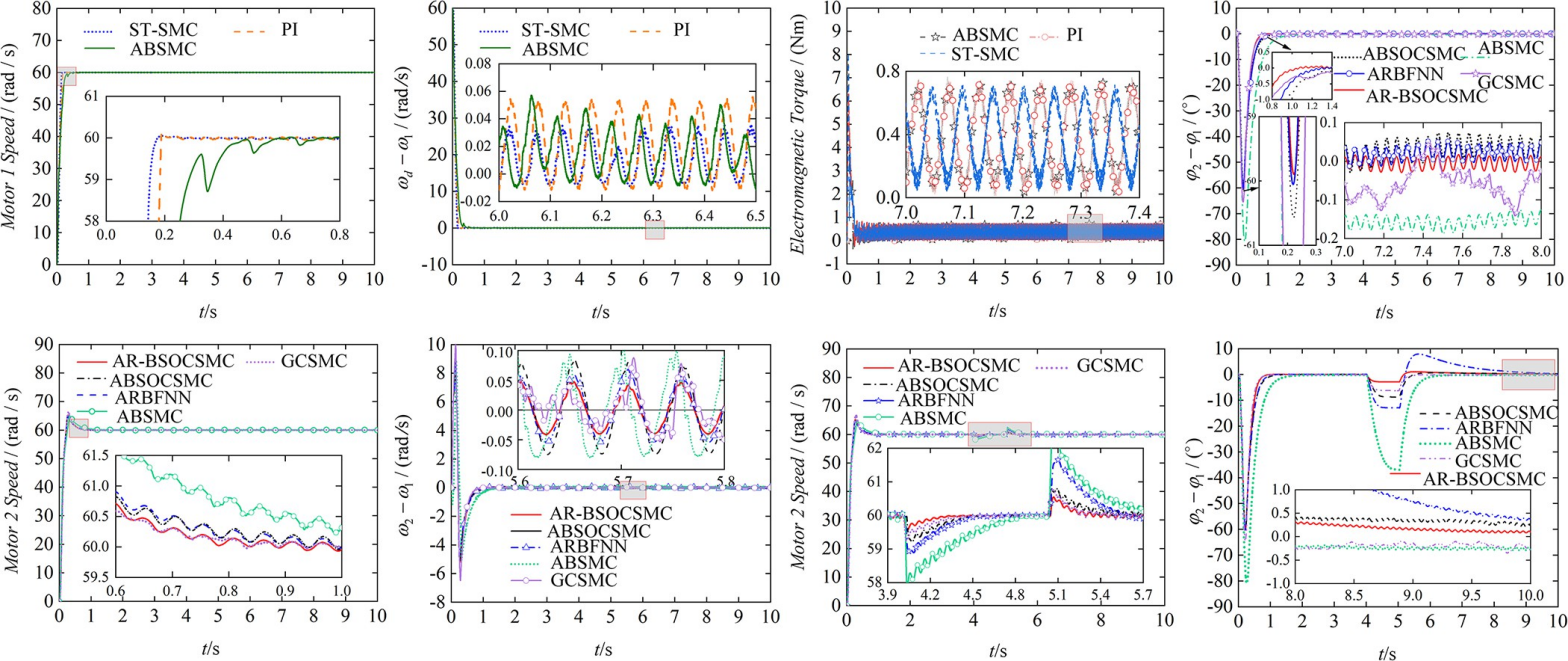
Comparison and analysis of multiple control methods. (a) Speed of motor 1. (b) Speed tracking error. (c) Electromagnetic torque of motor 1of motor 1. (d) Phase tracking error. (e) Speed of motor 2. (f) Speed tracking error of motor 2. (g) Speed of motor 2 under perturbation. (h) Phase tracking error under perturbation.

The control methods designed in this paper have been verified in terms of effectiveness, so we continue to analyze the advanced and robustness of controllers by means of methods comparison. The results of simulation are shown in Fig 8.

For motor 1, we compared the ST-SMC controller with a conventional PI controller and an adaptive backstepping sliding mode controller (ABSMC) in simulation. From (a), we can see that the three control methods show the same effect on the whole. However, the ST-SMC is more responsive during the start-up phase of the motor, and the speed tracking error represented in (b) is similarly minimized. In (c), the ST-SMC minimizes fluctuations in the electromagnetic torque of motor 1 compared to other controllers. The above phenomena can show that the controller designed for motor 1 in the paper is significantly advanced. (d) shows the phase tracking curve for ERs, AR-BSOCSMC shows good tracking performance compared to adaptive BSOCSMC (ABSOCSMC), global CSMC (GCSMC), and so on. With AR-BSOCSMC, the maximum value of phase error of the two motors is 60°, which is the smallest among the comparative control methods. In addition, AR-BSOCSMC has the smallest phase tracking error. The speed of motor 2 is able to track the speed of motor 1 well under the control of different controllers, but the AR-BSOCSMC brings the best results in terms of tracking error and weakening of chattering, which can be obtained from (e), (f). At the simulation time of 4 s, we disconnect the controller, forcing the vibration system to self-synchronize, and resume the controlled synchronization at 5 s. This time period can be regarded as the vibrating system is subjected to external disturbance. From (g), the speed fluctuation with AR-BSOCSMC after applying the perturbation is the smallest and the regulation time is shorter than others. In (h), The fluctuation in phase error for AR-BSOCSMC is about 3°, while other control methods are much larger than 3°. In addition, when the vibration system resumes controlled synchronization, the phase overshoot with AR-BSOCSMC is very small and the phase error returns to zero in a short time. By analyzing (g), (h), it is known that AR-BSOCSMC has strong robustness. Above analysis leads to the conclusion that the control method designed in this paper is significantly advanced and robust.

## 6. Conclusions

This paper investigates that the controlled synchronization method can be applied to realize the miniaturization of the vibrating screens. And the results indicate that the control strategy and control method proposed in this paper are effective, and have higher control accuracy and better robustness compared with other control methods. According to the dynamics model and based on the small parameter method, we obtained the synchronization conditions and the stability conditions of the vibrating screen driven by two motors. In order to verify the correctness of the theoretical derivation, the synchronization and stability conditions were numerically analyzed and visualized, and it is concluded that the condition of self-synchronization to achieve zero phase error is $r_{l}>\sqrt{2}$. We respectively designed ST-SMC controller and AR-BSOCSMC controller for motor 1 and motor 2, and analyzed the Lyapunov stability. The simulation indicates that the controlled synchronization can make the phase error become zero. The total excitation force of the vibration system is the sum of the excitation force generated by the ER1 and the excitation force generated by the ER2. In this case, the displacement of the shaker in the *x* and *y* directions increase significantly, and its motion trajectory is elliptical. The screening efficiency of the vibrating screen reaches a more ideal state. In addition, we demonstrate that the controllers designed in this paper are better in terms of robustness, weakening of chattering, and control accuracy by comparative simulation with other controllers.

## Supporting information

S1 Fig Mechanical model of a vibrating screen driven by two motors. (ZIP)

S2 Fig Structure of the control system. (ZIP)

S3 Fig Model predictive torque control (MPTC). (ZIP)

S4 Fig Characterization of synchronization conditions and stability conditions. (ZIP)

S5 Fig Effect of *r*_*l*_ on parameters *a*_*ij*_(*i*,*j* = 1,2), *b*_*ij*_(*i*,*j* = 1,2) and stability conditions for zero phase error. (ZIP)

S6 Fig Results of self-synchronous simulation. (ZIP)

S7 Fig Results of controlled synchronization simulation. (ZIP)

S8 Fig Comparison and analysis of multiple control methods. (ZIP)

S1 Table Parameters of motors. (DOCX)

S2 Table Parameters of the vibration system. (DOCX)

## References

[1] Zhang XL, Wen BC, Zhao CY. Synchronization of three homodromy coupled exciters in a non-resonant vibrating system of plane motion[J]. Acta Mechanica Sinica, 2012, 28(5): 1424–1435.

[2] Wen BC, Fan J, Zhao CY, Xiong WL. Vibratory synchronization and controlled synchronization in engineering[M]. Beijing: Science Press, 2009.

[3] Blekhman II. Synchronization in science and technology[M], New York: ASME Press,1988.

[4] Blekhman II. Synchronization of dynamical systems[M], Moscow: Nauka,1971.

[5] Wen BC, Zhang H, Liu SY, He Q, Zhao CY. Theory and techniques of vibrating machinery and their applications[M], Beijing: Science Press, 2010.

[6] Zhao CY, Zhu HT, Zhang YM, Wen BC. Synchronization of two coupled exciters in a vibrating system of spatial motion[J], Acta Mechanica Sinica, 2010, 26 (3): 477–493.

[7] Zhao CY, Zhu HT, Wang RZ, Wen BC. Synchronization of two non-identical coupled exciters in a non-resonant vibrating system of linear motion. Part I: Theoretical analysis[J]. Shock and Vibration, 2009, 16(5): 505–515. doi: 10.3233/SAV-2009-0485

[8] Zhao CY, Zhu TT, Bai TJ, Wen BC. Synchronization of two non-identical coupled exciters in a non-resonant vibrating system of linear motion. Part II: Numeric analysis[J]. Shock and Vibration, 2009, 16(5): 517–528.

[9] Zhao CY, Zhao QH, Gong ZM, Wen BC. Synchronization of two self-synchronous vibrating machines on an isolation frame[J], Shock and Vibration, 2011, 18 (1–2) 73–90.

[10] Jia L, Liu ZL. Multifrequency composite synchronization of three inductor motors with the method of fixed speed ratio in a vibration system[J]. Proceedings of the Institution of Mechanical Engineers, Part E: Journal of Process Mechanical Engineering, 2023, 237(2): 254–268.

[11] Jia L, Wang C, Liu ZL. Multifrequency controlled synchronization of four inductor motors by the fixed frequency ratio method in a vibration system[J]. Scientific Reports, 2023, 13(1): 2467. 36774384 10.1038/s41598-023-29603-y PMC9922278

[12] Kong XX, Zhou C, Wen BC. Composite synchronization of four exciters driven by induction motors in a vibration system[J]. Meccanica, 2020, 55: 2107–2133.

[13] Kong XX, Chen CZ, Wen BC. Composite synchronization of three eccentric rotors driven by induction motors in a vibrating system[J]. Mechanical Systems & Signal Processing, 2018, 102(MAR.1):158–179. doi: 10.1016/j.ymssp.2017.09.025

[14] Fang P, Shi S, Zou M, Lu XG, Wang DJ. Self-synchronization and control-synchronization of dual-rotor space vibration system[J]. International Journal of Non-Linear Mechanics, 2022, 139: 103869.

[15] Zhang LP, Feng J, Qiu WM, Zhang LL. Experiment and Simulation Research on Synchronization Control of Shaking Tables System Based on Adaptive Sliding Mode Controller[J]. Journal of Vibration Engineering & Technologies, 2022: 1–23.

[16] Huang ZL, Li YM, Song GQ, Zhang XL, Zhang ZC. Speed and phase adjacent cross-coupling synchronous control of multi-exciters in vibration system considering material influence[J]. IEEE Access, 2019, 7: 63204–63216.

[17] Huang ZL, Song GQ, Li YM, Sun MN. Synchronous control of two counter-rotating eccentric rotors in nonlinear coupling vibration system[J]. Mechanical Systems and Signal Processing, 2019, 114: 68–83.

[18] Huang ZL, Song GQ, Zhang ZC, Zhang XL. Control synchronization of two nonidentical homodromy exciters in nonlinear coupled vibration system[J]. IEEE Access, 2019, 7: 109934–109944.

[19] Fang P, Wang Y, Zou M, Zhang ZL. Combined control strategy for synchronization control in multi-motor-pendulum vibration system[J]. Journal of Vibration and Control, 2022, 28(17–18): 2254–2267.

[20] Xi XJ, Mobayen S, Ren H, Jafari S. Robust finite-time synchronization of a class of chaotic systems via adaptive global sliding mode control[J]. Journal of Vibration and Control, 2018, 24(17): 3842–3854.

[21] Zhai AB, Wang J, Zhang HY, Lu GD, Li H. Adaptive robust synchronized control for cooperative robotic manipulators with uncertain base coordinate system[J]. ISA transactions, 2022, 126: 134–143. 34344538 10.1016/j.isatra.2021.07.036

[22] Shi SQ, Fang P, Zou M, Peng H. Zhang ZL.et al. Control synchronization of three eccentric rotors driven by motors in space considering adaptive fuzzy sliding mode control algorithm[J]. Journal of Vibration and Control, 2023, 29(1–2): 375–386.

[23] El-Sousy FFM. Adaptive dynamic sliding-mode control system using recurrent RBFN for high-performance induction motor servo drive[J]. IEEE Transactions on Industrial Informatics, 2013, 9(4): 1922–1936.

[24] Jiang YM, Wang YN, Miao ZQ, et al. Composite-learning-based adaptive neural control for dual-arm robots with relative motion[J]. IEEE Transactions on Neural Networks and Learning Systems, 2020, 33(3): 1010–1021. 10.1109/TNNLS.2020.3037795 33361000

[25] Guo Q, Chen ZL. Neural adaptive control of single-rod electrohydraulic system with lumped uncertainty[J]. Mechanical Systems and Signal Processing, 2021, 146: 106869.

