# Supplementary figures and images for: Controlled synchronization of a vibrating screen driven by two motors based on improved sliding mode controlling method
Source: PLoS One. 2023 Nov 21;18(11):e0294726. doi: 10.1371/journal.pone.0294726 (PMC10662758; doi:10.1371/journal.pone.0294726)

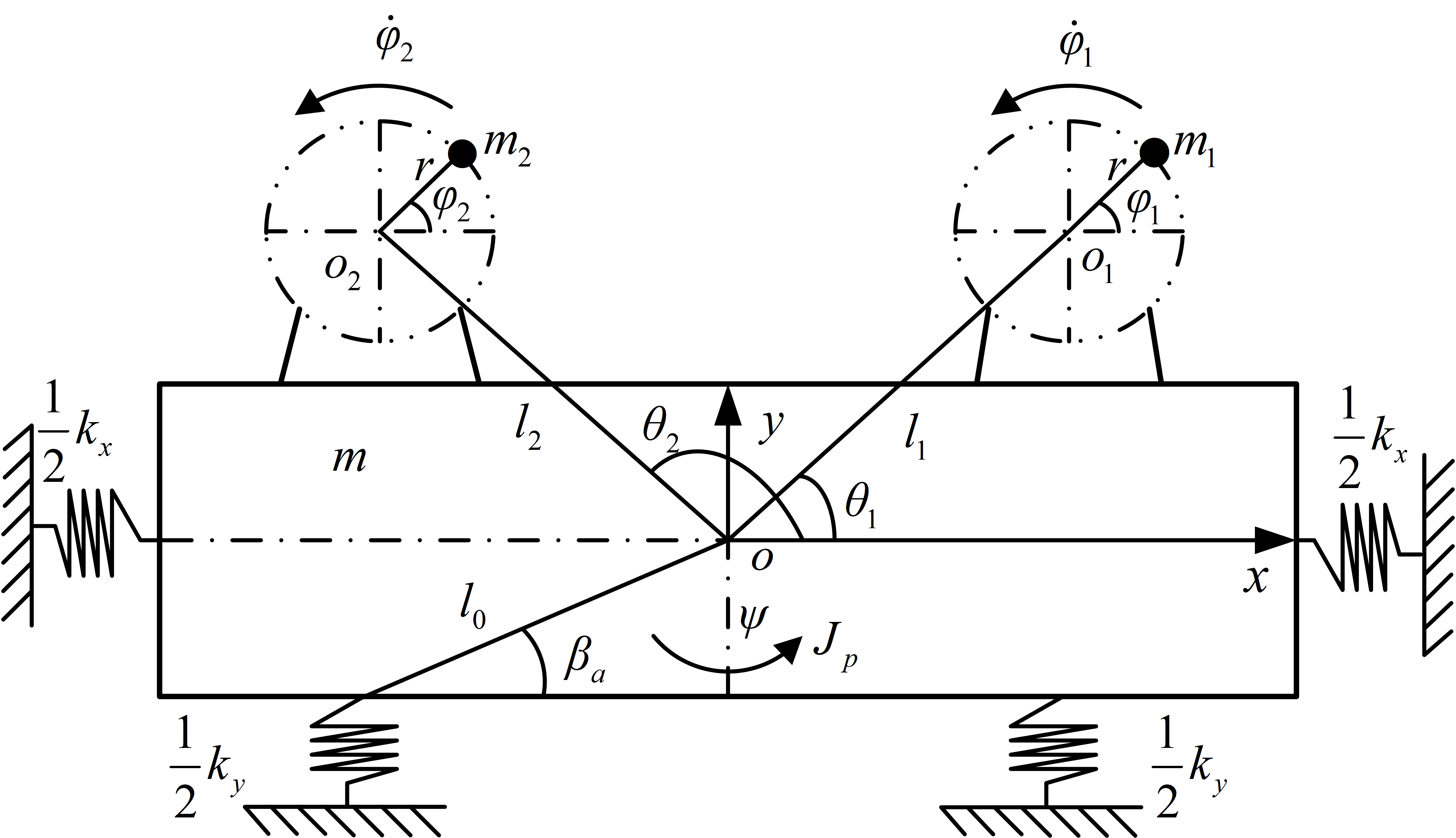

Supplement: S1 Fig — (ZIP) [file pone.0294726.s001.zip › S1 Fig.tif]

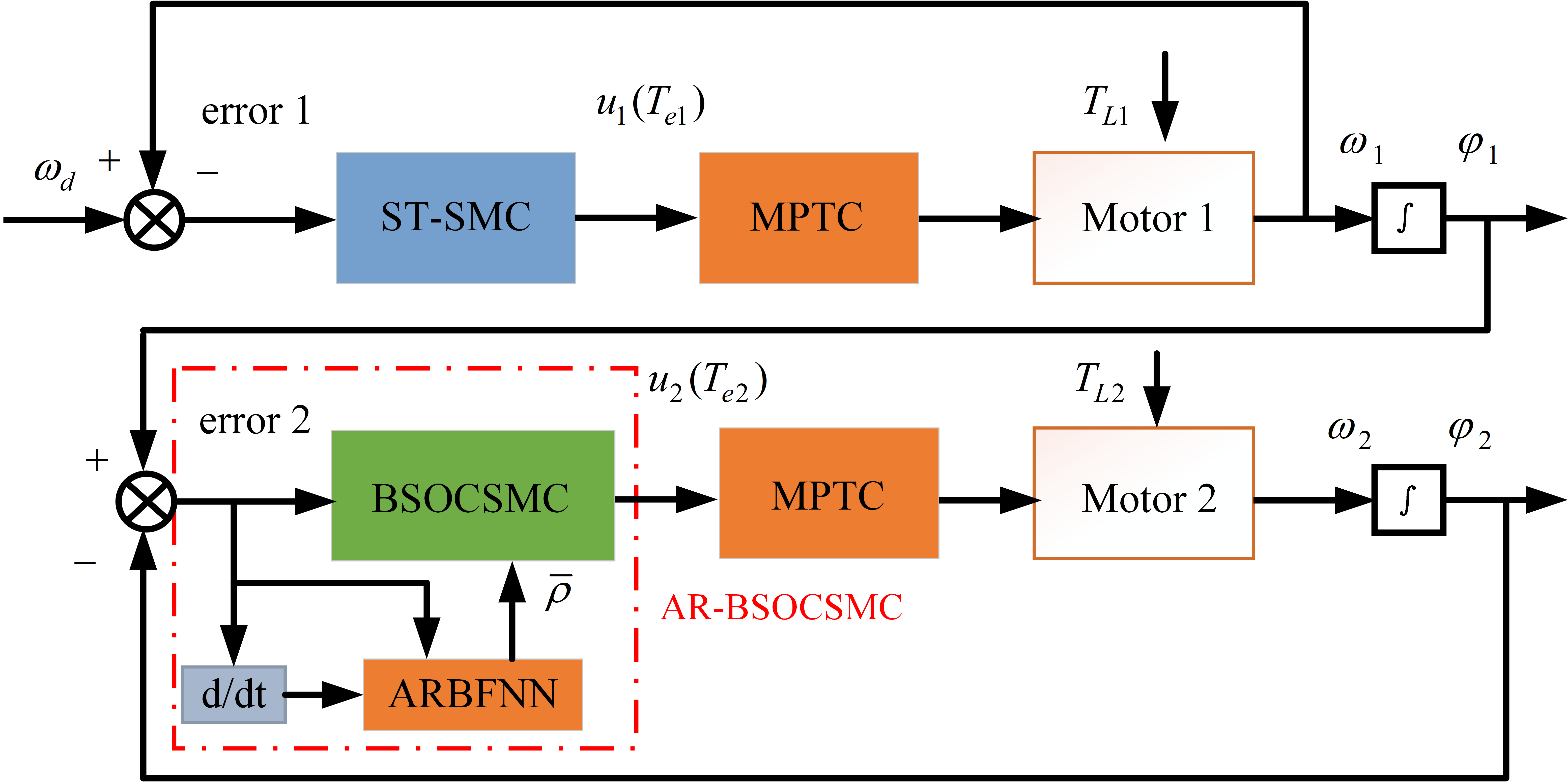

Supplement: S2 Fig — (ZIP) [file pone.0294726.s002.zip › S2 Fig.tif]

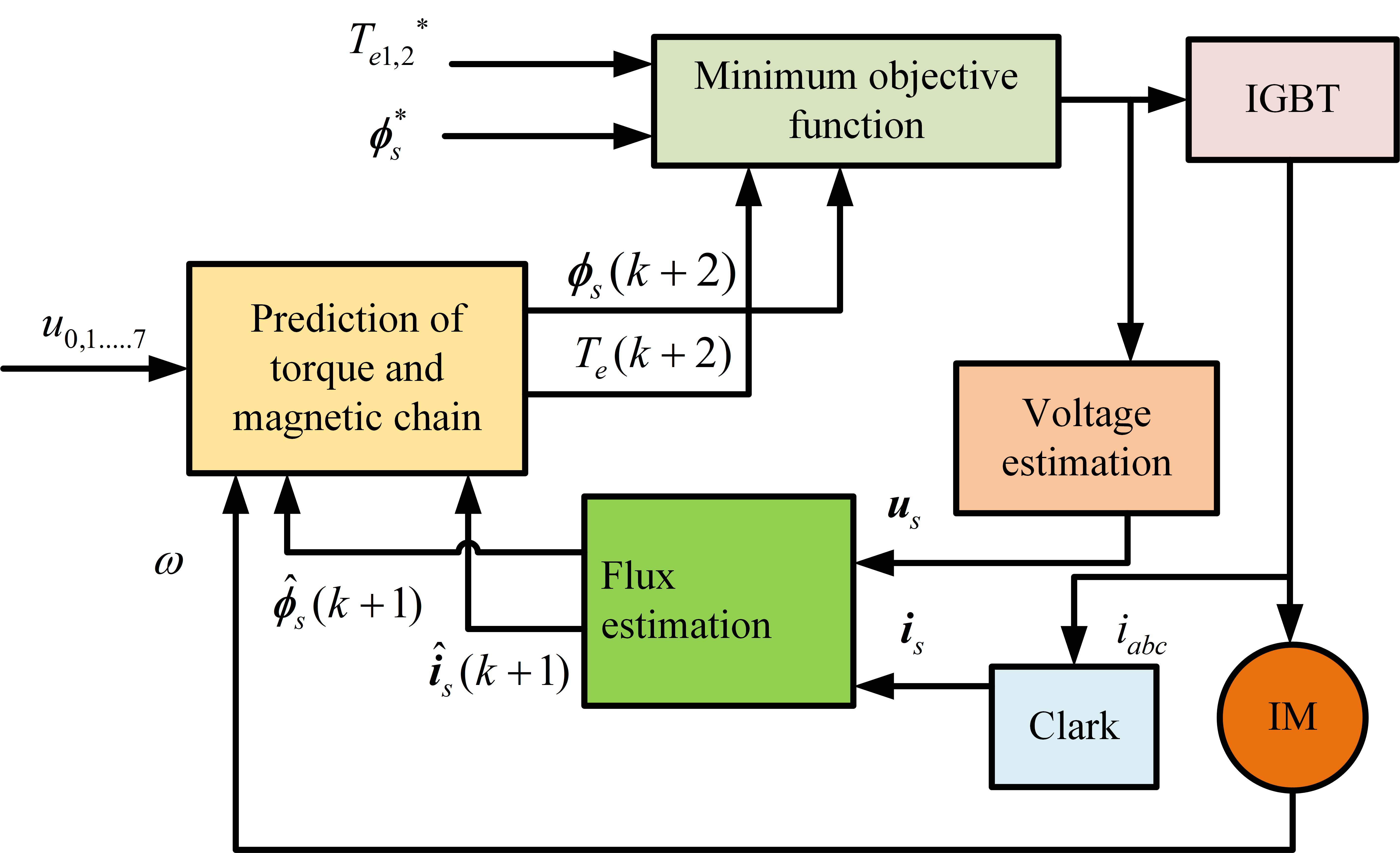

Supplement: S3 Fig — (ZIP) [file pone.0294726.s003.zip › S3 Fig.tif]

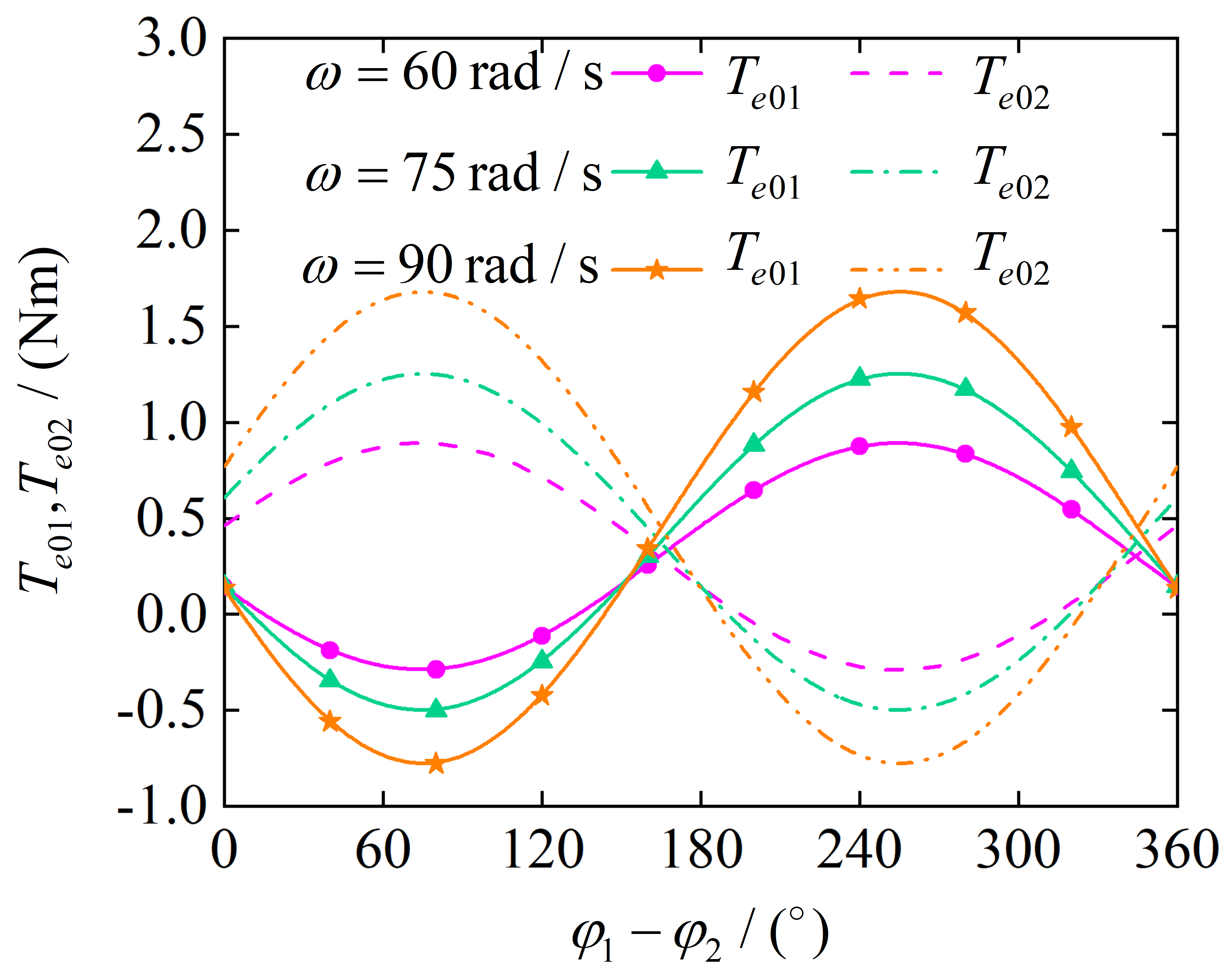

Supplement: S4 Fig — (ZIP) [file pone.0294726.s004.zip › (a).tif]

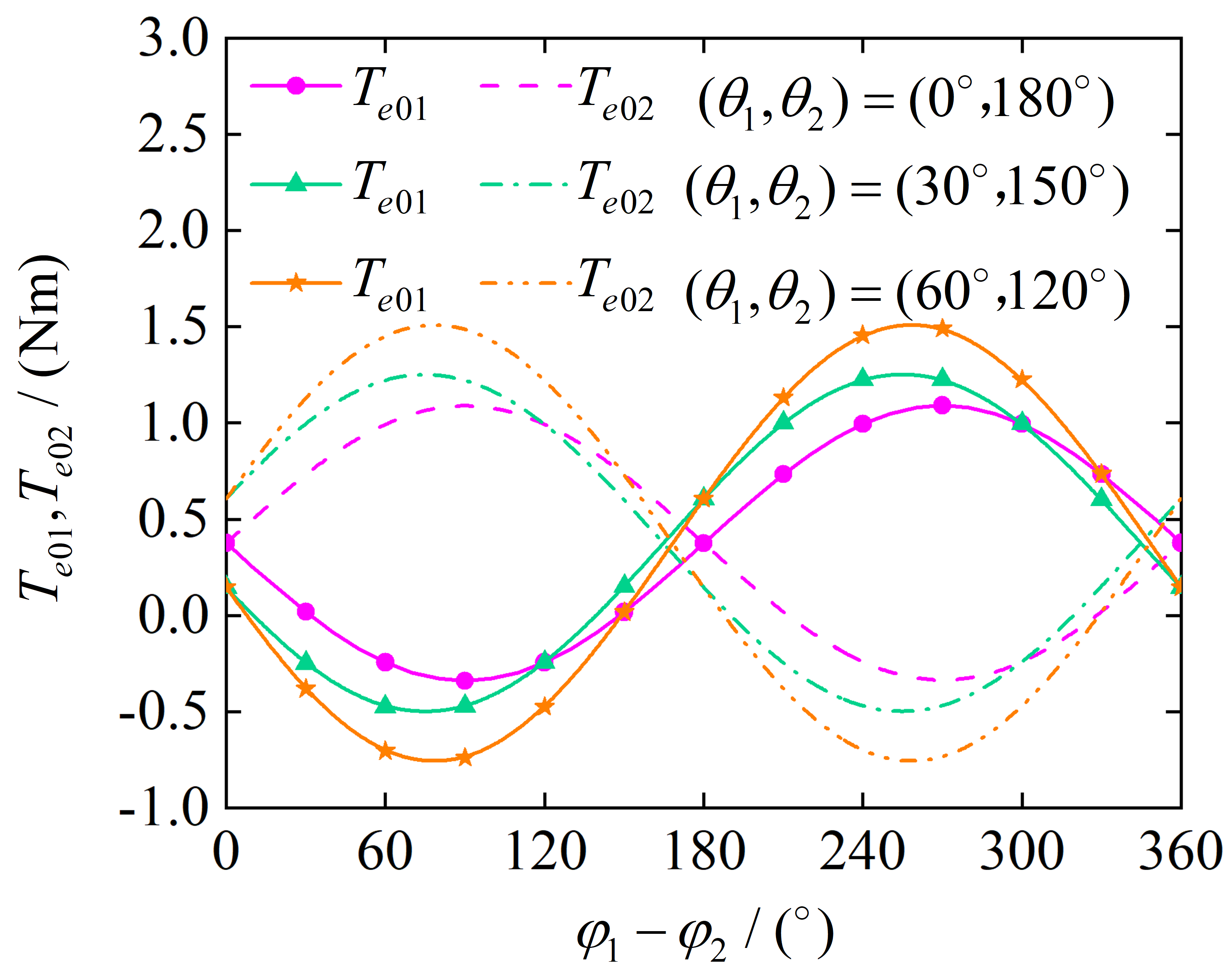

Supplement: S4 Fig — (ZIP) [file pone.0294726.s004.zip › (b).tif]

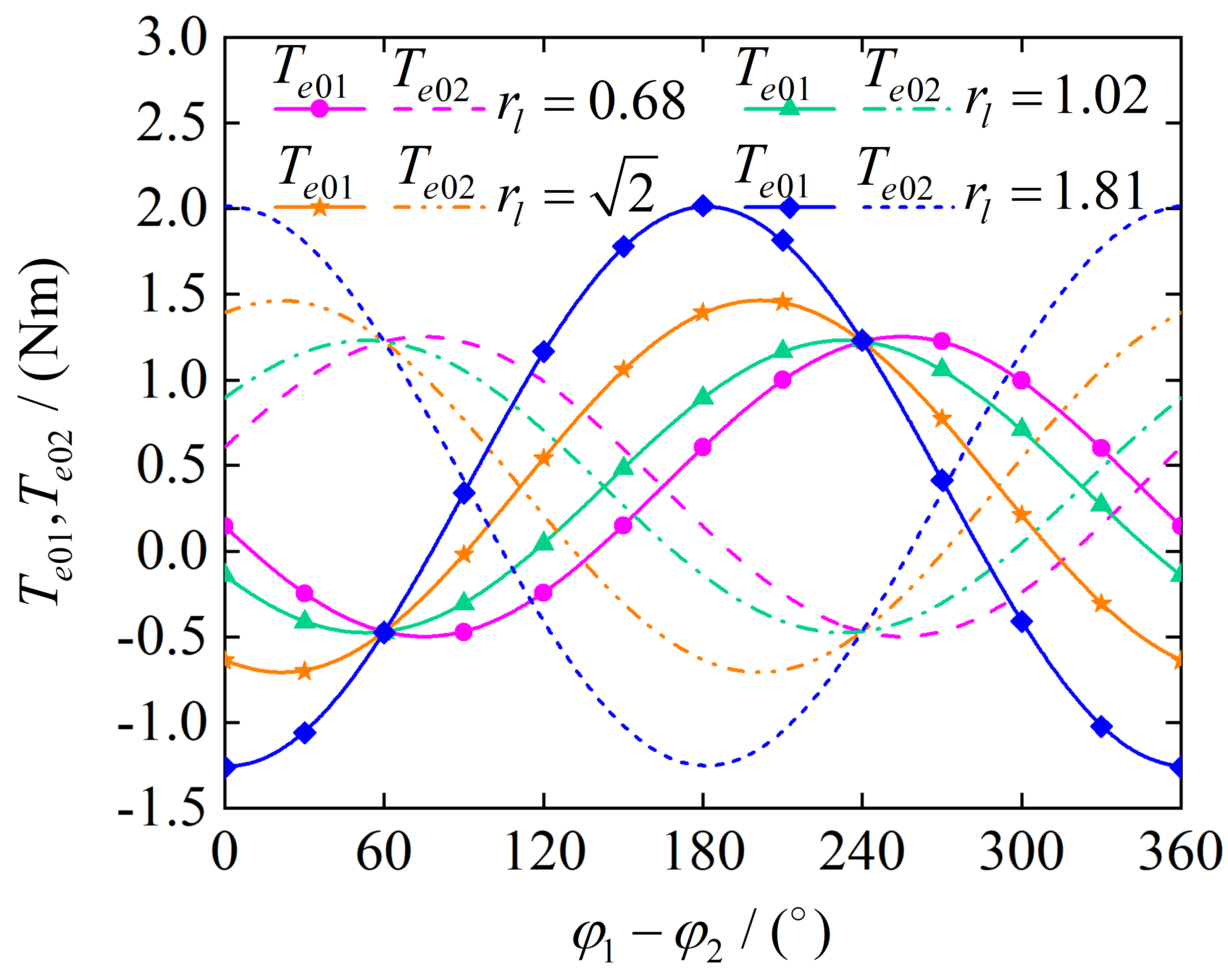

Supplement: S4 Fig — (ZIP) [file pone.0294726.s004.zip › (c).tif]

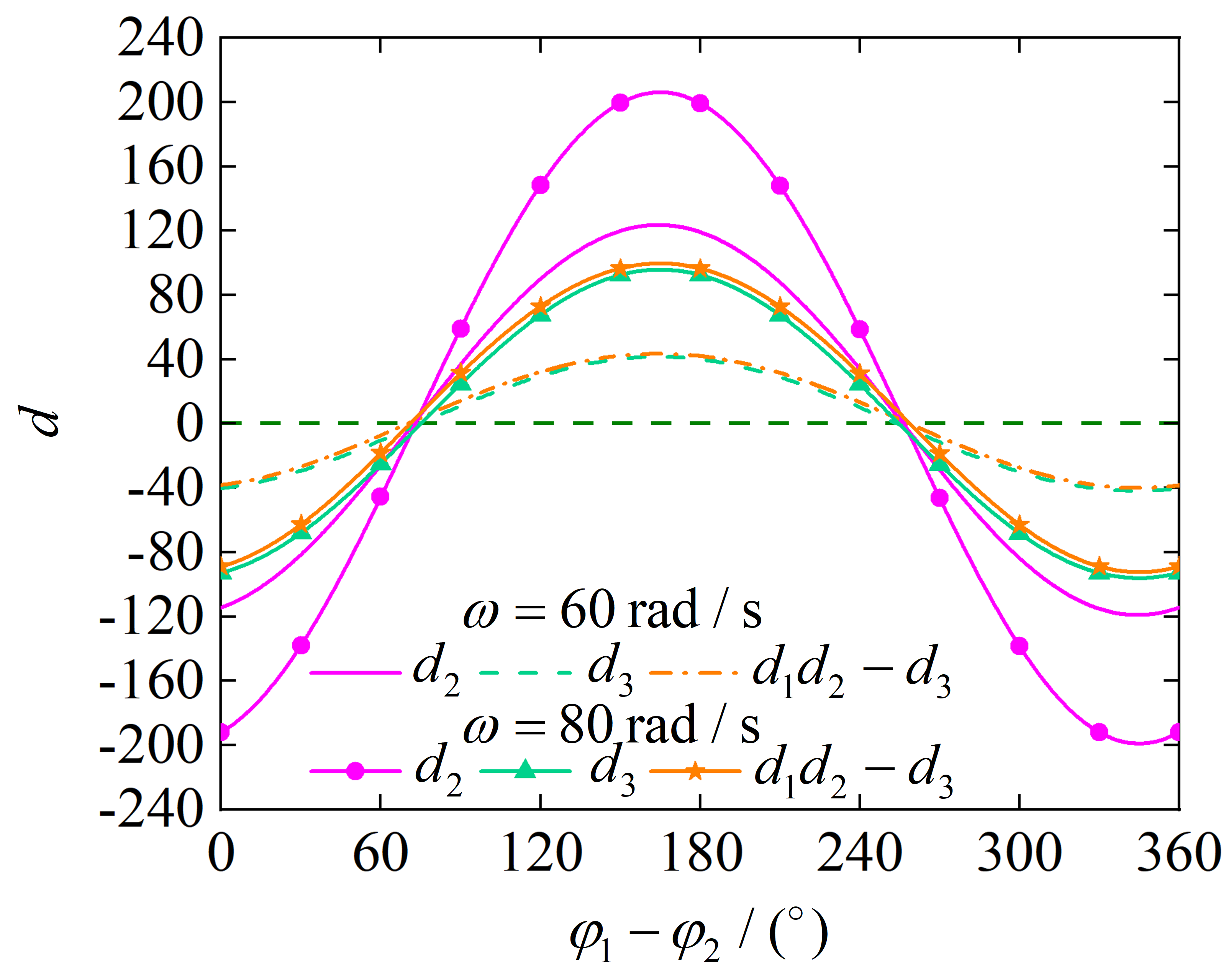

Supplement: S4 Fig — (ZIP) [file pone.0294726.s004.zip › (d).tif]

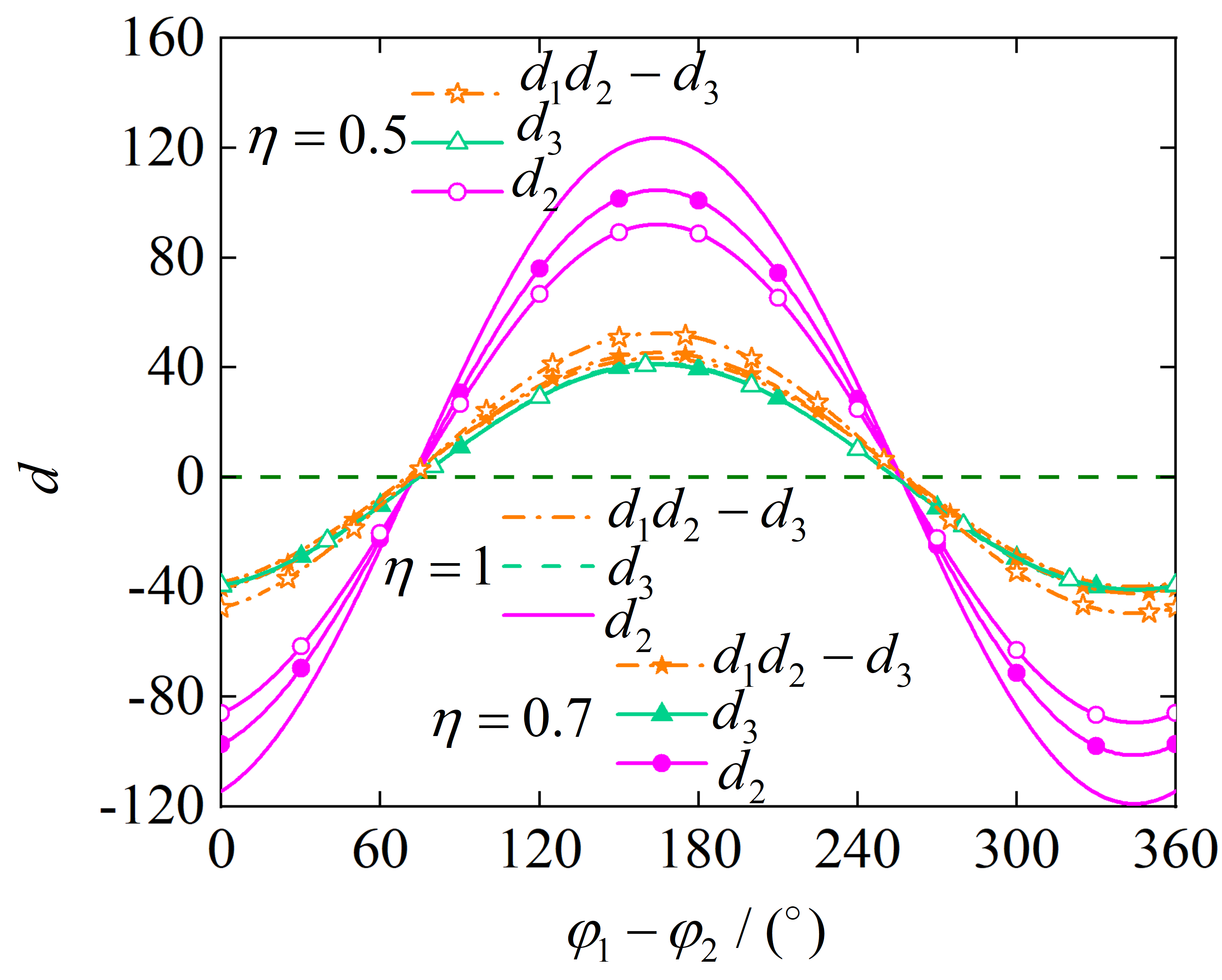

Supplement: S4 Fig — (ZIP) [file pone.0294726.s004.zip › (e).tif]

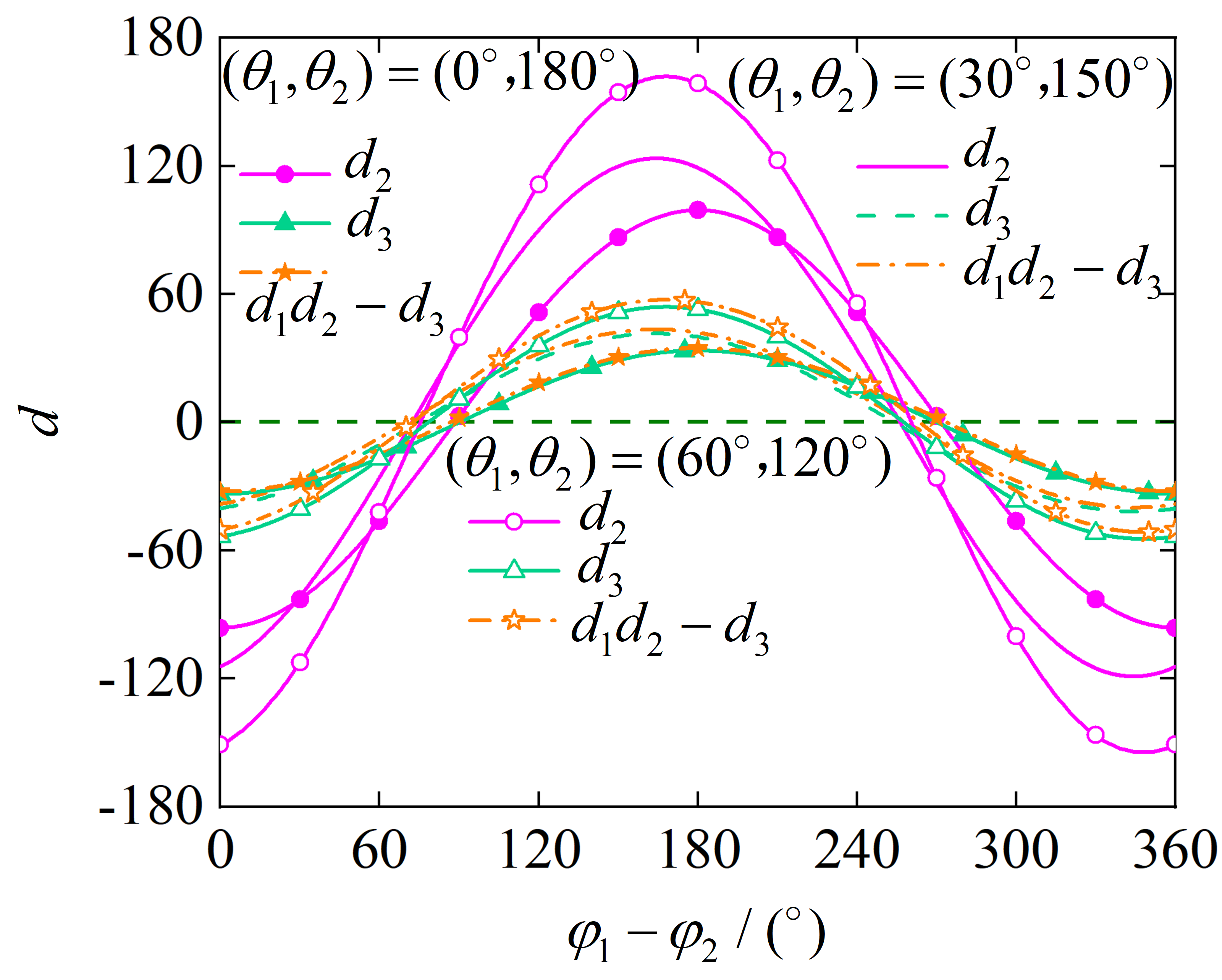

Supplement: S4 Fig — (ZIP) [file pone.0294726.s004.zip › (f).tif]

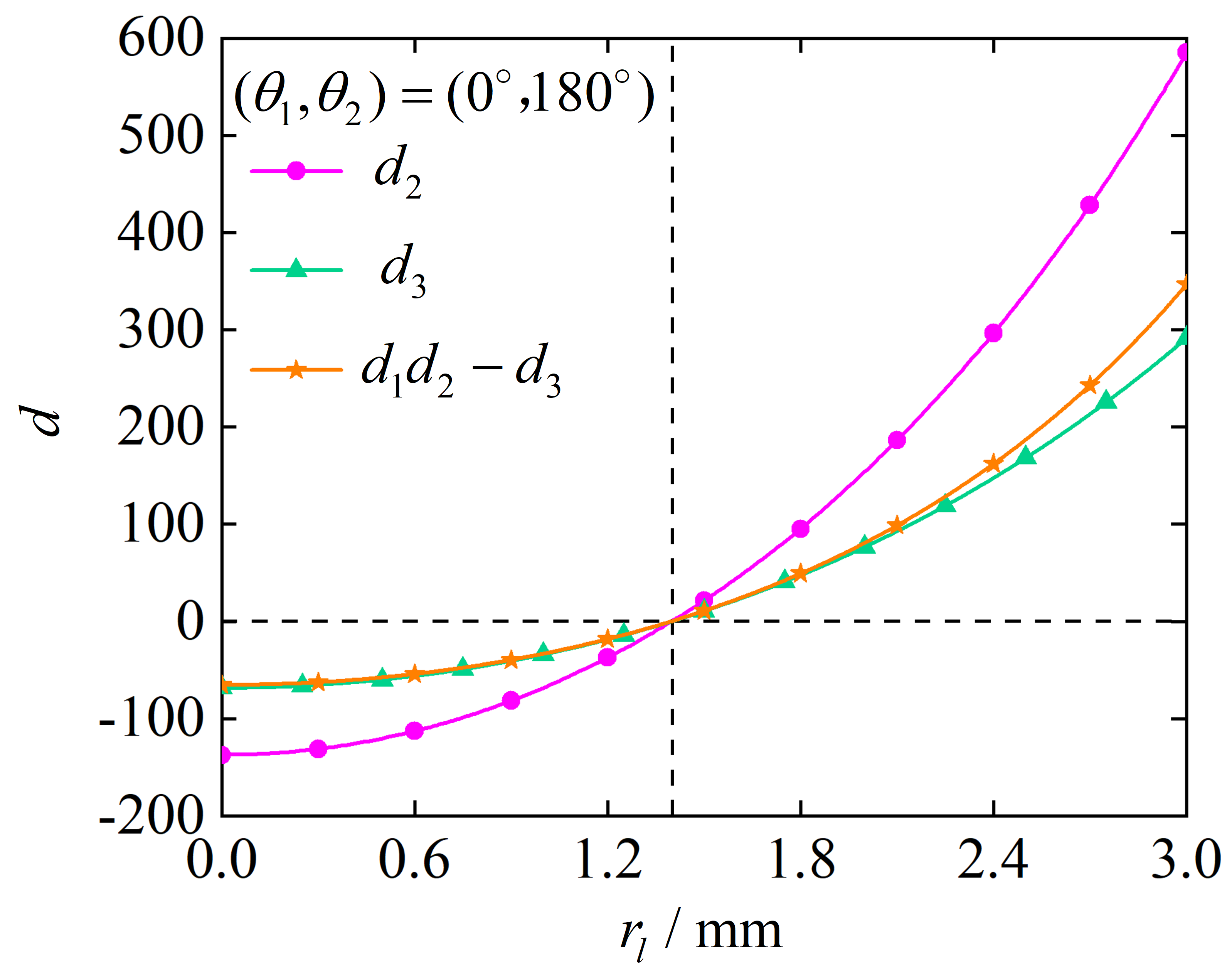

Supplement: S5 Fig — (ZIP) [file pone.0294726.s005.zip › (a).tif]

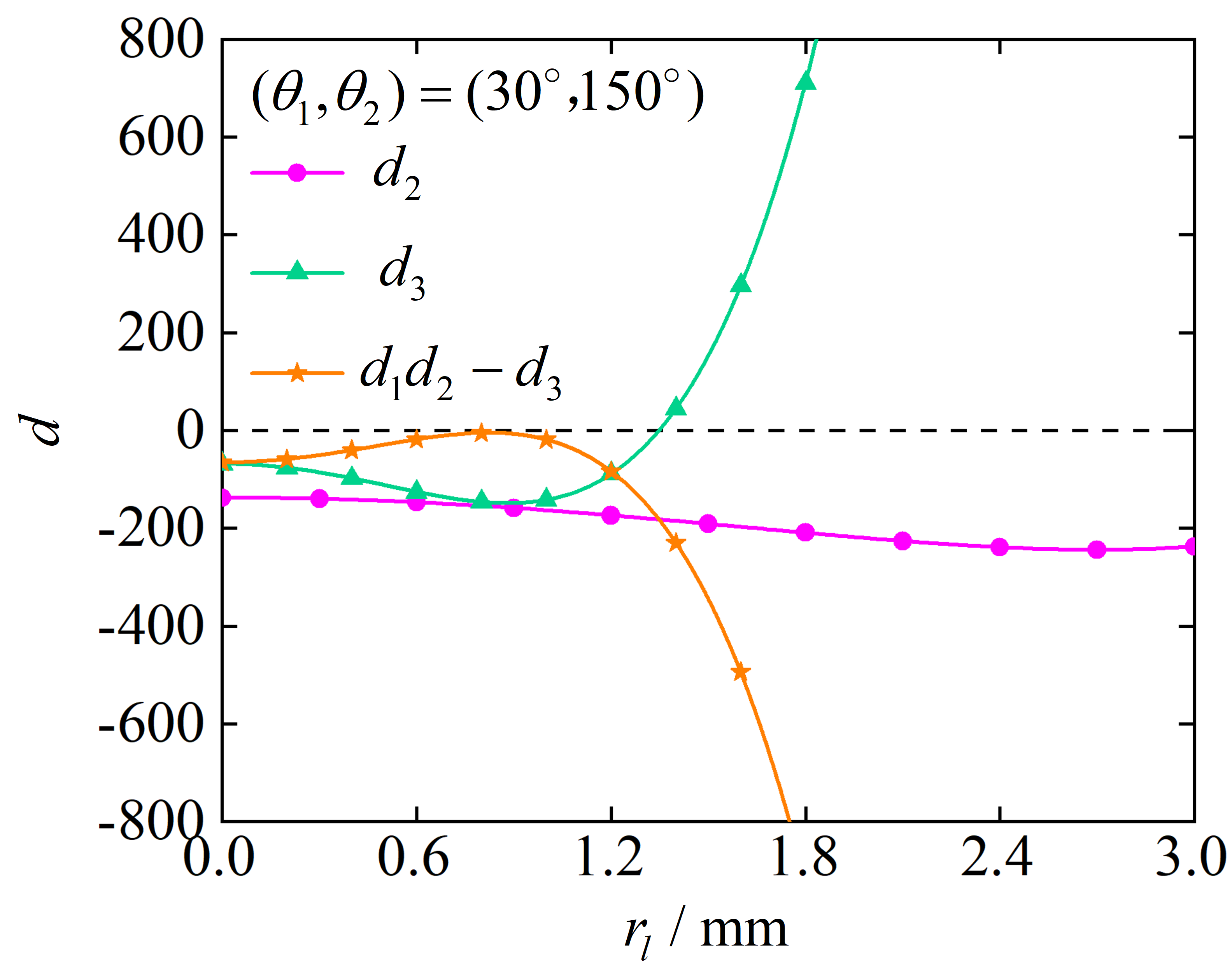

Supplement: S5 Fig — (ZIP) [file pone.0294726.s005.zip › (b).tif]

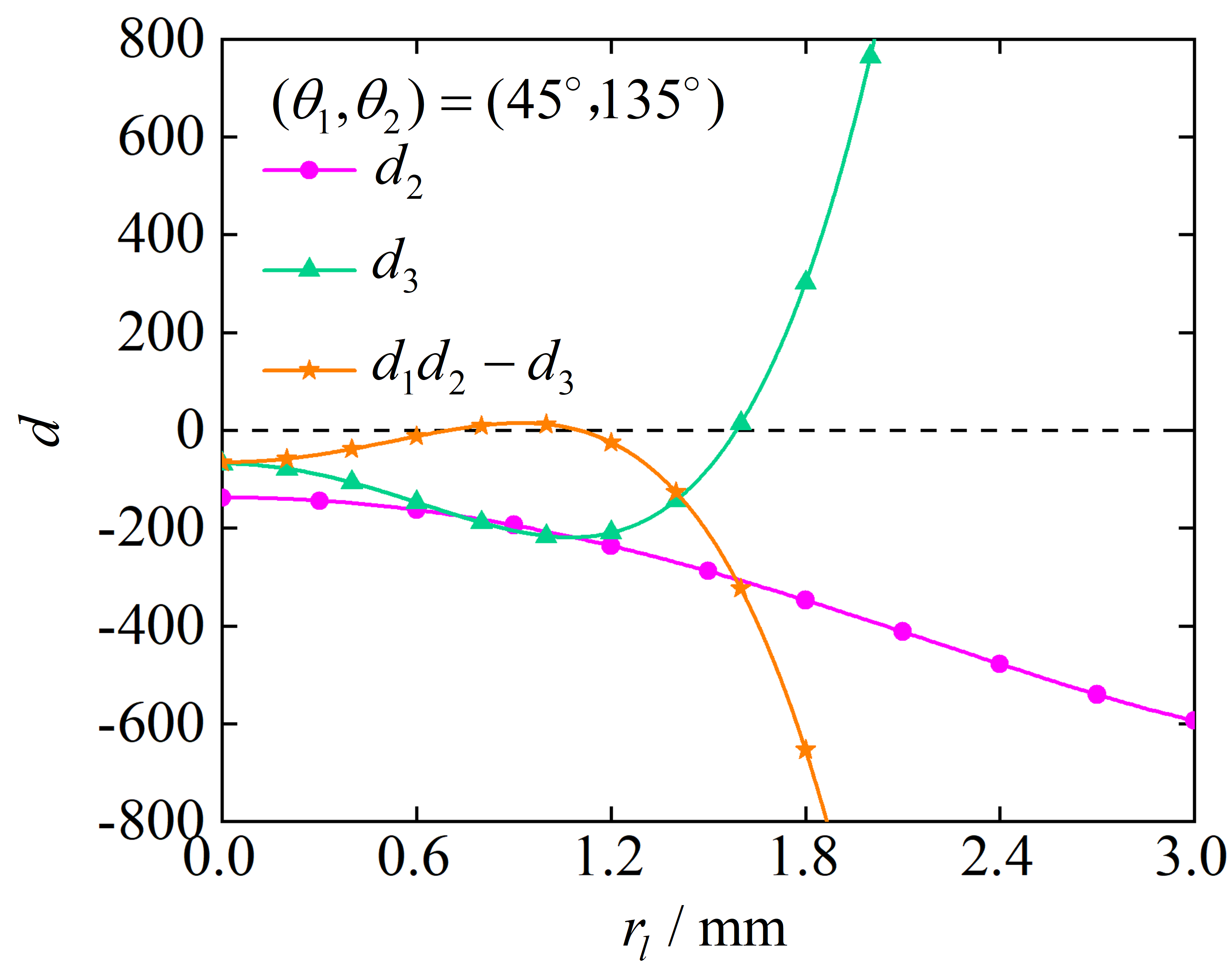

Supplement: S5 Fig — (ZIP) [file pone.0294726.s005.zip › (c).tif]

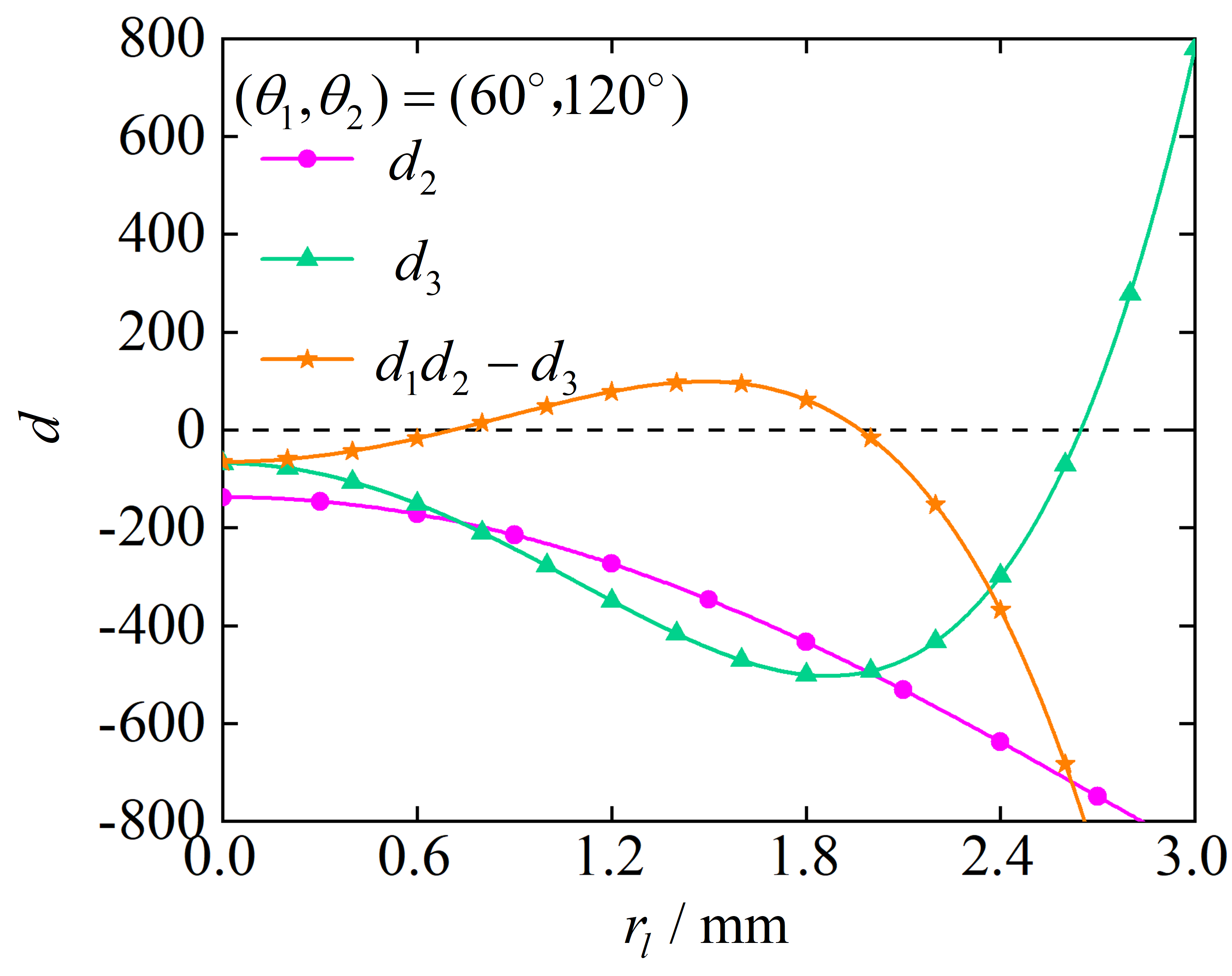

Supplement: S5 Fig — (ZIP) [file pone.0294726.s005.zip › (d).tif]

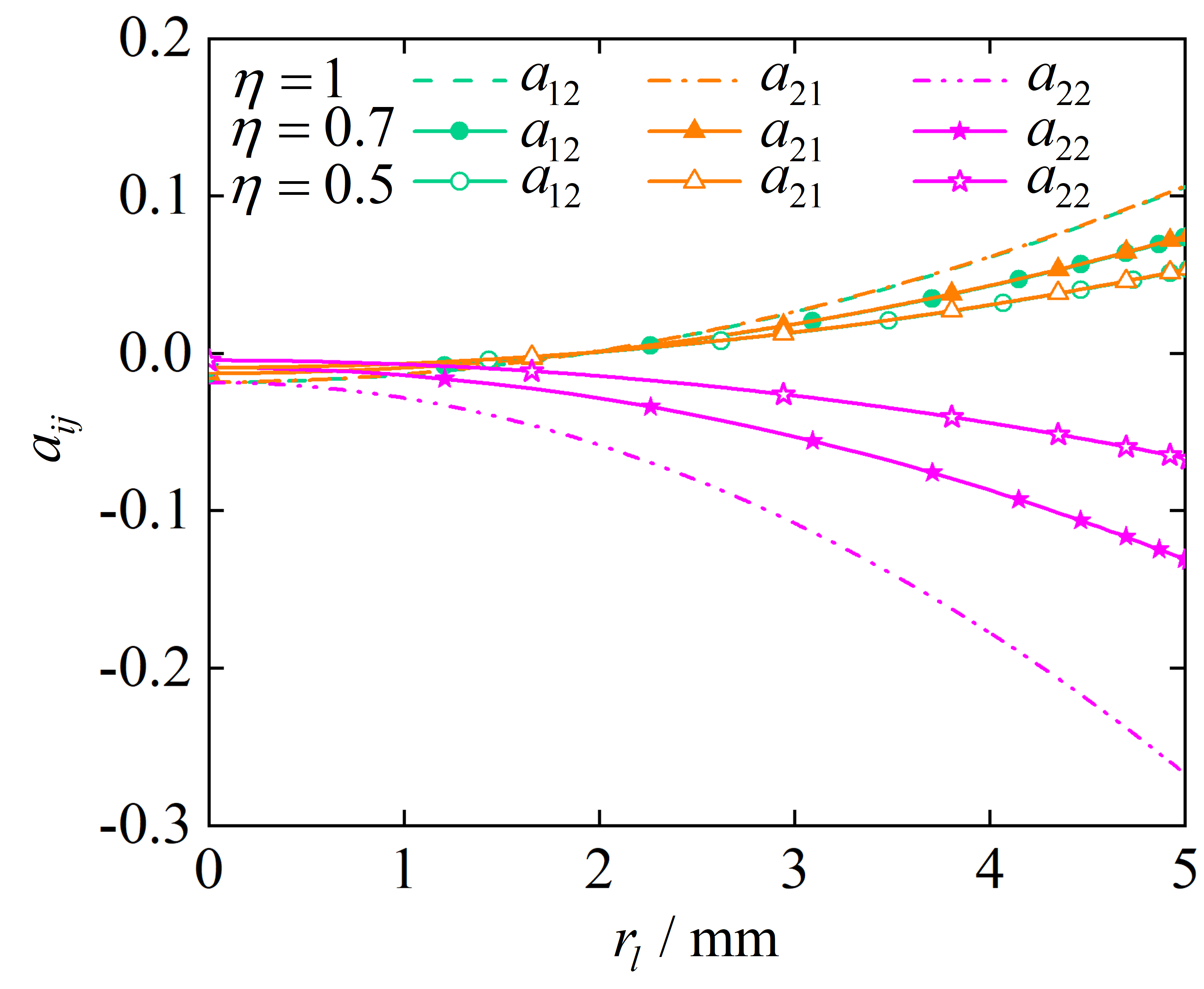

Supplement: S5 Fig — (ZIP) [file pone.0294726.s005.zip › (e).tif]

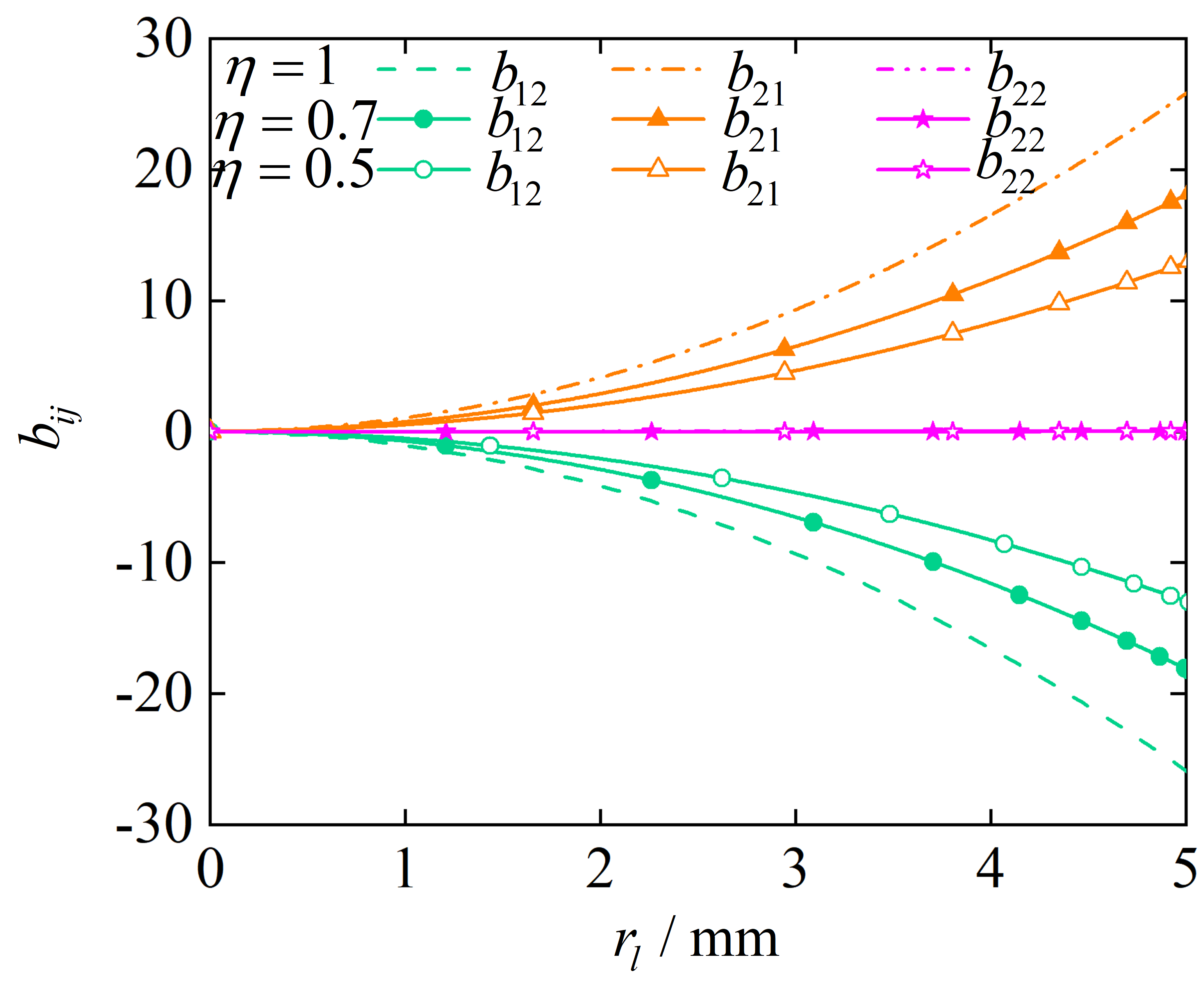

Supplement: S5 Fig — (ZIP) [file pone.0294726.s005.zip › (f).tif]

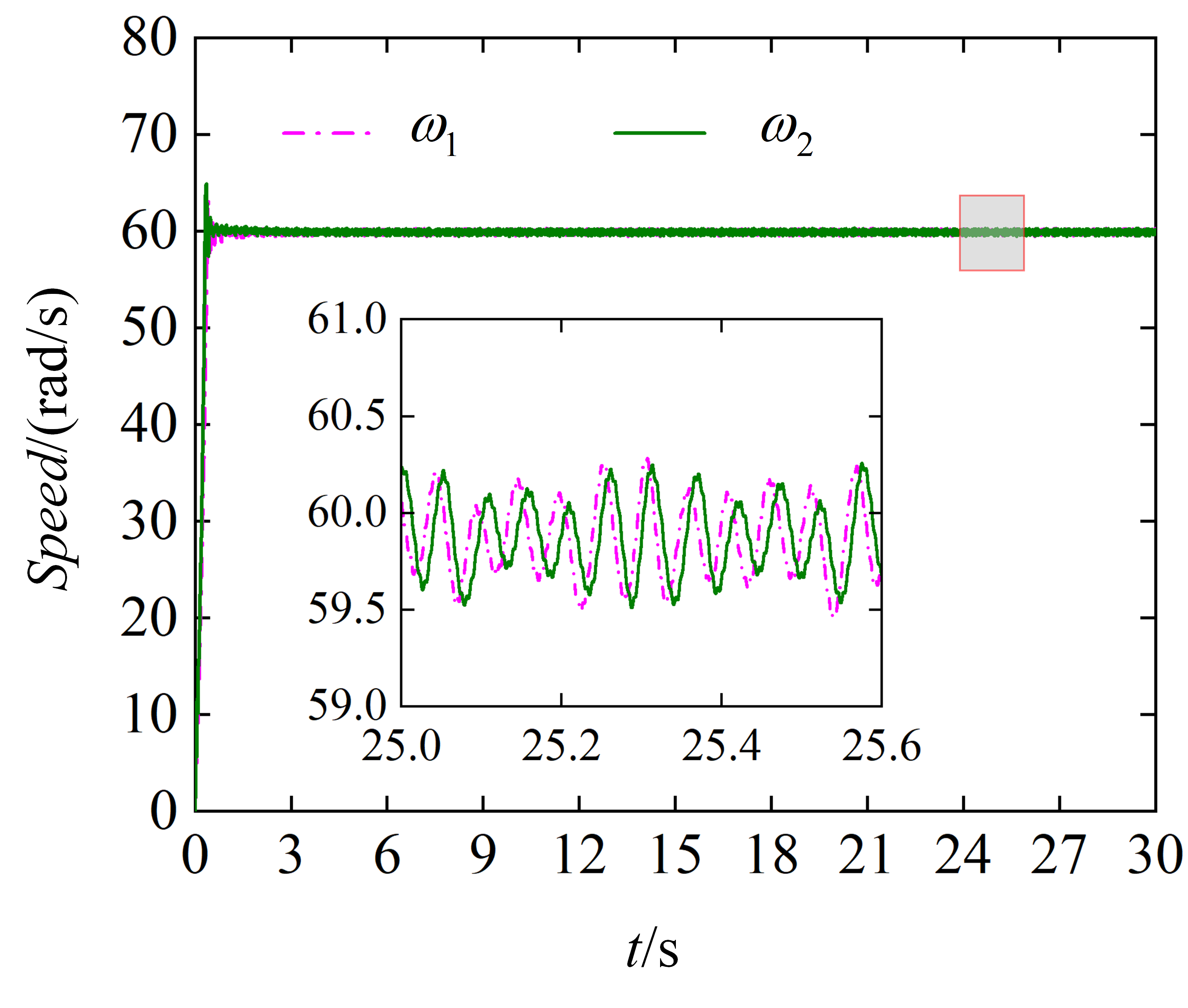

Supplement: S6 Fig — (ZIP) [file pone.0294726.s006.zip › (a).tif]

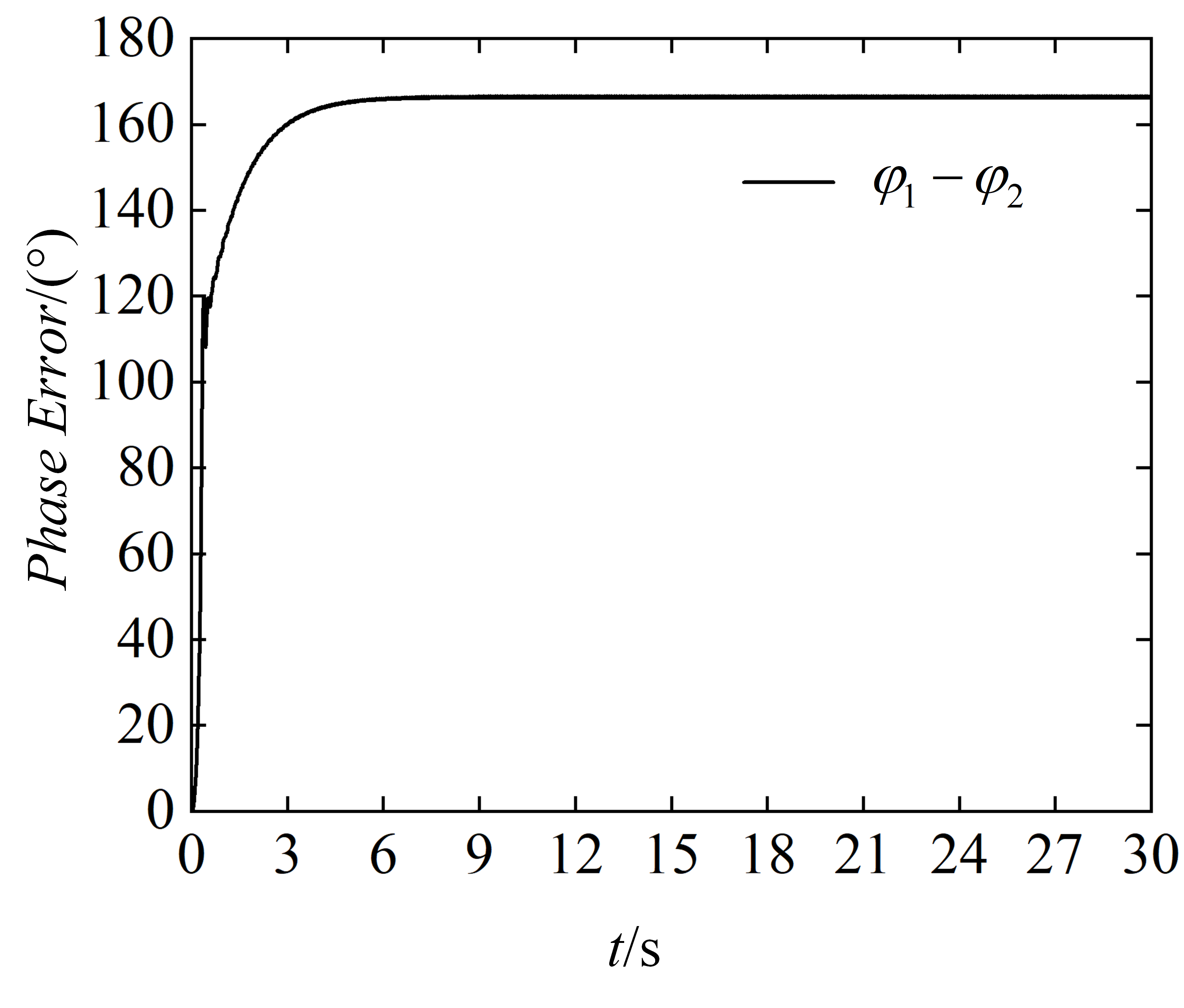

Supplement: S6 Fig — (ZIP) [file pone.0294726.s006.zip › (b).tif]

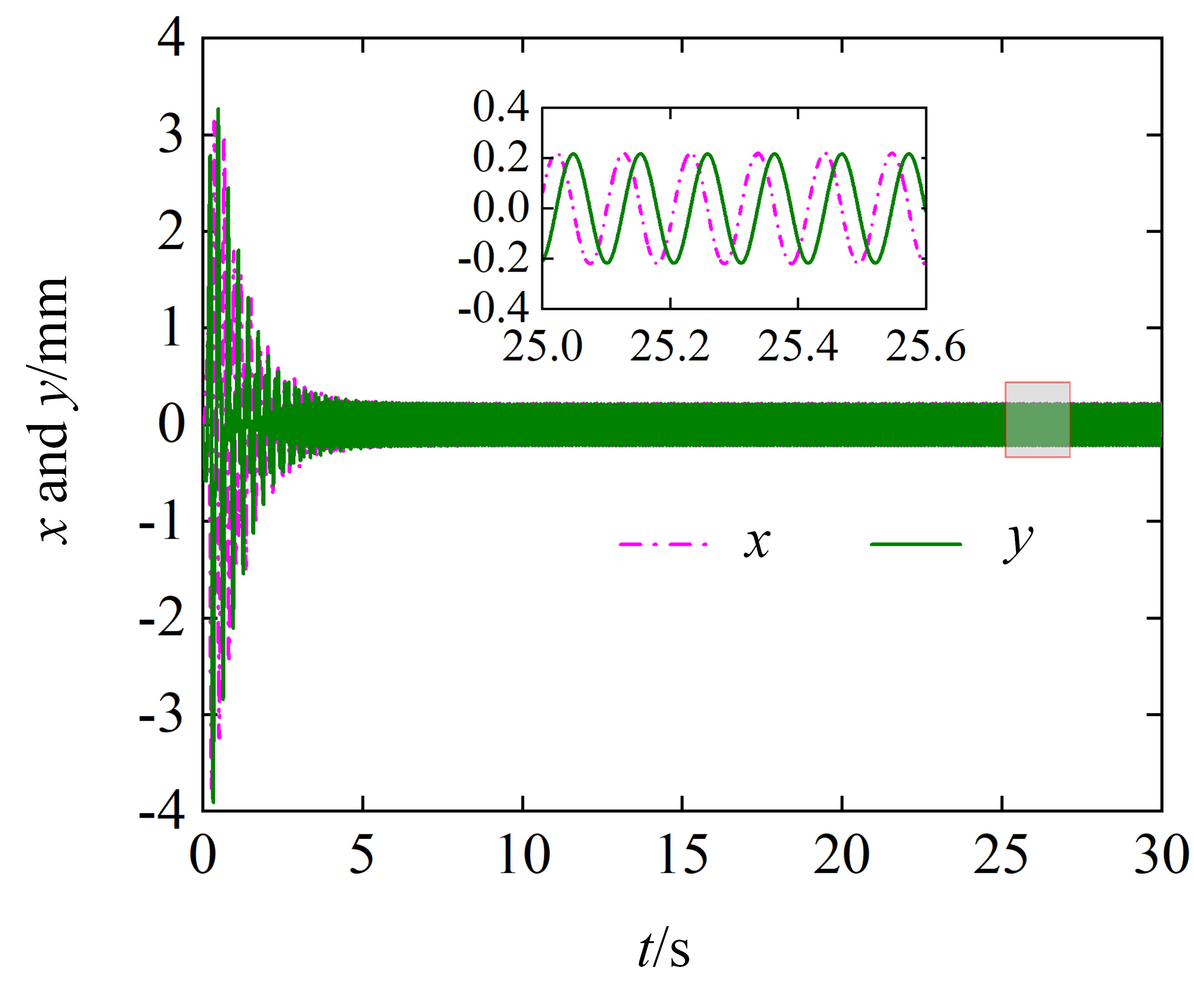

Supplement: S6 Fig — (ZIP) [file pone.0294726.s006.zip › (c).tif]

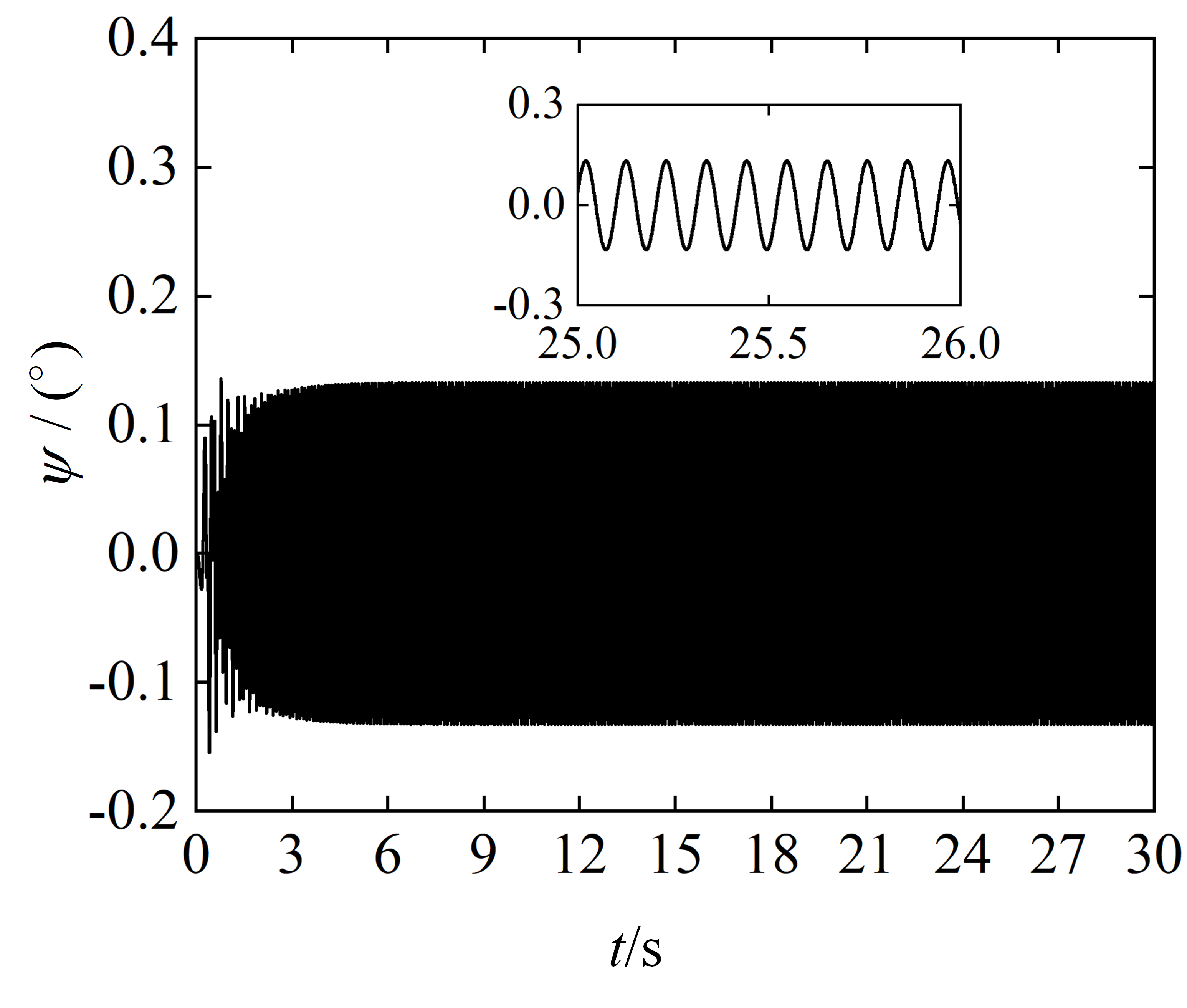

Supplement: S6 Fig — (ZIP) [file pone.0294726.s006.zip › (d).tif]

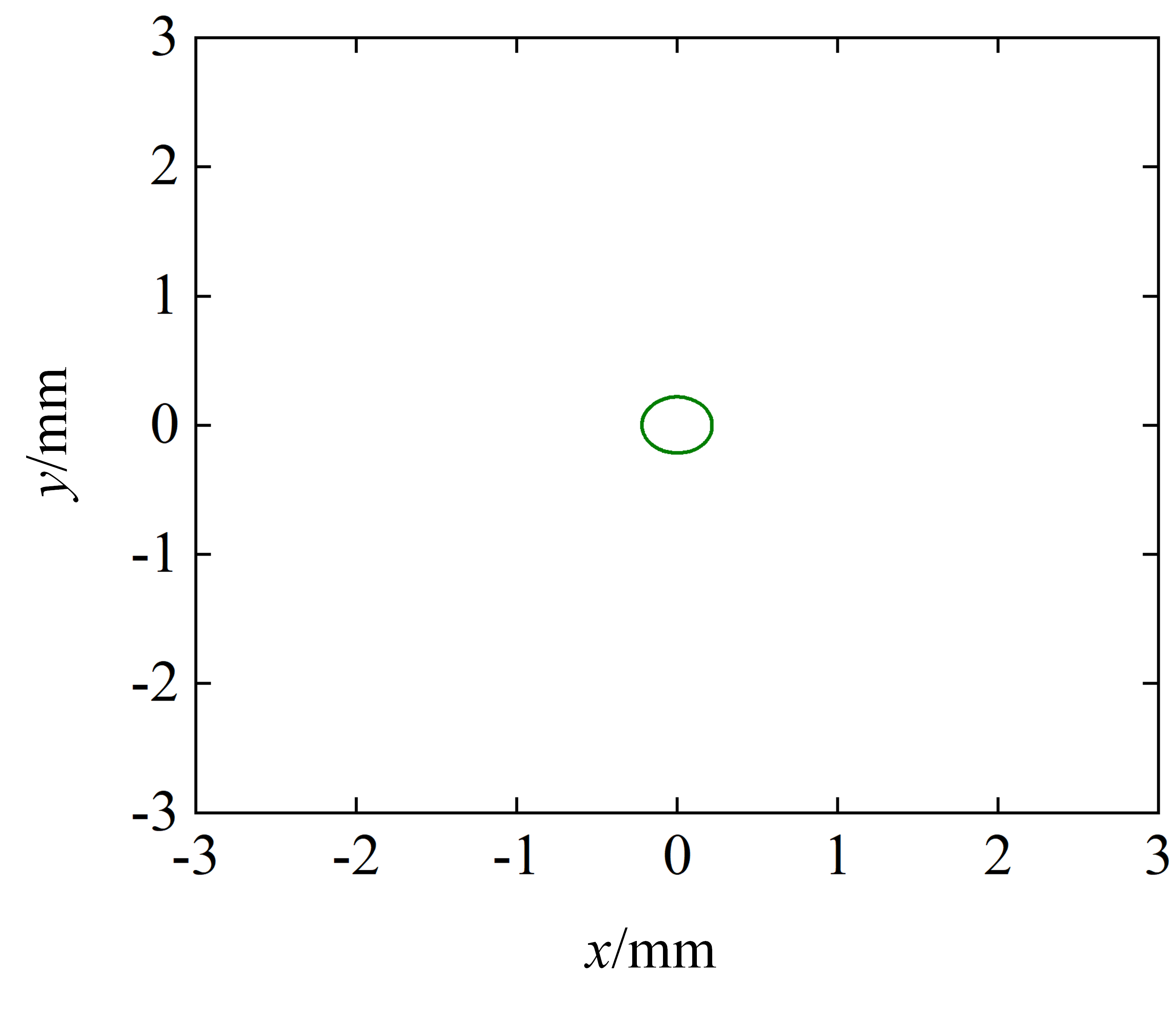

Supplement: S6 Fig — (ZIP) [file pone.0294726.s006.zip › (e).tif]

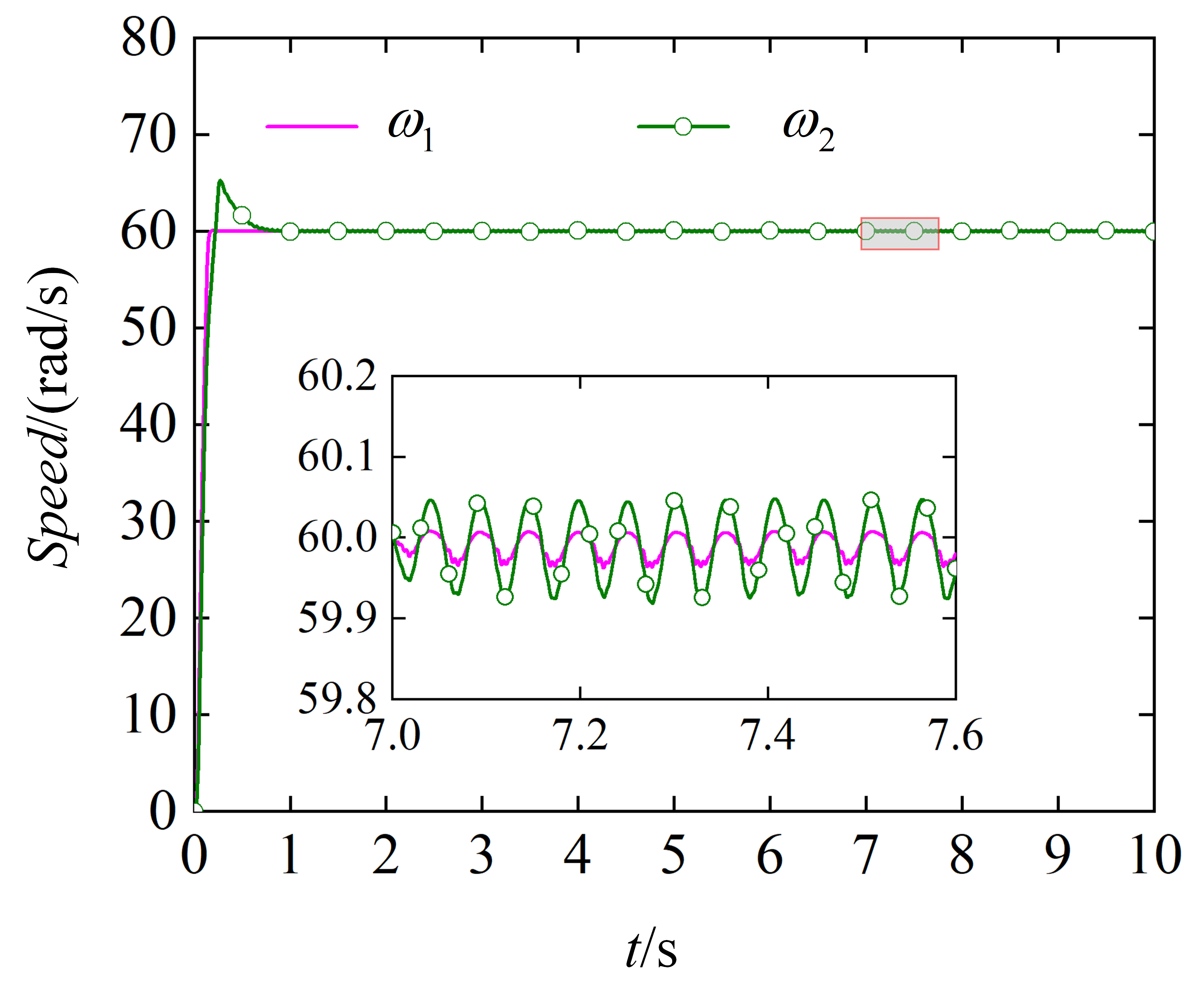

Supplement: S7 Fig — (ZIP) [file pone.0294726.s007.zip › (a).tif]

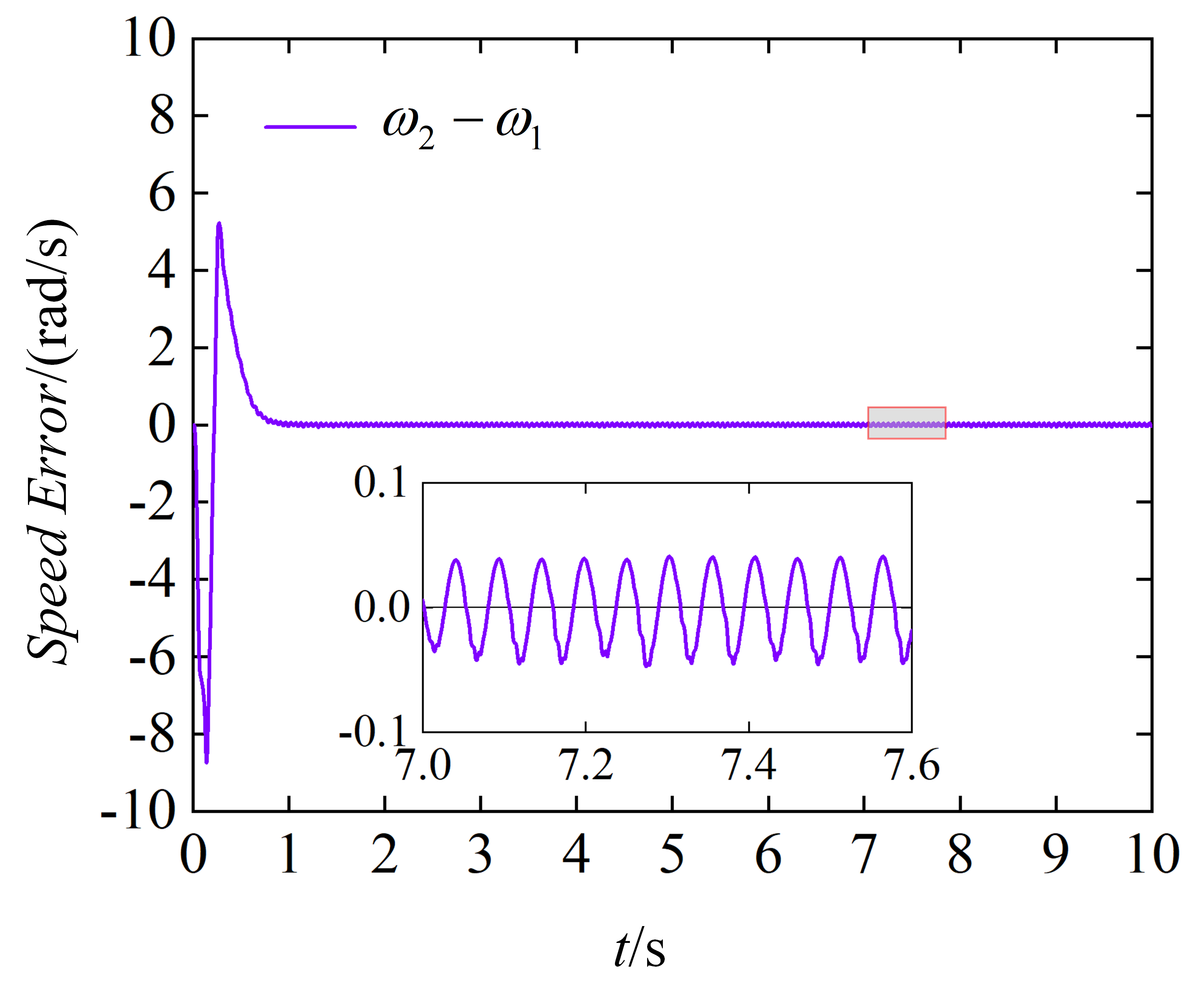

Supplement: S7 Fig — (ZIP) [file pone.0294726.s007.zip › (b).tif]

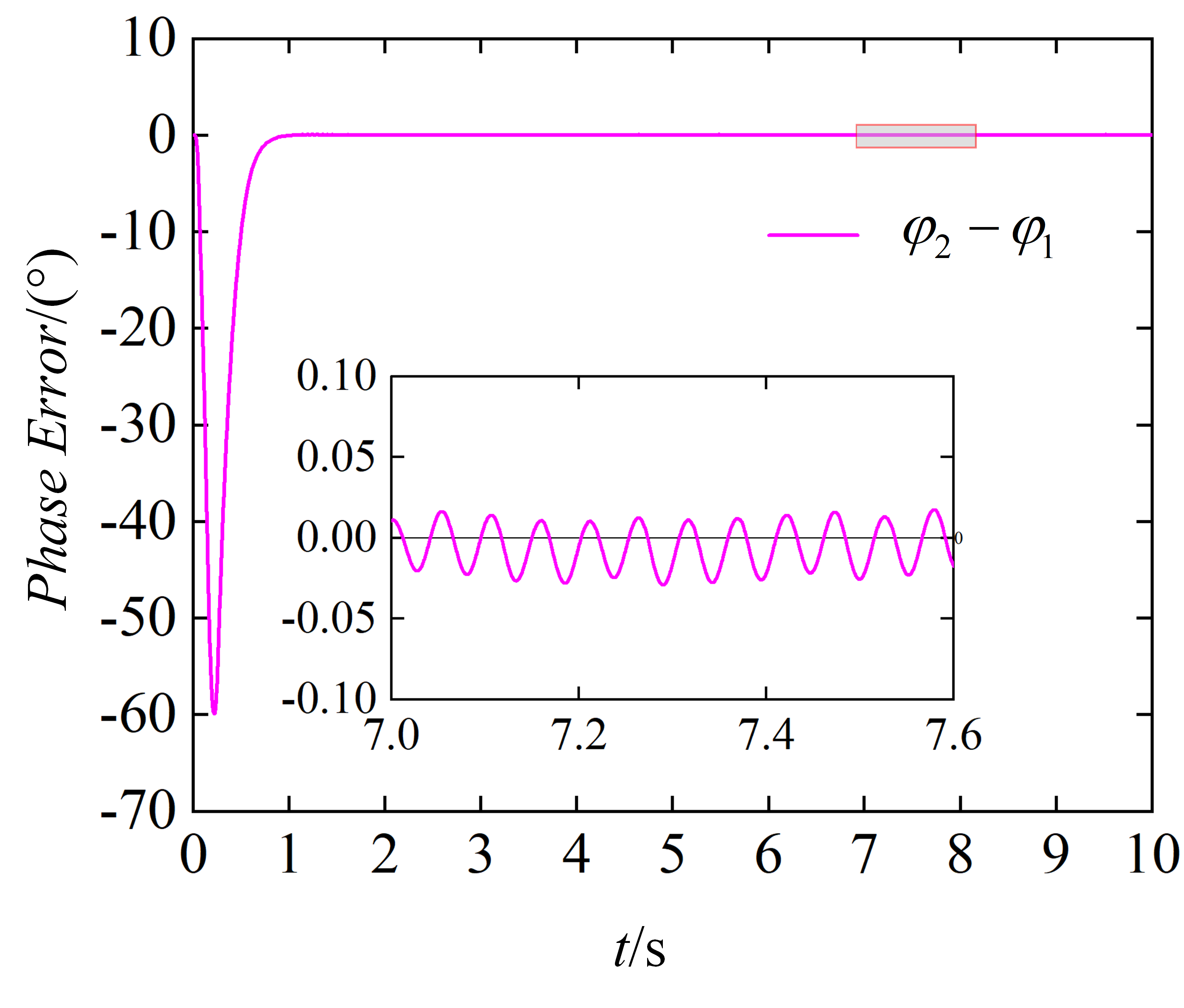

Supplement: S7 Fig — (ZIP) [file pone.0294726.s007.zip › (c).tif]

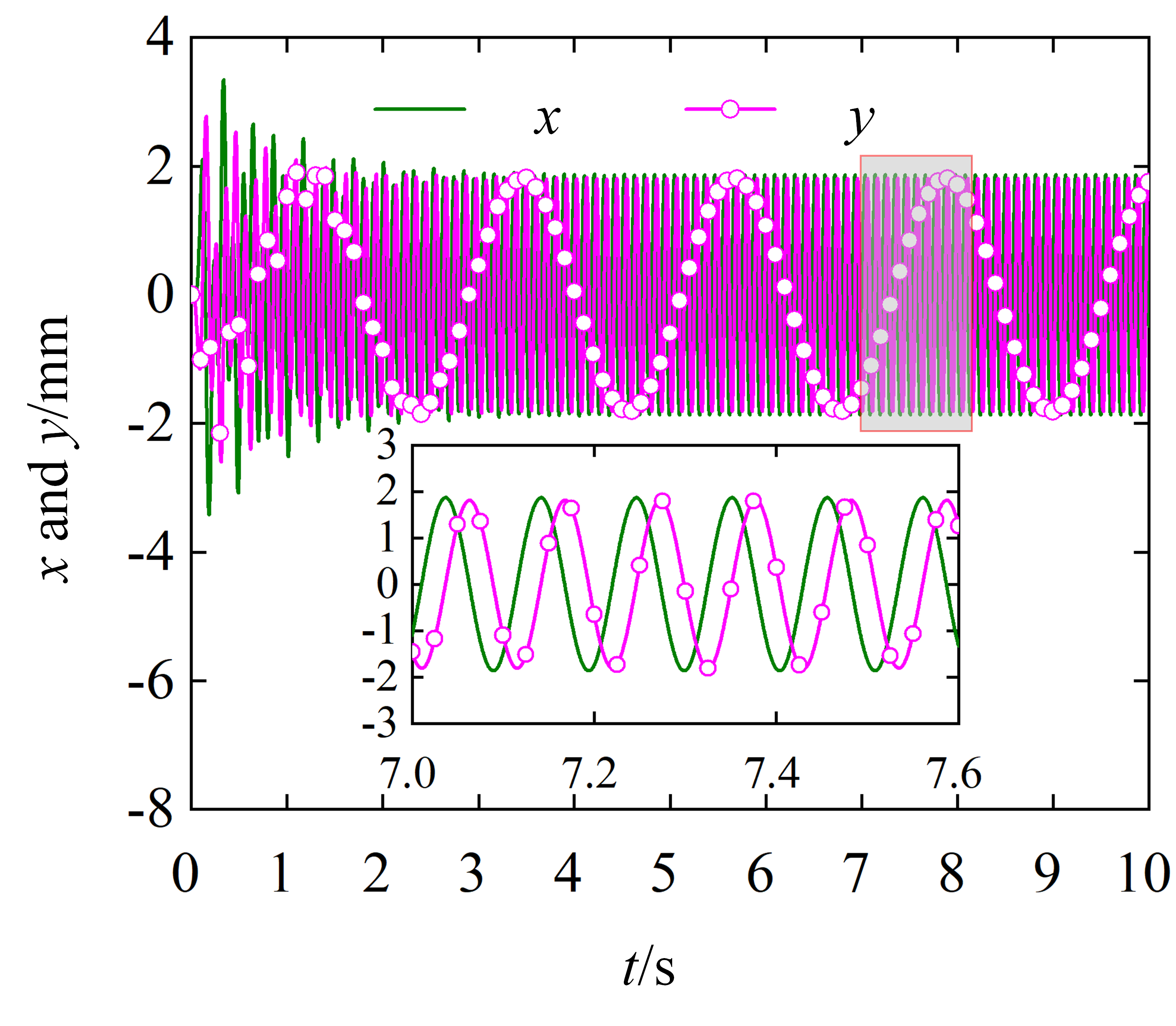

Supplement: S7 Fig — (ZIP) [file pone.0294726.s007.zip › (d).tif]

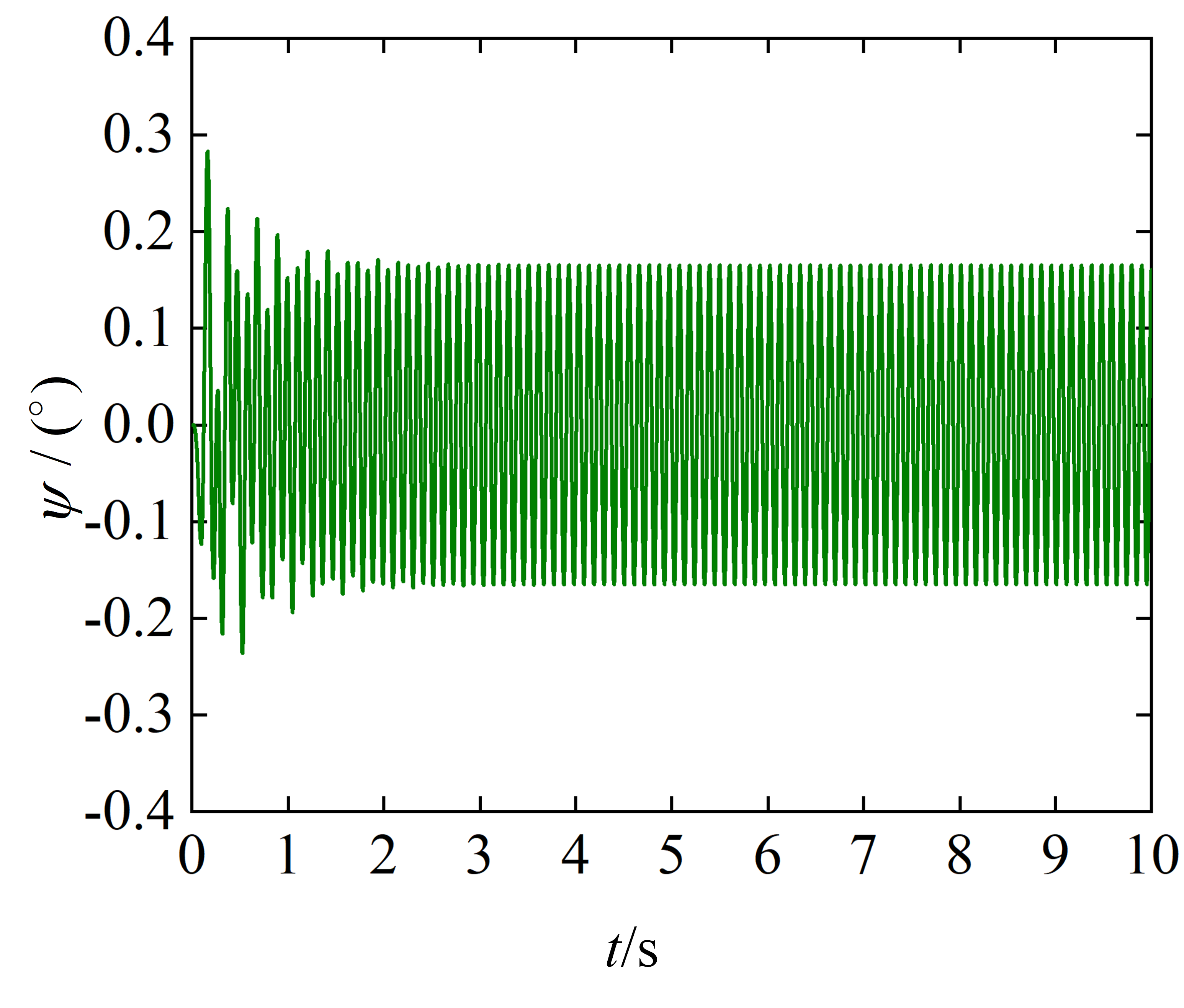

Supplement: S7 Fig — (ZIP) [file pone.0294726.s007.zip › (e).tif]

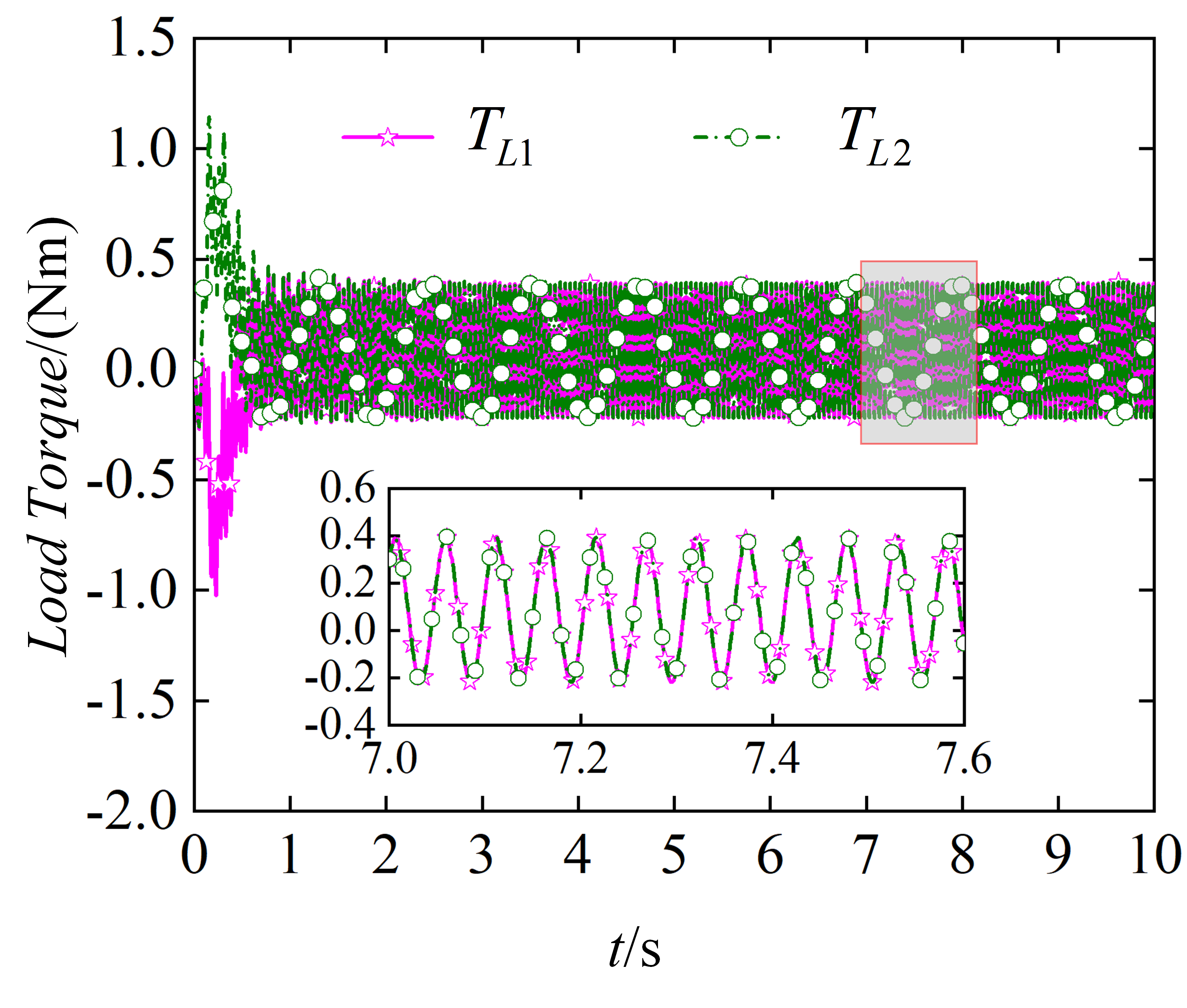

Supplement: S7 Fig — (ZIP) [file pone.0294726.s007.zip › (f).tif]

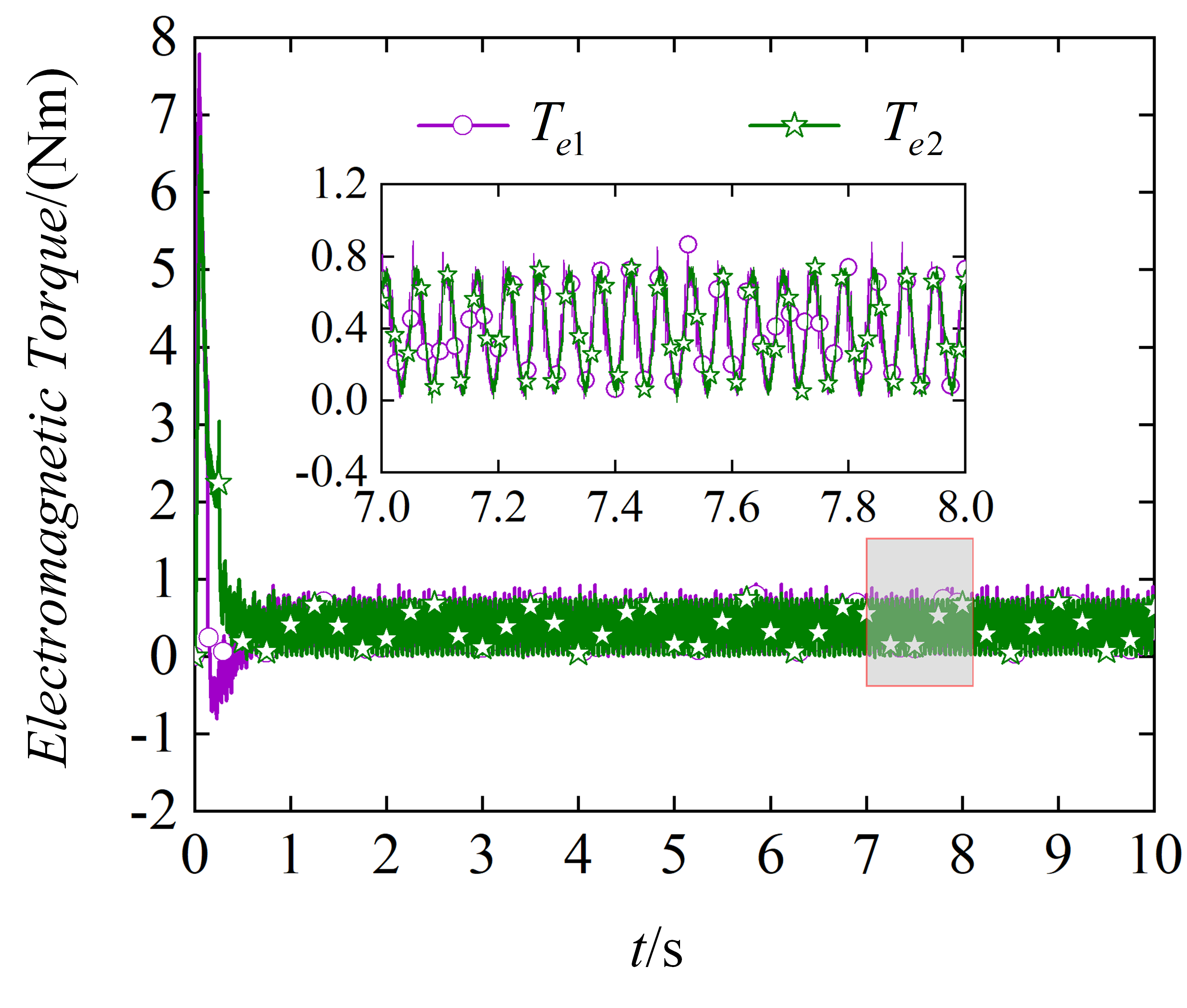

Supplement: S7 Fig — (ZIP) [file pone.0294726.s007.zip › (g).tif]

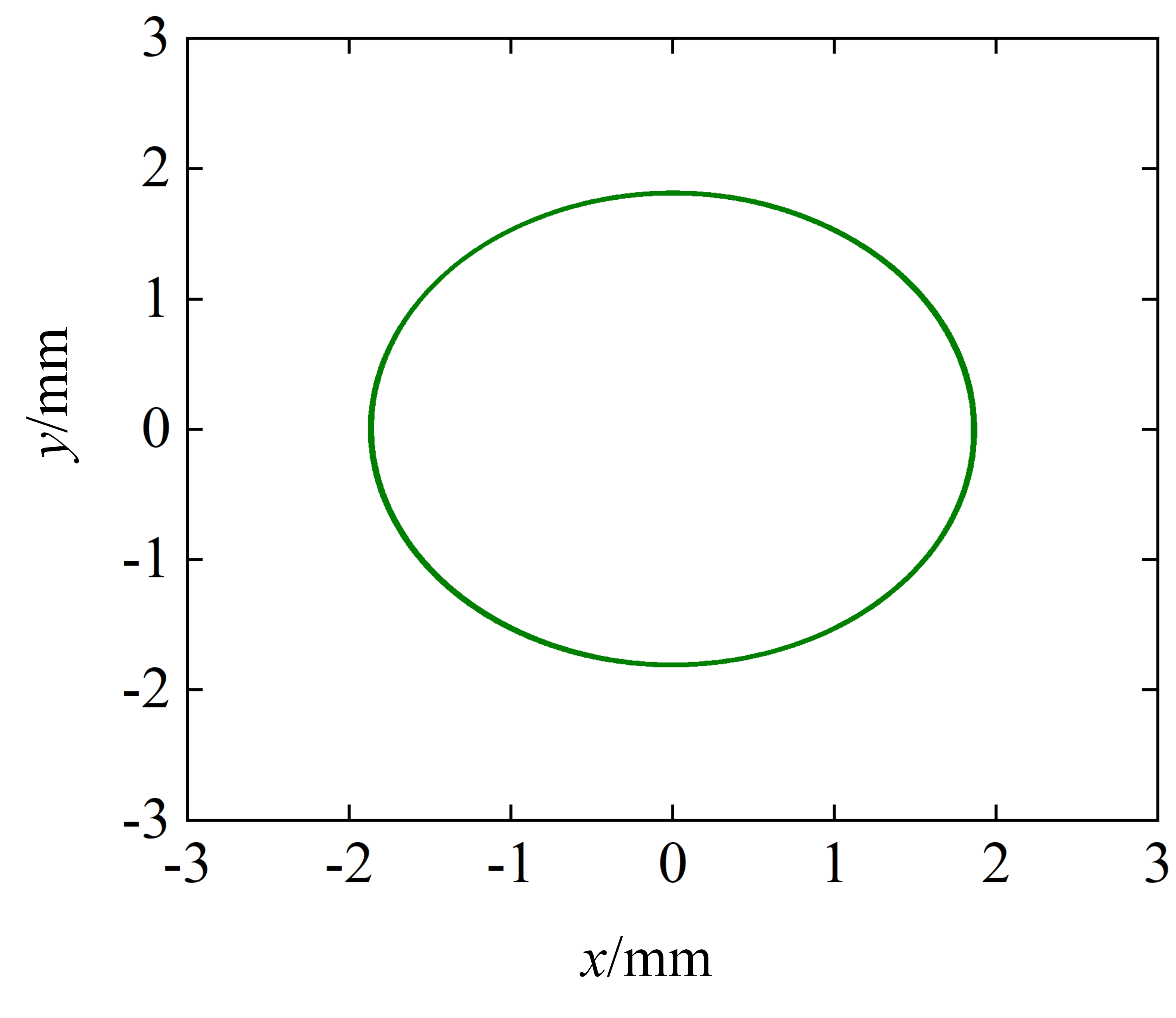

Supplement: S7 Fig — (ZIP) [file pone.0294726.s007.zip › (h).tif]

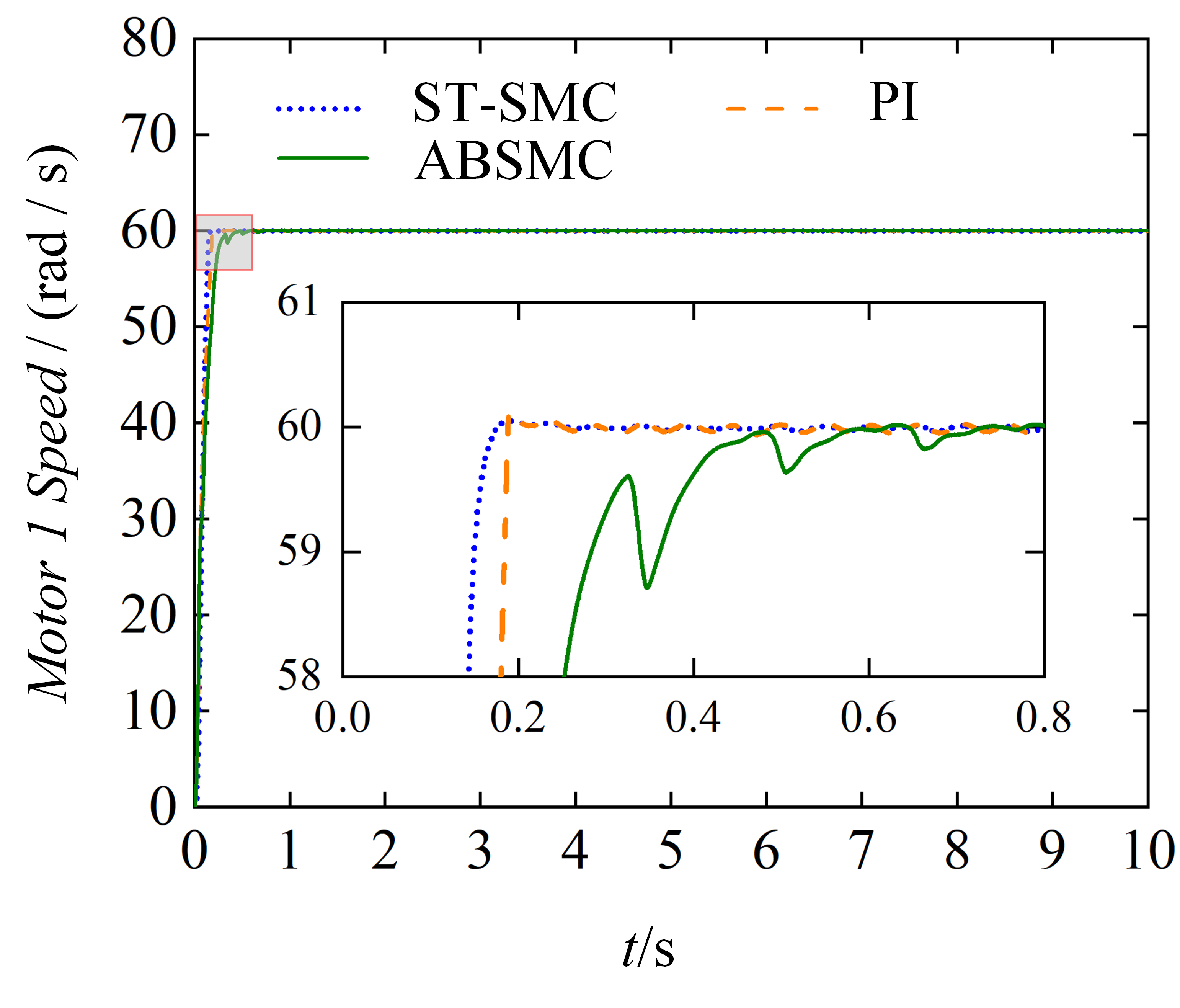

Supplement: S8 Fig — (ZIP) [file pone.0294726.s008.zip › (a).tif]

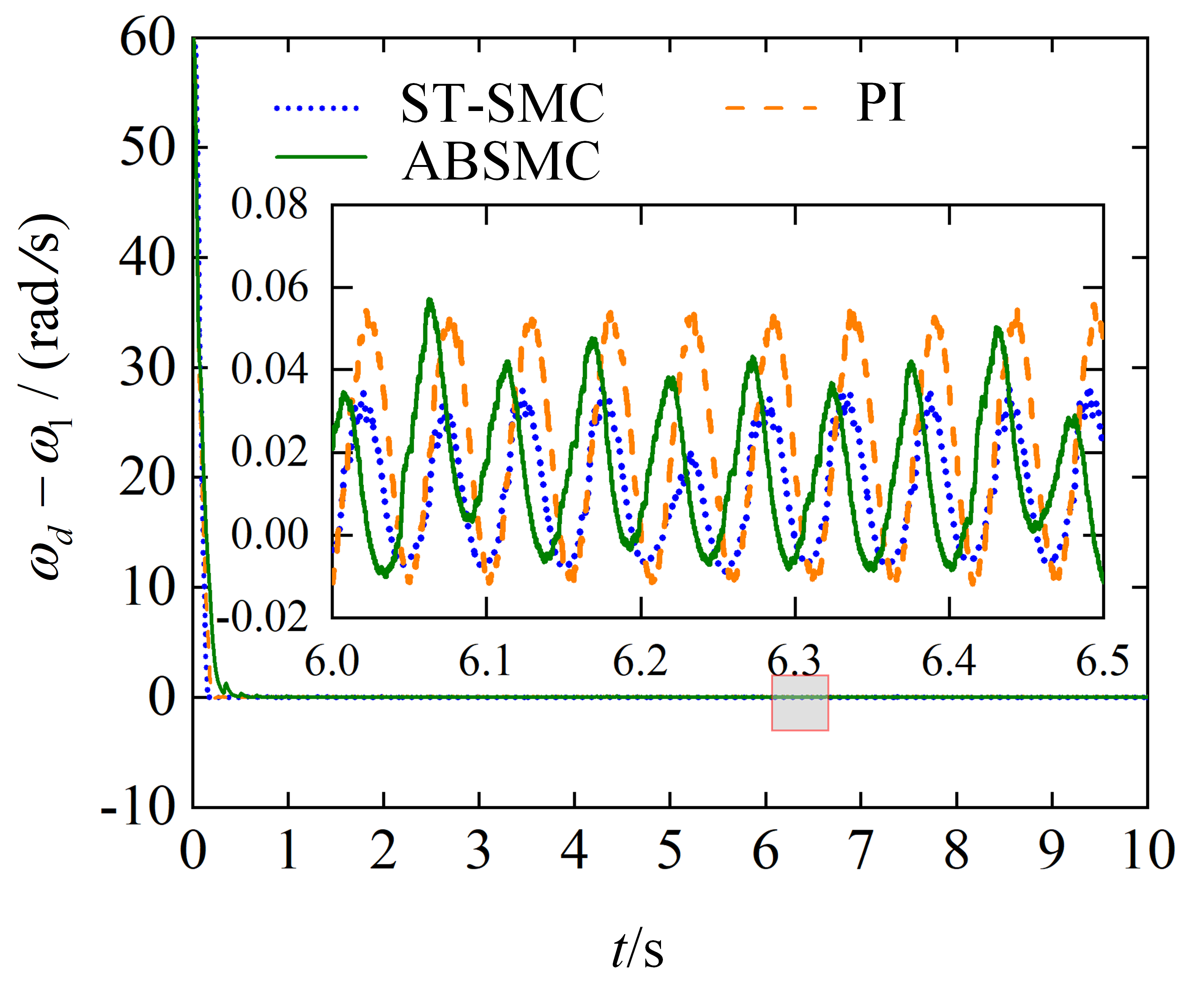

Supplement: S8 Fig — (ZIP) [file pone.0294726.s008.zip › (b).tif]

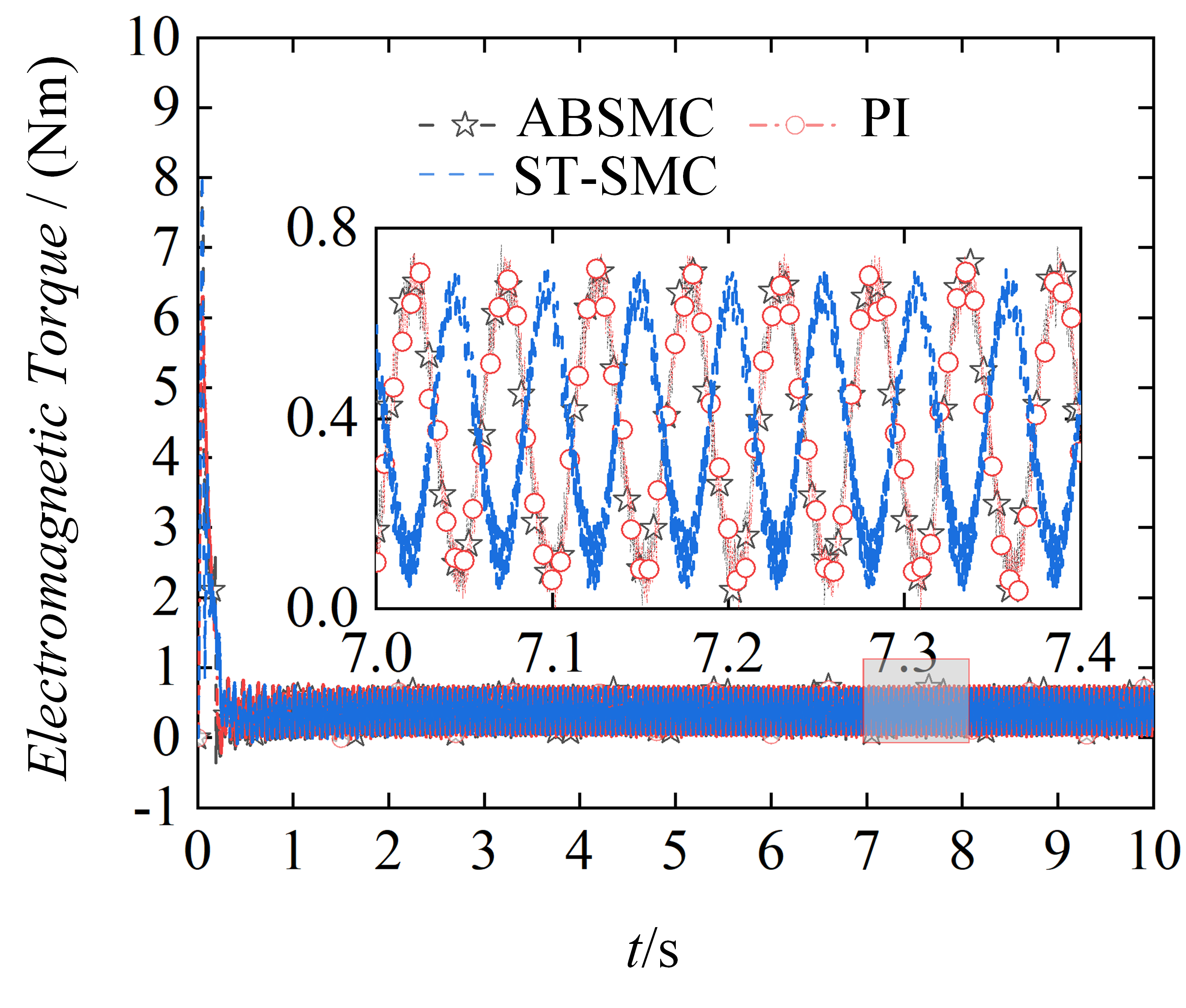

Supplement: S8 Fig — (ZIP) [file pone.0294726.s008.zip › (c).tif]

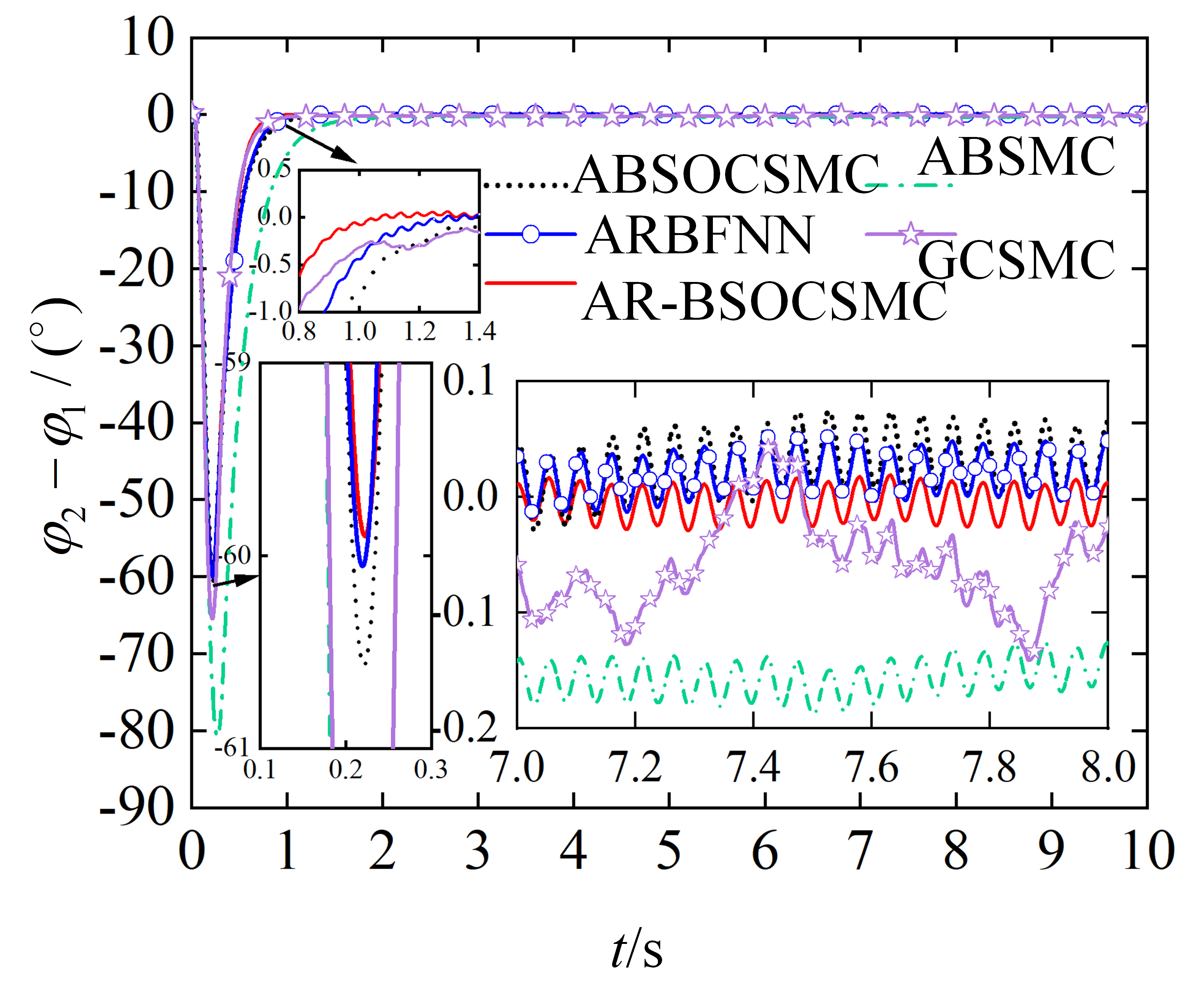

Supplement: S8 Fig — (ZIP) [file pone.0294726.s008.zip › (d).tif]

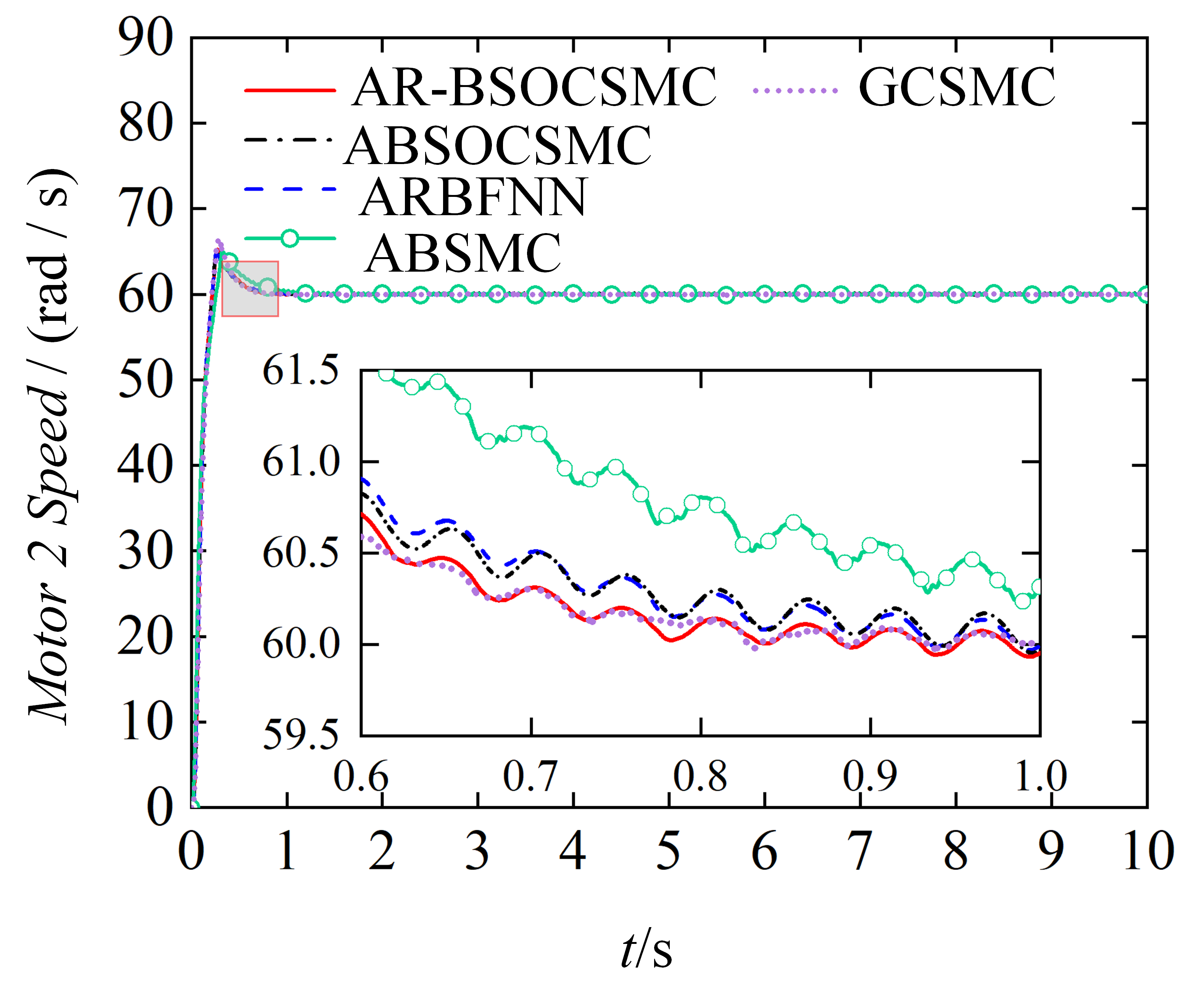

Supplement: S8 Fig — (ZIP) [file pone.0294726.s008.zip › (e).tif]

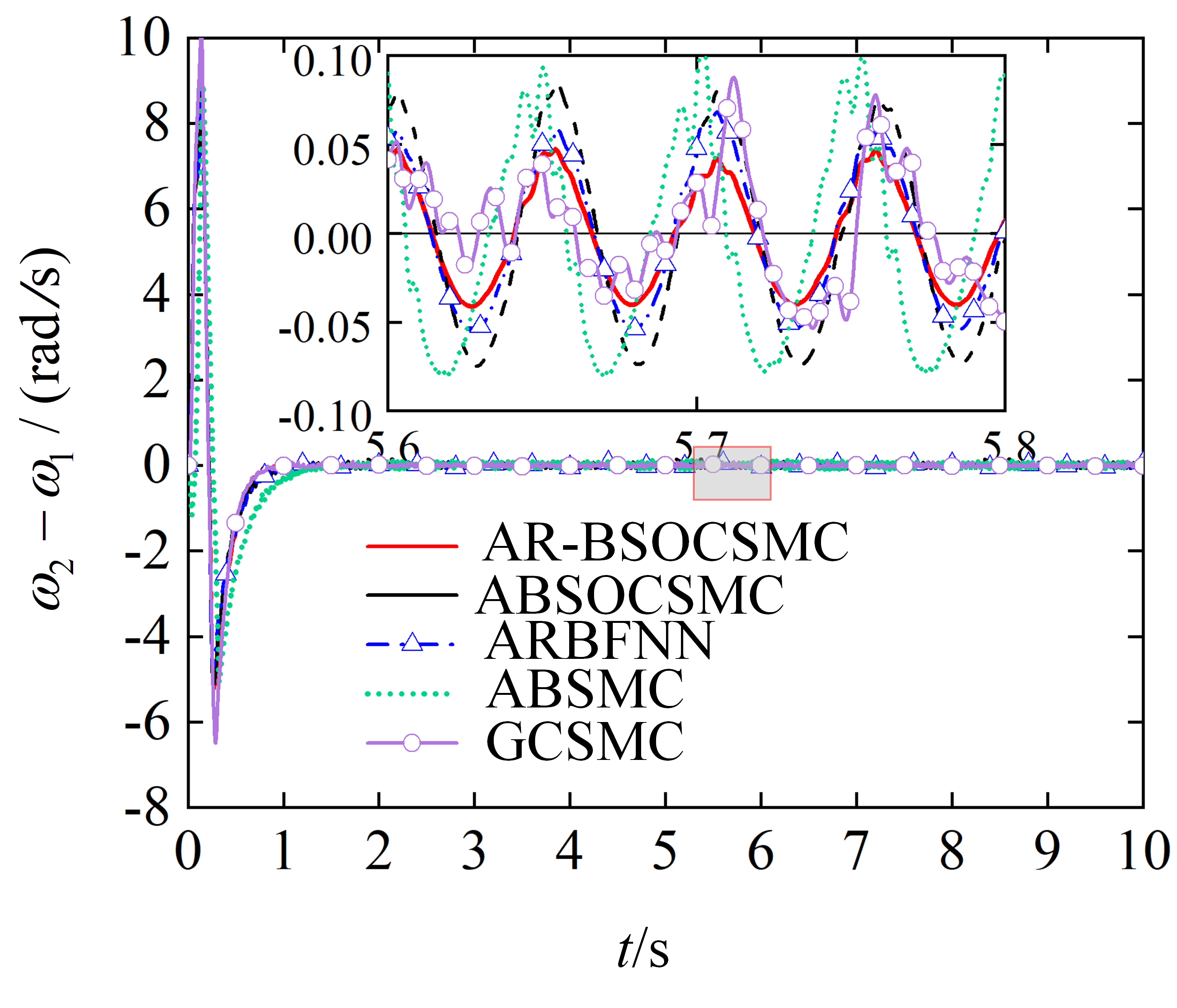

Supplement: S8 Fig — (ZIP) [file pone.0294726.s008.zip › (f).tif]

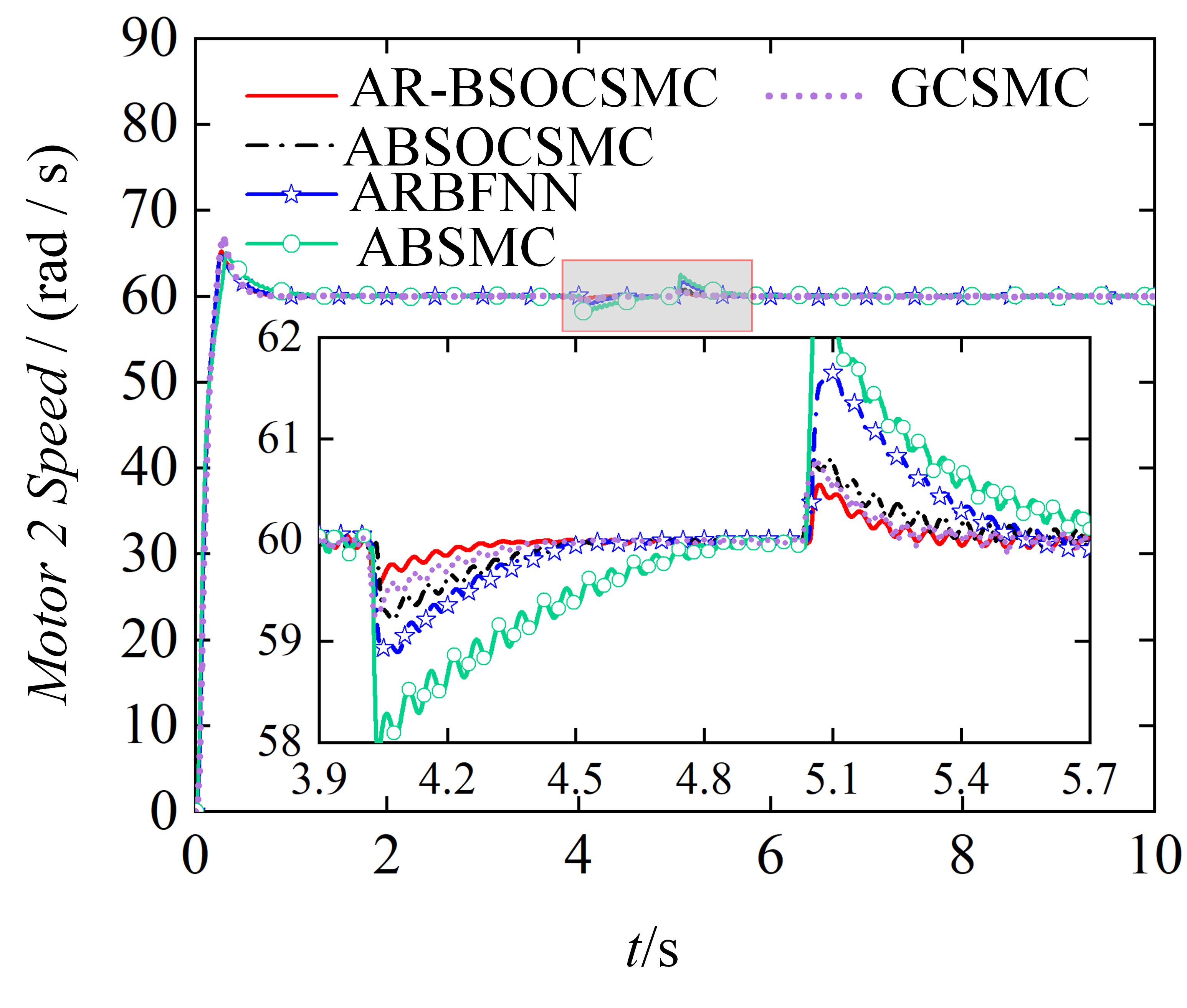

Supplement: S8 Fig — (ZIP) [file pone.0294726.s008.zip › (g).tif]

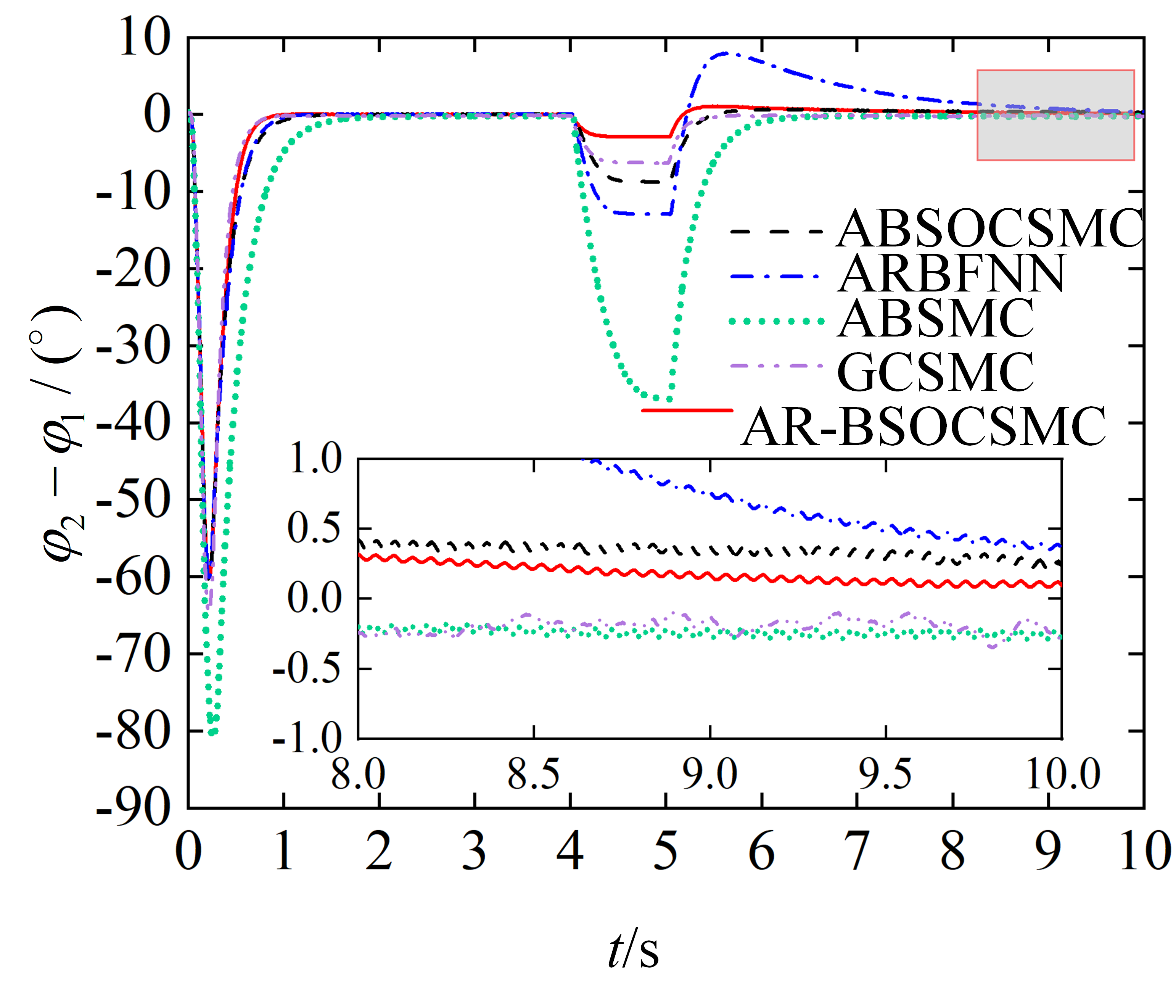

Supplement: S8 Fig — (ZIP) [file pone.0294726.s008.zip › (h).tif]
