# Supplementary material for: Controlled synchronization of a vibrating screen driven by two motors based on improved sliding mode controlling method
Source: PLoS One. 2023 Nov 21;18(11):e0294726. doi: 10.1371/journal.pone.0294726 (PMC10662758; doi:10.1371/journal.pone.0294726)
Supplement: S1 Table — (DOCX) [file pone.0294726.s009.docx]

| Parameters | Motor 1 | Motor 2 |
| --- | --- | --- |
| Power Rating *P*/kW | 1 | 1 |
| Number of polar pairs $n_{p}$ | 3 | 3 |
| Frequency rating $f_{0}$/Hz | 50 | 50 |
| Rated speed $n$/(r/min) | 950 | 950 |
| Stator resistance $R_{s}$/Ω | 5.75 | 5.4 |
| Rotor resistance $R_{r}$/Ω | 5.4 | 5.3 |
| Stator inductor $L_{s}$/H | 0.170 | 0.179 |
| Rotor inductor $L_{r}$/H | 0.170 | 0.179 |
| Coefficient of mutual inductance $L_{m}$/H | 0.115 | 0.125 |
| Given magnetic chain /Wb | 0.8 | 0.8 |
| Friction coefficient $f_{1,2}$/(N·s·m/rad) | 0.005 | 0.005 |
