# Supplementary material for: Controlled synchronization of a vibrating screen driven by two motors based on improved sliding mode controlling method
Source: PLoS One. 2023 Nov 21;18(11):e0294726. doi: 10.1371/journal.pone.0294726 (PMC10662758; doi:10.1371/journal.pone.0294726)
Supplement: S2 Table — (DOCX) [file pone.0294726.s010.docx]

| Parameters | Value |
| --- | --- |
| The quality of the shaking table and motors $m$/kg | 242 |
| The rotational inertia of the shaking table $J_{P}$/(kg·m^2^) | 43.5 |
| The spring stiffness in $x$ direction $k_{x}$/(N/m) | 129322 |
| The spring stiffness in $y$ direction $k_{y}$/(N/m) | 105334 |
| The spring stiffness in $\psi$ direction $k_{\psi}$/(N·m/rad) | 30715 |
| The damping coefficients in $x$ direction $f_{x}$/(N·s/m) | 615.5 |
| The damping coefficients in $y$ direction $f_{y}$/(N·s/m) | 618 |
| The damping coefficients in $\psi$ direction $f_{\psi}$/(N·s·m/rad) | 180.2 |
| The distance between $o$ and  $o_{1}$ $l_{1}$/m | 0.3 |
| The distance between $o$ and  $o_{2}$ $l_{2}$/m | 0.3 |
| The position angles of ER1 $\theta_{1}$/ (°) | 30 |
| The position angles of ER2 $\theta_{2}$/ (°) | 150 |
| The quality of ERs $m_{1}$/kg | 4 |
| Rotational radius of ERs $r$/m | 0.05 |
